# Live-Cell NanoBRET Assay to Measure AKT Inhibitor Binding to Conformational States of AKT

**DOI:** 10.1021/acschembio.5c00213

**Published:** 2025-07-09

**Authors:** Jeremy W. Harris, Flávio Antônio de Oliveira Simões, Erin N. Ryerson, William M. Marsiglia

**Affiliations:** Department of Genetics, The University of Alabama at Birmingham, Birmingham, Alabama 35294, United States

## Abstract

AKT is the main protein kinase of the PI3K-AKT pathway, interacting with over one hundred protein partners to facilitate cellular processes that allow cancer cells to survive and proliferate. It is an attractive target due to its control over many cellular outputs. However, ATP-competitive and allosteric AKT inhibitors have performed poorly in clinical trials. AKT inhibitor interactions with AKT are multifaceted and influence the catalytic activity of AKT, its conformation, its ability to interact with binding partners, and its phosphorylation state. Therefore, a better understanding of how these inhibitors influence these parameters is needed, especially in a cellular context. Using a live-cell NanoBRET target engagement assay to query the binding of AKT inhibitors to all isoforms of AKT, we found that ATP-competitive inhibitors bind similarly across all three isoforms and allosteric inhibitors bind more heterogeneously. Further, assaying gain-of-function pathological mutants and myristoylated active versions of all AKT isoforms revealed that T308 phosphorylation enhances the binding of ATP-competitive inhibitors. We found that this phosphorylation is a good indicator of cell viability sensitivity to ATP-competitive inhibitors when comparing effects on known resistant and sensitive triple-negative breast cancer cell lines. Taken together, these findings are useful for screening new AKT inhibitors, and these findings represent important considerations in developing the next generation of AKT inhibitors.

## Graphical abstract

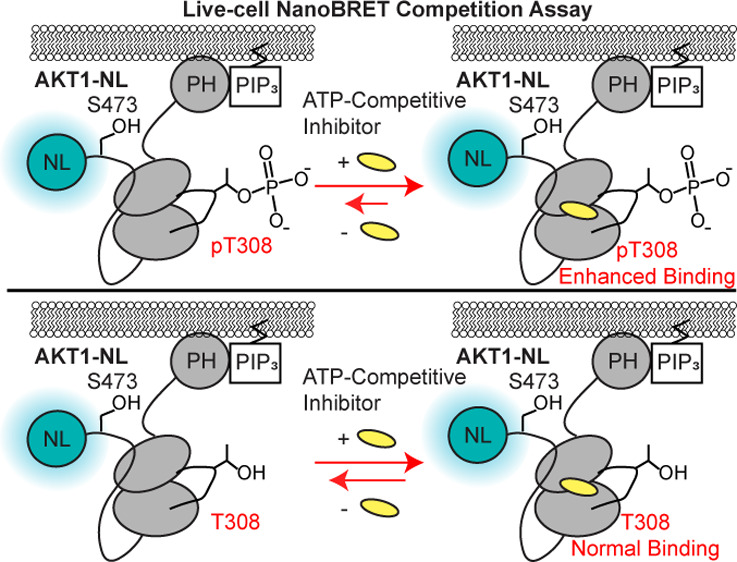

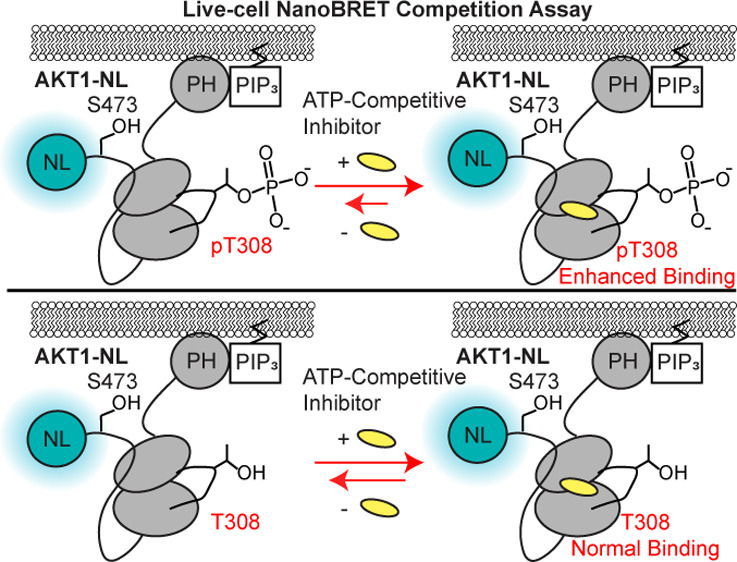

## Introduction

AKT is a critical protein kinase that interacts with more than 100 binding partners to facilitate cellular growth, proliferation, survival, and metabolism. There are three isoforms of AKT (1–3), each containing an N-terminal pleckstrin homology (PH) domain, kinase domain, and C-terminal regulatory tail (note that the use of “AKT” refers to all three isoforms). In its inactive conformation, each isoform resides in the cytoplasm and other cellular compartments, including the mitochondria and nucleus. Here, its PH domain is associated with its kinase domain to prevent interaction with binding partners.^1^ Upon generation of PIP_3_ by PI3K, the PH domain of AKT translocates to the plasma membrane (Figure 1A). In this active confirmation, the kinase domain is exposed for phosphorylation at residue T308/T309/T305 (AKT1/2/3) by PDK1 and at S473/S474/S472 (AKT1/2/3) by the mTORC2 complex. These phosphorylation events fully activate AKT’s catalytic activity, allowing it to regulate binding partner activity through subsequent phosphorylation events.

**Figure 1:**
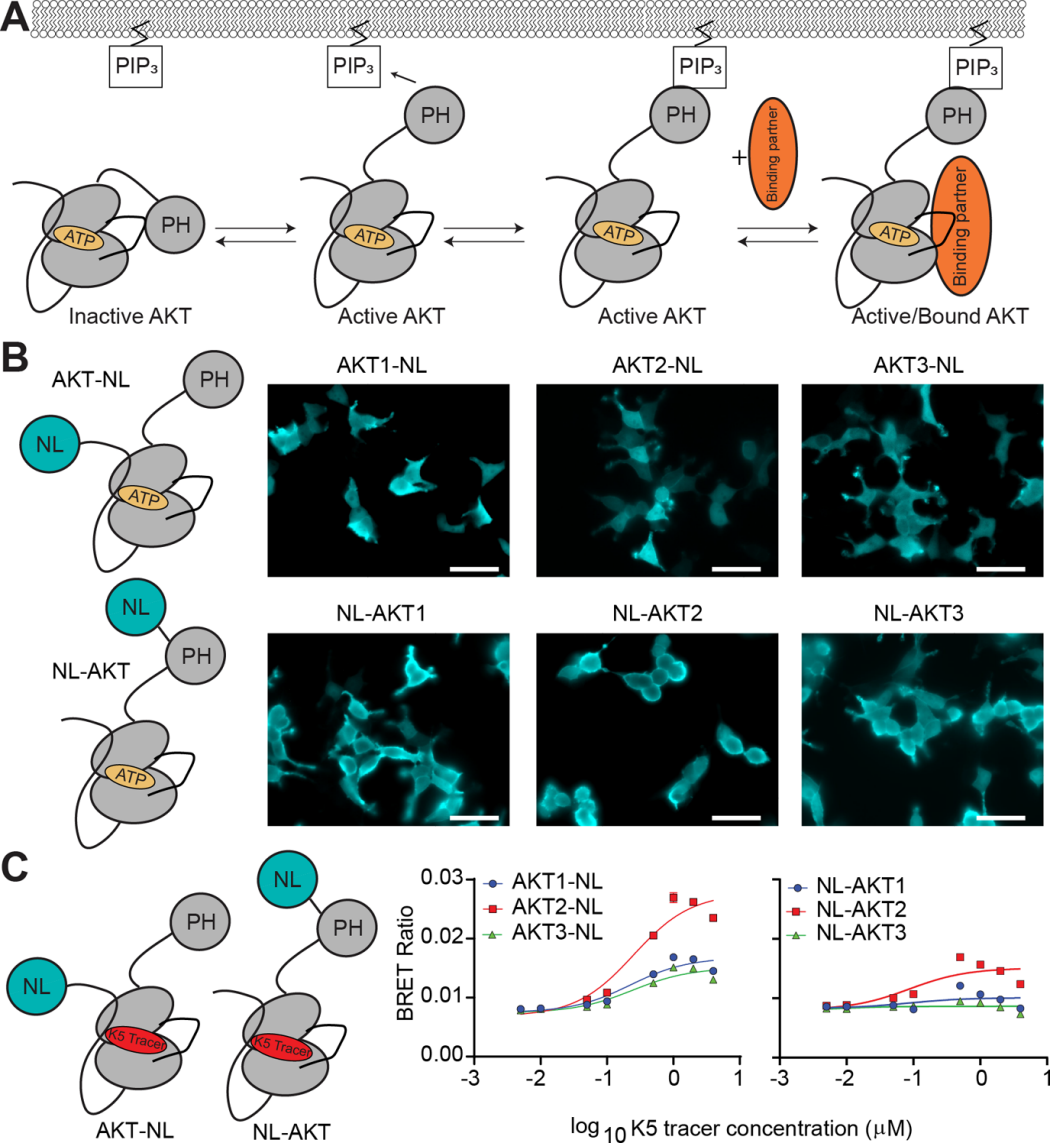
Optimum placement of NanoLuciferase on all AKT isoforms. (A) Schematic of AKT activation in the presence of PIP_3_. (B) Bioluminescent imaging of AKT-NL and NL-AKT constructs transfected into HEK293T cells. Scale bar = 50 μm. (C) Placement of Nanoluc on the C-terminus of all AKT isoforms yields a higher BRET signal than N-terminal placement. Each point for (C) represents an average of three separate transfections (biological replicate, *n* = 3). Each biological replicate had one technical replicate. Error bars represent the SEM of all of the biological replicates.

In cancer, AKT is aberrantly activated through PI3K-AKT pathway stimulation by upstream receptor tyrosine kinases and/or PI3K/Ras mutations, and AKT gene amplification.^2–5^ AKT mutations are less common but enhance the oncogenesis of breast, uterine, colorectal, and prostate cancers.^6,7^ As such, AKT stands as an attractive drug target. Directly targeting AKT to inhibit PI3K-AKT pathway stimulation or increased kinase activity imparted by AKT mutations has promising implications for controlling oncogenic cellular processes. Two main classes of AKT kinase inhibitors are ATP-competitive (type 1) and allosteric (type 4). ATP-competitive inhibitors inhibit AKT through competition with ATP for AKT’s active site and putatively promote the active conformation of AKT upon binding.^8^ A-443654 is a notable example that enhances membrane association and hyperphosphorylation of T308 and S473.^9^ Allosteric inhibitors, such as MK-2206 and ARQ-092 (miransertib), inhibit AKT by stabilizing its inactive conformation. Despite multiple modes of inhibition, AKT inhibitors have shown a lack of efficacy in treating cancer.^10,11^

A contributing factor underlying the AKT inhibitor ineffectiveness relates to how kinase inhibitors are developed. These methods include cell viability assays that indirectly report on inhibitor binding and cell-free in vitro drug-binding assays using purified protein or lysates. Previous literature indicates that on-target binding in a cell-free system may not fully recapitulate binding in cells because of factors including membrane permeability, drug metabolism, target phosphorylation state, etc.^12,13^ In addition, since AKT inhibitors influence the conformational equilibrium of AKT between inactive and active conformations to affect its phosphorylation state and availability to binding partners, it is essential to know the affinity of AKT inhibitors toward these conformations. Obtaining this information in a cellular context is key to providing the most relevant insight for identifying and generating new inhibitors that target specific AKT isoforms and conformations prevalent in disease.

Here, we developed a NanoBRET assay to measure the binding affinity of ATP-competitive and allosteric inhibitors for different conformational states and activating mutants of AKT. We also explored the role of T308 and S473 phosphorylation on AKT inhibitor binding and the ability to use this phosphorylation as a predictor of cell viability in triple-negative breast cancer. These results represent a platform for developing future inhibitors that focus on the inhibition of specific conformations of AKT.

## Results and Discussion

### NanoBRET Assay to Measure AKT Inhibitor Binding across AKT Isoforms

To quantify AKT inhibitor binding across all isoforms in a cellular context and correlate these results to clinical effectiveness, we performed NanoBRET competition assays. In this live-cell assay, bioluminescence resonance energy transfer (BRET) produced by the interaction of a transfected NanoLuciferase (NL)-tagged protein of interest and a protein-bound Bodipy-containing tracer molecule is dose-dependently competed out with a drug. To develop an assay for AKT, we first empirically determined if an N-terminal or C-terminal placement of NL on each AKT isoform would be optimal for BRET signal (i.e., orientation where the NL is closest to the tracer). These experiments were performed under serum-starved conditions in Opti-MEM to reduce background phosphorylation and promote an inactive population of each isoform. Bioluminescent imaging of all overexpressed AKT isoforms with N-terminal and C-terminal placement showed localization throughout the cell (Figure 1B). BRET signal buildup curves (tracer EC_50_) generated using the ATP-competitive K5 tracer from Promega indicated that the best placement (highest BRET signal) for the NL on AKT isoforms was the C-terminus (Figures 1C and S1A). The highest BRET signal of the AKT isoforms was observed for AKT2, and the binding EC_50_ values for the K5 tracer were similar across all AKT isoforms (EC_50_: AKT1-NL 0.219 ± 0.006 μM; AKT2-NL 0.21 ± 0.02 μM; AKT3-NL 0.19 ± 0.03 μM; “±” indicates the standard error of the mean (*n* = three biological replicates)).

We next characterized the binding of three representative ATP-competitive inhibitors (ipatasertib, capivasertib, and A-443654) and three representative allosteric inhibitors (MK-2206, miransertib, and inhibitor VIII) to AKT1-NL, AKT2-NL, and AKT3-NL (Figures 2A,B, and S1B–D). Although the K5 tracer is based on an ATP competitive inhibitor, we were confident that our assay would work for allosteric inhibitors because allosteric inhibitor binding displaces the DFG-loop into a conformation that is not compatible with ATP and K5 tracer binding (Figure S1E).

**Figure 2:**
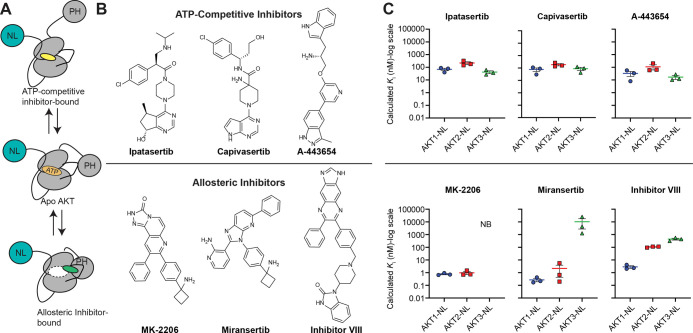
Characterization of ATP-competitive and allosteric AKT inhibitors on all AKT isoforms. (A,B) Schematic of binding modes and structures for ATP-competitive (ipatasertib, capivasertib, and A443654) and Allosteric (MK-2206, miransertib, and inhibitor VIII) AKT inhibitors. The dotted oval in the allosteric-bound diagram represents the ATP-binding pocket. (C) Calculated *K*_i_ values for ATP-competitive (top) and allosteric inhibitors. Note that “NB” indicated no binding. Each point for (C) represents a separate transfection (biological replicate, *n* = 3). Each biological replicate has one technical replicate. Error bars represent the SEM of the biological replicates.

We observed that the ATP-competitive inhibitors bound similarly across all three isoforms (Table 1, and Figure 2C). MK-2206 and Miransertib bound better to AKT1 and AKT2 than to AKT3. Inhibitor VIII preferred AKT1 over AKT2 and AKT3. The reduced potency of allosteric inhibitors for AKT3 is consistent with literature showing that the upregulation of AKT3 is associated with MK-2206 resistance in breast cancer^14^ and higher kinase activity of AKT3 relative to AKT1 and 2 in multiple cell lines when exposed to MK-2206.^15^ The isoform differences observed for the allosteric inhibitors could be explained by differences in the primary sequences among the isoforms that make up the inhibitor binding site.^16,17^ It should be noted that the differences in calculated *K*_i_ values between AKT1 and AKT3 for allosteric inhibitors are orders of magnitude larger than values previously reported using other biochemical methods but follow the same potency trends.^15,18^

**Table 1:** Summary of Calculated *K*_i_ Values for AKT1-NL, AKT2-NL, and AKT3-NL ± the Standard Error of the Mean (*n* = Three Biological Replicates)^a^

AKT inhibitor	AKT1-NL calculated *K*_i_ (nM)	AKT2-NL calculated *K*_i_ (nM)	AKT3-NL calculated *K*_i_ (nM)
ipatasertib	68.7 ± 14.8	216.2 ± 55.7	41.9 ± 10.6
capivasertib	76.0 ± 23.9	186.0 ± 39.2	87.8 ± 24.7
A-443654	32.7 ± 14.1	108.2 ± 46.7	16.6 ± 5.5
MK-2206	0.76 ± 0.09	0.97 ± 0.25	NB
miransertib	0.27 ± 0.08	2.1 ± 1.7	10,225 ± 7478
inhibitor VIII	2.8 ± 0.7	106.3 ± 6.6	438.7 ± 72.3

a NB indicates no binding.

We also performed bioluminescent imaging on AKT1-NL, AKT2-NL, and AKT3-NL transfected into HEK293T cells treated individually with all AKT inhibitors for 2 h (Figure S1F). We observed that for AKT1-NL and AKT2-NL compared to DMSO-treated cells, ipatasertib and capivasertib induced the movement of some AKT to the cell membrane. This effect was not observed for AKT3-NL. A-443654 induced movement of all isoforms to the cell membrane and nucleus. MK-2206, miransertib, and inhibitor VIII did not induce AKT localization changes for any isoform. This result is consistent with the mechanism of action of allosteric inhibitors that prevents the PH domain from interacting with membranes.

### Characterization of AKT Inhibitor Binding to Pathogenic AKT Mutants

We were curious to see how the same set of inhibitors bound to pathological gain-of-function mutants that shift AKT toward a more active population. Mutations of AKT are found in breast, prostate, colorectal, glioma, endometrial, bladder, lung, and melanoma cancers.^19–22^ AKT1 has a greater frequency of mutations than AKT2 and AKT3.^23^ These mutations are generally located near the interface between the PH and kinase domains (L52R and D323H), the PIP_3_ binding pocket (E17K), and the drug-binding pocket of allosteric inhibitors (Q79K and W80R) (Figure 3A). In the context of AKT1-NL, we performed bioluminescent imaging of E17K, L52R, Q79K, W80R, and D323H (Figure S2A). We also imaged the E17K mutant in the context of AKT2-NL and AKT3-NL (Figure S3A). For AKT1-NL, E17K, L52R, Q80R, and D323H promoted the association of AKT1 at the cell membrane. Q79K remained in the cytosol but not the nucleus. In the contexts of AKT2-NL and AKT3-NL, the E17K mutant remained mainly in the cytosol.

**Figure 3:**
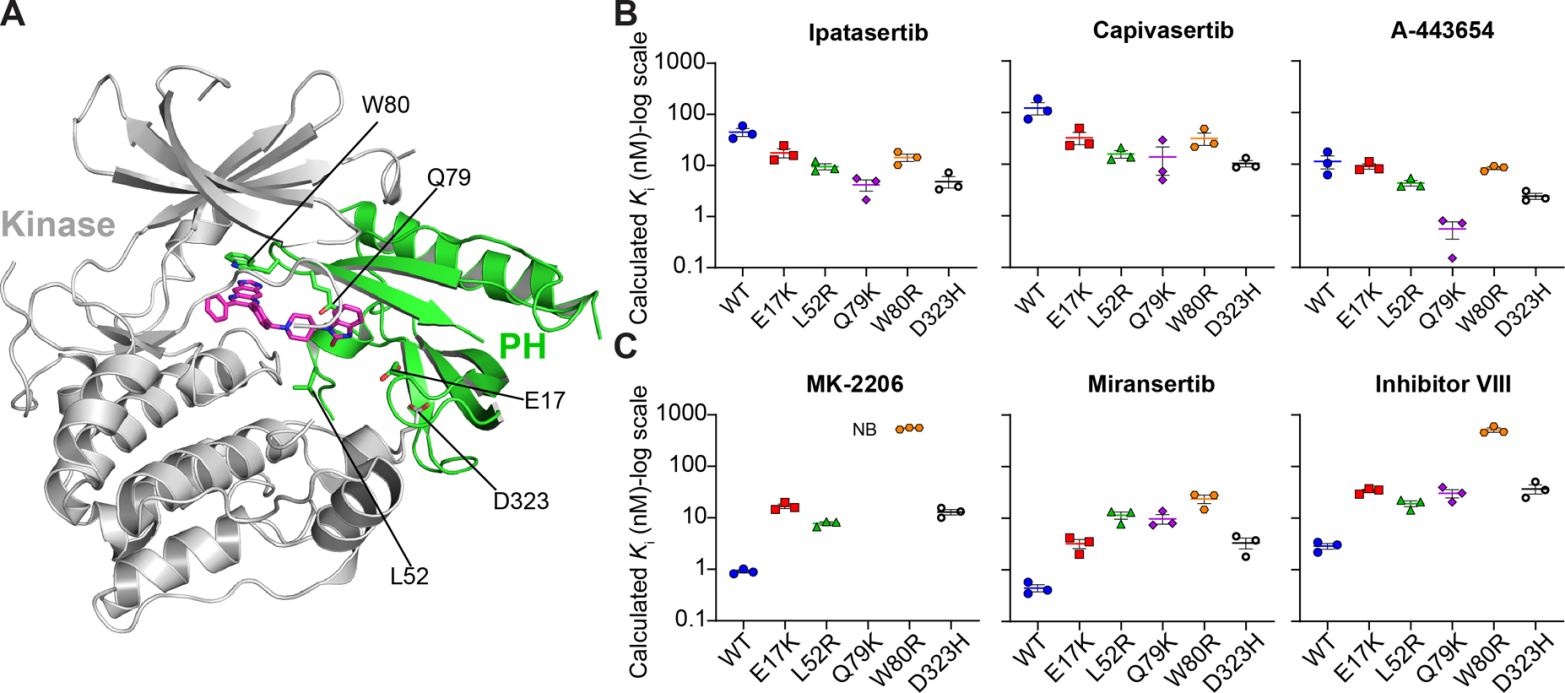
Comparison of AKT inhibitor binding on pathogenic mutations. (A) Structure of AKT2 bound to inhibitor VIII (PDB ID: 3O96) highlighting the location of pathogenic AKT mutations. (B) Calculated *K*_i_ values for ATP-competitive inhibitors on pathogenic mutants. (C) Calculated *K*_i_ values for allosteric inhibitors for pathogenic mutants. Note that “NB” indicates no binding. Each point for (B,C) represents a separate transfection (biological replicate). Each biological replicate has one technical replicate. Error bars represent the SEM of the biological replicates.

We next quantified the binding of ATP-competitive and allosteric inhibitors to these pathogenic mutants. Compared to wild-type (WT) AKT1-NL, we observed enhanced binding of ATP-competitive inhibitors to all mutants (Figures 3B and S2B–E). All allosteric inhibitors bound worse to all mutants relative to WT-AKT1-NL (Figures 3C and S2B–E). Notably, no binding of MK-2206 was observed to the Q79K mutant, a key residue that is part of the MK-2206 binding site. Miransertib and inhibitor VIII, however, bound better to Q79K, highlighting mutant-specific inhibitor selectivity. There was no significant difference in ATP-competitive inhibitor binding between WT and E17K versions of AKT2-NL and AKT3-NL (Figure S3B–F). MK-2206 did not bind to AKT3-NL or AKT3-E17K-NL, and miransertib did not bind to AKT3-E17K-NL. The weaker binding of allosteric inhibitors to AKT1 mutants can be explained through a combination of mutation location (i.e., near the inhibitor binding site) and destabilization of the kinase-PH domain interface that makes up the inhibitor binding site.^19^

### Residue T308 in AKT1 Is Important for the Binding of ATP-Competitive AKT Inhibitors

It was puzzling to understand why ATP-competitive inhibitors bound more tightly to some pathological mutants. Since these mutants have been shown to increase AKT activity by enhancing T308 and S473 phosphorylation,^24^ we reasoned that changes in phosphorylation and conformation from inactive to active states may influence inhibitor binding. It was previously shown that unstimulated AKT primarily resides in the cytoplasm inactive and unphosphorylated until increased PIP_3_ levels are present.^25^ To achieve an active AKT population characterized by increased phosphorylation and membrane association, we created N-terminally myristoylated AKT1–3-NL constructs (Figure 4A). AKT myristoylation is commonly used to keep AKT membrane-associated and available for phosphorylation by PDK1 and mTORC2.^26,27^ We decided to use myristoylation instead of treatment with a PI3K-AKT stimulating hormone such as IGF1 because of transient time-dependent effects on phosphorylation that complicates the reproducibility of our NanoBRET assay.^28,29^ Bioluminescent imaging of Myr-AKT1-NL, Myr-AKT2-NL, and Myr-AKT3-NL constructs confirmed that each isoform was associated at the cell membrane (Figure S4A).

**Figure 4:**
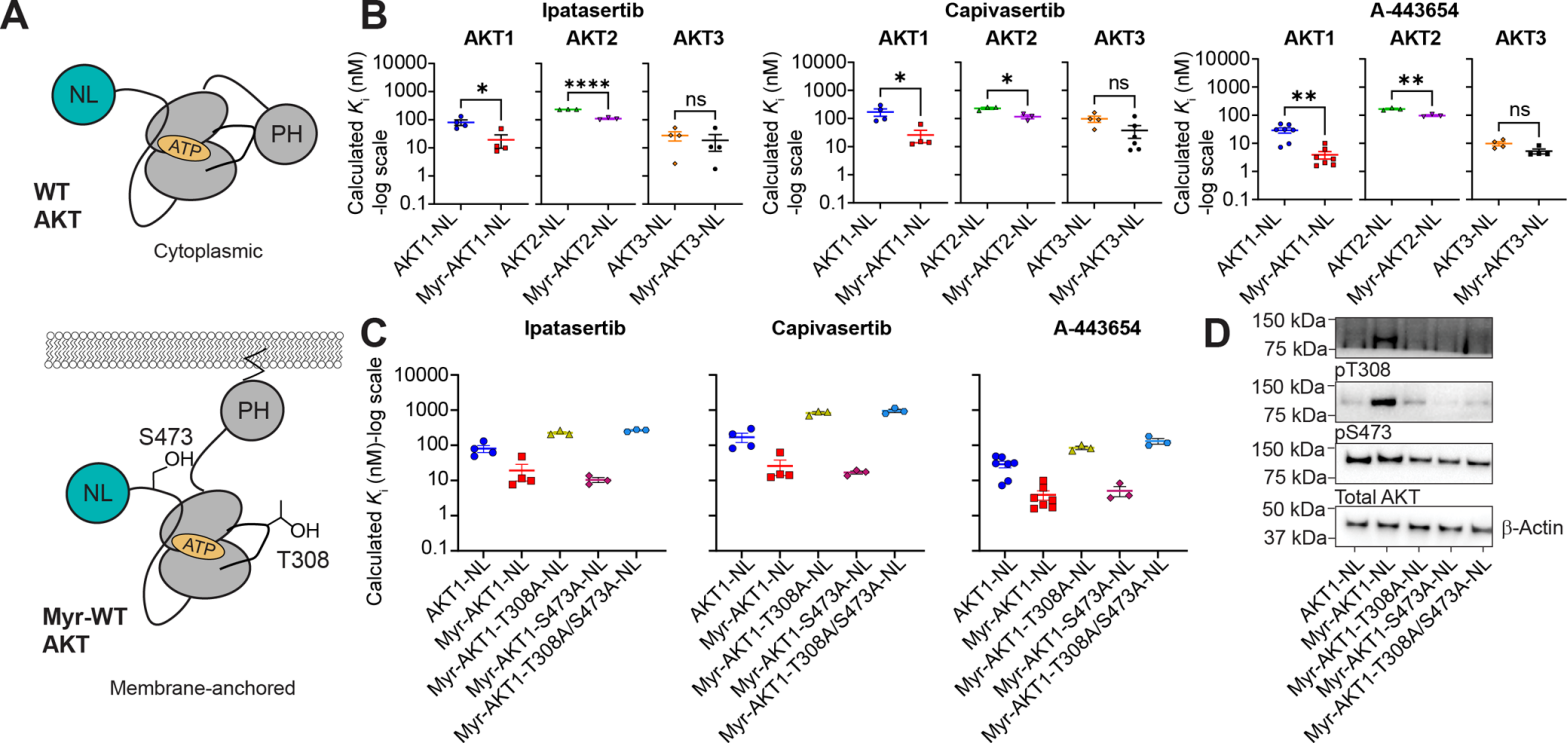
Influence of residues T308 and S473 on AKT inhibitor binding. (A) Schematic showing WT-AKT-NL (top) and Myristoylated AKT-NL constructs (bottom). Phosphorylatable residues T308 and S473 are shown by using AKT1 numbering. (B) Calculated *K*_i_ comparison of ATP-competitive binding to WT and myristoylated versions of each AKT isoform. Ipatasertib: AKT1-NL vs Myr-AKT1-NL, *P* = 0.286, *U* = 0, two-tailed Mann–Whitney *U* test; AKT2-NL vs Myr-AKT2-NL, *P* = <0.001, *t* = 20.41, df = 4, two-tailed unpaired *t*-test; AKT3-NL vs Myr-AKT3-NL, *P* = 0.5749, *t* = 0.5928, df = 6, two-tailed unpaired *t*-test. Capivasertib: AKT1-NL vs Myr-AKT1-NL, *P* = 0.286, *U* = 0, two-tailed Mann–Whitney *U* test; AKT2-NL vs Myr-AKT2-NL, *P* = 0.0134, *t* = 4.231, df = 4, two-tailed unpaired *t*-test; AKT3-NL vs Myr-AKT3-NL, *P* = 0.1143, *U* = 4, two-tailed Mann–Whitney *U* test. A-443654: AKT1-NL vs Myr-AKT1-NL, *P* = 0.0023, *U* = 2, two-tailed Mann–Whitney *U* test; AKT2-NL vs Myr-AKT2-NL, *P* = 0.0033, *t* = 6.294, df = 4, two-tailed unpaired *t*-test; AKT3-NL vs Myr-AKT3-NL, *P* = 0.1143, *U* = 2, two-tailed Mann–Whitney *U* test. (C) Comparison of calculated *K*_i_ values of ATP-competitive inhibitor binding to WT-AKT1-NL and versions of AKT that cannot be phosphorylated at specific residues to determine the source of enhanced ATP-competitive inhibitor binding to Myr-AKT1-NL. *K*_i_ values were calculated via the Cheng-Prusoff equation using measured K5 EC_50_ values in Figures S1A and S4A, and apparent IC_50_ values in Figure S4A. Note that the data for AKT1-NL and Myr-AKT1-NL in (B,C) are the same due to how the data were collected. (D) Western blot analysis confirming the phosphorylation states of T308 and S473 in each construct. *P < 0.05; **P < 0.01; ****P < 0.0001. Each point for (B,C) represents a separate transfection (biological replicate). Each biological replicate has one technical replicate. Error bars represent the SEM of the biological replicates.

We then measured K5 tracer binding across myristoylated isoforms and observed that it was similar to the nonmyristoylated versions of each isoform (K5 EC_50_: AKT1-NL 0.219 ± 0.006 μM; Myr-AKT1-NL 0.18 ± 0.02 μM; AKT2-NL 0.21 ± 0.02 μM; Myr-AKT2-NL 0.124 ± 0.007 μM; AKT3-NL 0.19 ± 0.03 μM; Myr-AKT3-NL 0.16 ± 0.01 μM; “±” indicates the standard error of the mean (*n* = three biological replicates)) (Figures S1A and S4B). Characterization of ATP-competitive and allosteric inhibitors on nonmyristoylated and myristoylated versions of AKT1–3-NL (Figures 4B and S4C–N) revealed that relative to the nonmyristoylated protein, there was a ∼6-fold enhancement in the potency of ATP-competitive inhibitors to AKT1-NL vs Myr-AKT1-NL on average. A smaller change between AKT2-NL and Myr-AKT2-NL (∼2-fold) and no significant change in AKT3-NL vs Myr-AKT3-NL were also observed. These results suggest that ATP-competitive inhibitors are isoform-selective in their preference for active and membrane-associated AKT.

Since the AKT1 isoform showed a larger change in ATP-competitive inhibitor binding affinity than the other isoforms, we aimed to determine the influence of T308 and S473 phosphorylation sites in its myristoylated context. To do this, we mutated each site separately (T308A or S473A) and in combination (T308A/S473A) to alanine (Figures 4C and S4E). Indeed, Western blot analysis of AKT1-NL, Myr-AKT1-NL, Myr-AKT1-T308A-NL, Myr-AKT1-S473A-NL, and Myr-AKT1-T308A/S473A-NL revealed that alanine mutation prevented the phosphorylation of its corresponding residue (Figures 4D and S5). We observed that the mutation of T308 (Myr-AKT1-T308A-NL) alone or in combination with S473 (Myr-AKT1-T308A/S473A-NL) was sufficient to reverse the increase in binding potency of ATP-competitive inhibitors and resembled binding to AKT1-NL. The binding affinities of ATP-competitive inhibitors to AKT1 harboring the S473A mutation matched more closely to the Myr-AKT1-NL construct. The allosteric connection between the ATP binding site and T308 has been previously shown where the presence of ATP or A-443654 promotes the resistance of pT308 to dephosphorylation through long-range interactions mediated by N-lobe to C-lobe closure. This closure places residues on the αC-helix in position to sequester pT308 away from solvent.^30^ These results are the first representations of this allostery in the context of live-cell target engagement. Therefore, we conclude that residue T308 but not S473 allosterically enhances the binding of ATP-competitive inhibitors to AKT.

The same NanoBRET experiments were then performed using allosteric inhibitors, and worse binding was observed for all myristoylated constructs regardless of mutation relative to AKT1-NL (Figure S4E). In general, myristoylation reduced calculated *K*_i_ values for each allosteric inhibitor 10–100-fold. This result indicates that myristoylation makes it more difficult for allosteric inhibitors to stabilize the inactive form of AKT. The weaker binding of allosteric inhibitors, combined with stronger binding to ATP-competitive inhibitors, represents a characteristic inhibitor “profile” of Myr-AKT-NL constructs.

To ensure that changes in ATP-competitive inhibitor binding affinity were not due to generally mutating an amino acid, we mutated residue C344S on Myr-AKT1-NL (Figure S4E). This residue is located at the C-terminal end of the αF helix in the kinase C-lobe, away from residues that could influence kinase conformation, inhibitor binding sites, and catalytic residues. Western blot analysis of T308 and S473 phosphorylation in the Myr-AKT1-C344S-NL construct matched closely with that in Myr-AKT1-NL and confirmed a minimal effect of the mutation (Figure S5). We also observed from our NanoBRET assay that the Myr-AKT1-C344S-NL mutant retained the ATP-competitive inhibitor binding affinity enhancement displayed by the Myr-AKT1-NL construct.

### The Starting Population of pT308 Is Important for ATP-Competitive Inhibitor Binding

In light of determining the role of T308 in enhancing the binding of ATP-competitive inhibitors, we wanted to see if we could shift the populations of phosphorylated T308 and thus ATP-competitive binding using a PDK1 inhibitor (BX-795). In other words, we were curious to see if BX-795 would influence ATP-competitive inhibitor binding to AKT1-NL (low pT308) and Myr-AKT1-NL (high pT308) (Figure 5A). To test this, we performed a steady-state NanoBRET competition assay for A-443654 in the presence of a constant concentration of BX-795 (1 μM). The concentration of BX-795 was determined empirically from dose–response curves of BX-795 on AKT1-NL and Myr-AKT1-NL (Figures 5B and S6A). Notably, some binding of BX-795 to AKT1-NL and Myr-AKT1-NL was observed at concentrations higher than 1 μM. We also confirmed that BX-795 substantially inhibits PDK1 at a 1 μM concentration by performing a competition assay using PDK1-NL (Calculated *K*_i_: 5.85 ± 0.42 nM; “±” indicates the standard error of the mean (*n* = three biological replicates); Figure S6C). NanoBRET competition assays of A-443654 in the presence and absence of BX-795 for AKT1-NL and Myr-AKT1-NL revealed that A-443654 binding to AKT1-NL was unaffected by BX-795 and that A-443654 bound more weakly to Myr-AKT1-NL in the presence of BX-795 (Figures 5C and S6D–H). This reversal of ATP-competitive inhibitor binding enhancement agrees well with the effect of the T308A-Myr-AKT1-NL mutant in Figure 4C. In addition, we were able to reverse the effect of BX-795 on Myr-AKT1-NL by overexpressing unlabeled PDK1 (Figure S6D–F).

**Figure 5:**
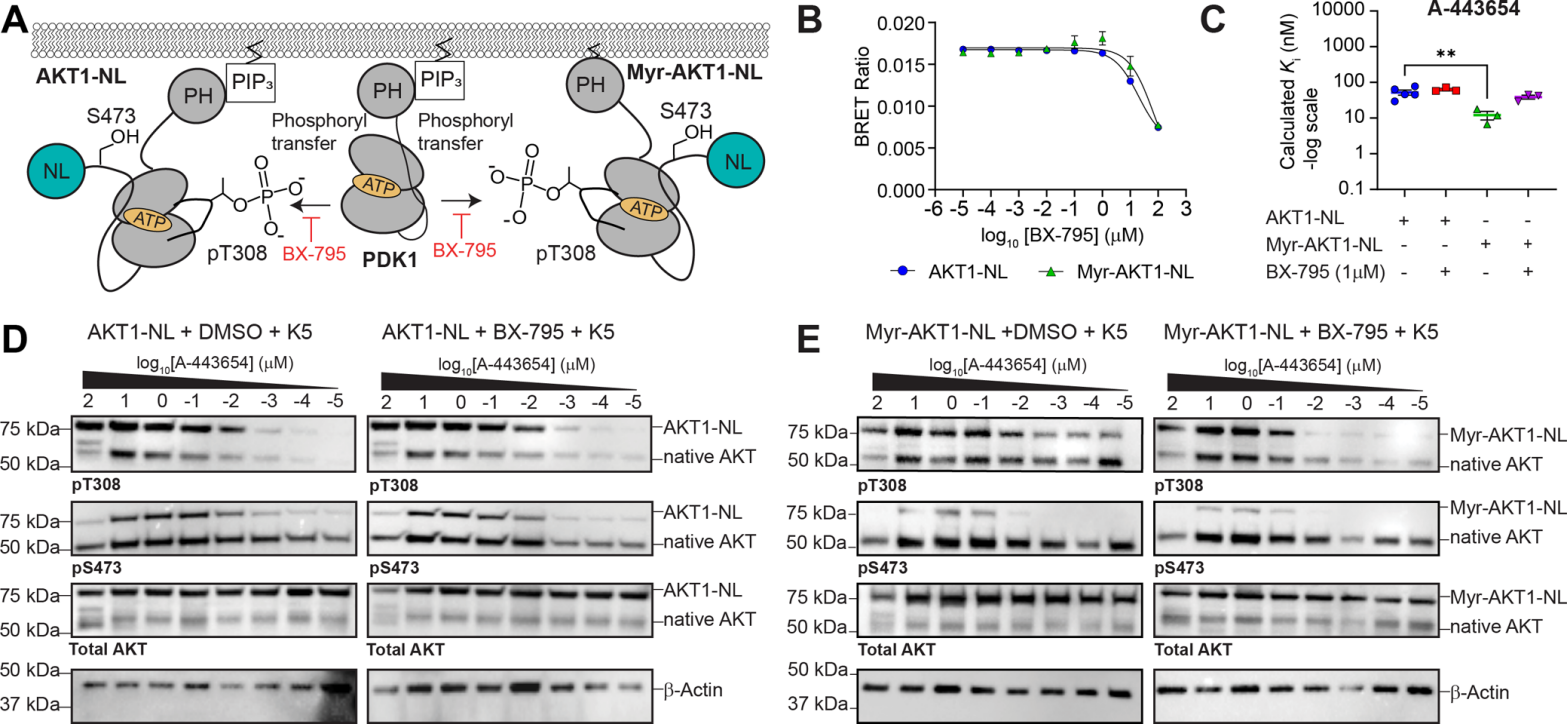
Pharmacological modulation of AKT phosphorylation and AKT inhibitor binding. (A) Schematic showing the role of BX-795 (PDK1 inhibitor) in blocking T308 phosphorylation on AKT1-NL and Myr-AKT1-NL. (B) Dose–response curves of BX-795 on AKT1-NL and Myr-AKT1-NL. (C) Calculated *K*_i_ values for A-443654 on AKT1-NL and Myr-AKT1-NL in the presence and absence of 1 μM BX-795. Data were analyzed by one-way ANOVA with Dunnett’s multiple comparison test (AKT1-NL vs AKT1-NL + 1 μM BX-795 adjusted *P* = 0.6201, AKT1-NL vs Myr-AKT1-NL adjusted *P* = 0.005, AKT1-NL vs Myr-AKT1-NL + 1 μM BX-795 adjusted *P* = 0.4152). Note that the final DMSO concentration is higher in all conditions relative (0.2% DMSO) to that in Figure 4B (0.1% DMSO) and may contribute to differences in calculated *K*_i_ values. Importantly, the trends are the same. (D) Western Blot analysis of AKT1-NL and AKT1-NL + 1 μM BX-795 in the presence of an A-443654 dose–response curve. (E) Western Blot analysis of Myr-AKT1-NL and Myr-AKT1-NL + 1 μM BX-795 in the presence of an A-443654 dose–response curve. *P < 0.05; **P < 0.01; ****P < 0.0001. Each point for (C) represents a separate transfection (biological replicate). Each biological replicate has one technical replicate. Error bars represent the SEM of the biological replicates.

Given that the addition of A-443654 induces AKT hyperphosphorylation, we aimed to quantify the contribution of T308 and S473 hyperphosphorylation on the steady-state NanoBRET-derived *K*_i_ values of A-443654 observed for AKT1-NL and Myr-AKT1-NL in the presence and absence of BX-795. We performed a Western blot analysis on a larger scale NanoBRET assay where 293T cells transfected with either AKT1-NL or Myr-AKT1-NL were separately incubated with each concentration of an A-443654 dose–response curve (100, 10, 1, 0.1, 0.01, 0.001, 0.0001, or 0.00001 μM), 1 μM K5 tracer, and either 1 μM of BX-795 or DMSO for 2 h at 37 °C. For AKT1-NL, we observed that A-443654 created a dose-dependent increase in T308 and S473 phosphorylation, which was unaffected by BX-795 (Figures 5D and S7A,B). Myr-AKT1-NL also showed a dose-dependent increase in T308 phosphorylation in the presence and absence of BX-795 (Figures 5D and S7C,D). However, the addition of BX-795 strongly reduced the level of phosphorylation of T308 observed at lower concentrations of A-443654. This result correlates well with the steady-state NanoBRET experiments, which show less A-443654 binding in the presence of BX-795.

Taken together, the *K*_i_ measurements from the NanoBRET steady-state experiments stem from an intrinsic inhibitor binding affinity modulated by AKT’s starting phosphorylation state population and the inhibitors’ induced phosphorylation changes. Nonetheless, our main conclusion is that the binding affinity of ATP-competitive inhibitors is stronger for AKT when phosphorylated at T308.

### T308 Phosphorylation Plays a Role in Triple-Negative Breast Cancer Sensitivity to ATP-Competitive AKT Inhibitors

Triple-negative breast cancer subtypes are genetically heterogeneous and have different sensitivities to AKT inhibitors.^31–33^ The MDA-MB-231 cell line is derived from a triple-negative breast cancer patient harboring mutations in the RAS-MAPK pathway in addition to alterations of P53. MDA-MB-468 is a cell line derived from a triple-negative breast cancer patient with deletion of PTEN that leads to overstimulation of the PI3K-AKT pathway. It has been previously reported that the MDA-MB-231 cell line is resistant to AKT inhibitors and the MDA-MB-468 is sensitive to AKT inhibitors.^34^ Based on data presented in Figures 4 and 5, we were curious to see if intrinsic differences in AKT phosphorylation between MDA-MB-231 and MDA-MB-468 cell lines would display differences in AKT inhibitor binding. We first tested the effects of ipatasertib, capivasertib, A-443654, MK-2206, miransertib, and inhibitor VIII on the cell viability of both cell lines. We found that the MDA-MB-468 cell line was more sensitive to ATP-competitive inhibitors and similarly as sensitive to allosteric inhibitors relative to the MDA-MB-231-cell line (Figures 6A,B and S8). The largest difference in inhibitor sensitivity between the two cell lines was observed for A-443654 (IC_50_: MDA-MB-231 0.33 ± 0.01 μM; MDA-MB-468 0.020 ± 0.001 μM; “±” indicates the standard error of the mean (*n* = three biological replicates)).

**Figure 6:**
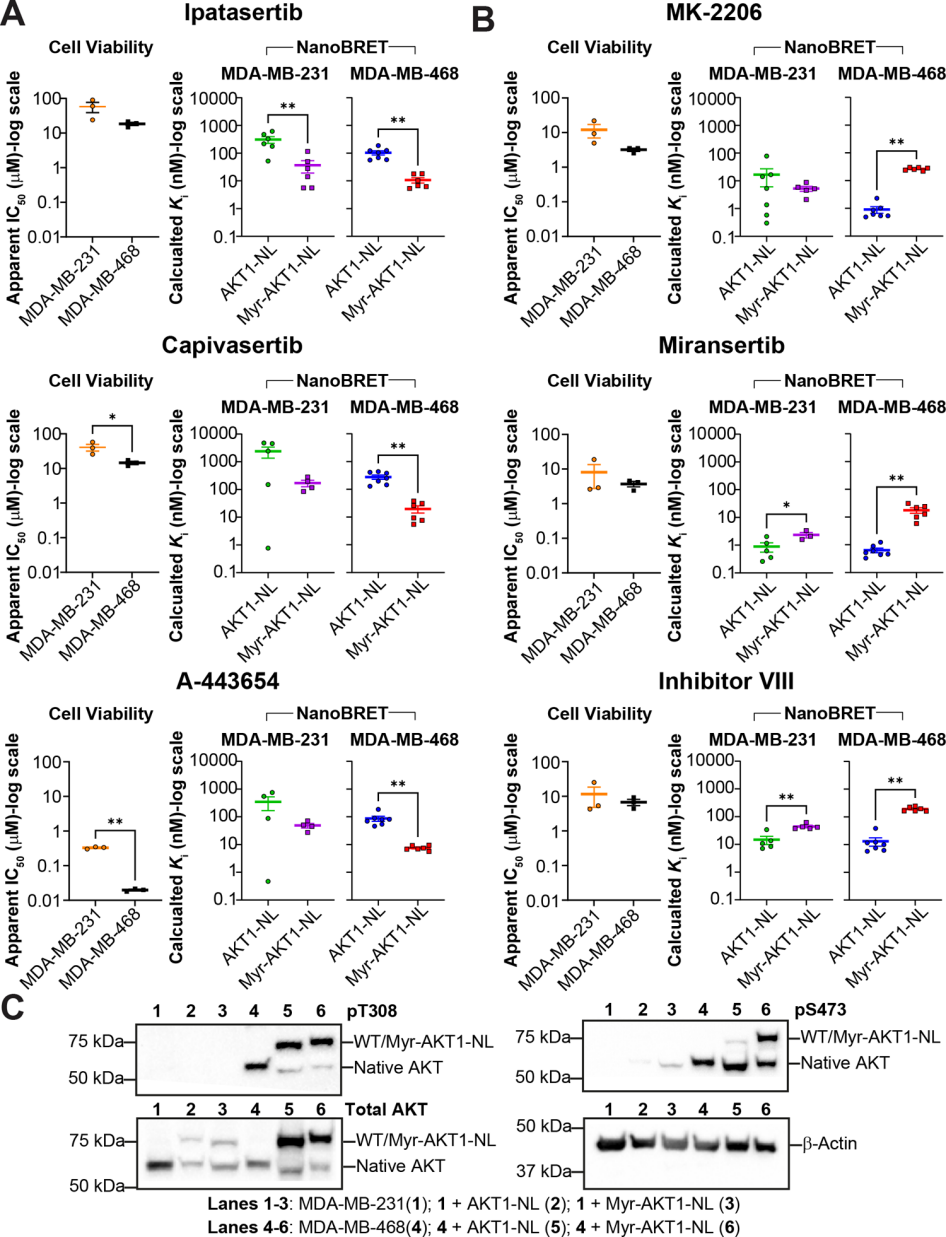
Correlation of cell viability and target engagement of AKT inhibitors on AKT inhibitor sensitive and resistant triple-negative breast cancer cell lines. (A) Cell viability comparison of MDA-MB-231 and MDA-MB-468 in the presence of ATP-competitive inhibitors. Capivasertib: MDA-MB-231 vs MDA-MB-468, *P* = 0.0483, *t* = 2.811, df = 4, two-tailed unpaired *t*-test. A-443654: MDA-MB-231 vs MDA-MB-468, *P* = 0.0021, *t* = 21.14, df = 2.023, two-tailed Welch’s *t*-test. NanoBRET target engagement assays of ATP-competitive inhibitors on MDA-MB-231 or MDA-MB-468 cell lines transfected with either AKT1-NL or Myr-AKT1-NL. Ipatasertib_231_: AKT1-NL vs Myr-AKT1-NL, *P* = 0.0043, *U* = 1, two-tailed Mann–Whitney *U* test. Ipatasertib_468_: AKT1-NL vs Myr-AKT1-NL, *P* = 0.0012, *U* = 0, two-tailed Mann–Whitney *U* test. Capivasertib_468_: AKT1-NL vs Myr-AKT1-NL, *P* = 0.0012, *U* = 0, two-tailed Mann–Whitney *U* test. A-443654_468_: AKT1-NL vs Myr-AKT1-NL, *P* = 0.0012, *U* = 0, two-tailed Mann–Whitney *U* test. (B) Cell viability comparison of MDA-MB-231 and MDA-MB-468 in the presence of allosteric inhibitors, and NanoBRET target engagement assays of allosteric inhibitors on MDA-MB-231 or MDA-MB-468 cell lines transfected with either AKT1-NL or Myr-AKT1-NL. Miransertib_231_: AKT1-NL vs Myr-AKT1-NL, *P* = 0.0714, *U* = 1, two-tailed Mann–Whitney *U* test. Inhibitor VIII_231_: AKT1-NL vs Myr-AKT1-NL, *P* = 0.0025, *t* = 4.347, df = 8, two-tailed unpaired *t*-test. MK-2206_468_: AKT1-NL vs Myr-AKT1-NL, *P* = 0.0012, *U* = 0, two-tailed Mann–Whitney *U* test. Miransertib_468_: AKT1-NL vs Myr-AKT1-NL, *P* = 0.0012, *U* = 0, two-tailed Mann–Whitney *U* test. Inhibitor VIII_468_: AKT1-NL vs Myr-AKT1-NL, *P* = 0.0012, *U* = 0, two-tailed Mann–Whitney *U* test. (C) Western blot comparing the phosphorylation state of T308 and S473 in MDA-MB-231 and MDA-MB-468 cell lines alone or transfected with either AKT1-NL or Myr-AKT1-NL. *P < 0.05; **P < 0.01; ****P < 0.0001. Each point for (A,B) represents a separate transfection (biological replicate). Each biological replicate has one technical replicate. Error bars represent the SEM of the biological replicates.

To understand the contribution of phosphorylation to these results, we performed a Western blot analysis on both cell lines separately transfected with AKT1-NL and Myr-AK1-NL to query the phosphorylation state of T308 and S473. We found that the transfected constructs were phosphorylated in the MDA-MB-468 cell line but not in the MDA-MB-231 cell line (Figures 6C and S9). In the MDA-MB-468 cell line, Myr-AKT1-NL showed 1.7× increased phosphorylation (see Methods) of T308 compared to AKT1-NL. The lack of phosphorylation in the MDA-MB-231 cell line is consistent with previous literature.^32^ To test if cell viability differences between cell lines correlated with differences in target engagement with different AKT conformations, we transfected AKT1-NL or Myr-AKT1-NL into both cell lines. We performed steady-state NanoBRET competition assays for the same AKT inhibitors. Interestingly for MDA-MB-231 cells, we observed similar binding between AKT1-NL and Myr-AKT1-NL for almost all inhibitors (Figures 6A,B and S10). In agreement with experiments performed using HEK293T cells, we observed an increase in ATP-competitive inhibitor binding affinity and a decrease in allosteric inhibitor binding for MDA-MB-468 cells (Figures 6A,B and S10). It is important to note that although there were larger differences in the binding of allosteric inhibitors to AKT1-NL and Myr-AKT1-NL in MDA-MB-468 vs MDA-MB-231 cell lines, these differences did not translate to effects on cell viability. These results highlight the utility of using T308 phosphorylation to predict the effects of ATP-competitive inhibitors on cell viability. These results also indicate that the steady-state NanoBRET assay can be used to report on differences in genetic backgrounds between cell lines.

## Conclusions

In conclusion, we demonstrated a NanoBRET target engagement assay to query the binding of ATP-competitive and allosteric AKT inhibitors to all AKT isoforms. We have shown that ATP-competitive inhibitors bind similarly to all isoforms and that allosteric inhibitors are generally selective for AKT1 and AKT2 over AKT3. Using a combination of pathogenic and nonphosphorylatable mutants, and an upstream PDK1 inhibitor, we found that phosphorylation of AKT1 at T308 enhances the binding of ATP-competitive inhibitors. These results are the first to measure the direct binding of AKT inhibitors to AKT in a cellular context and represent a platform for screening of new inhibitors. We also highlight that AKT gain-of-function mutations bind better to ATP-competitive inhibitors and that T308 phosphorylation is an important consideration in developing inhibitors that target specific states of AKT and as a marker to indicate the potential effectiveness of ATP-competitive inhibitors.

## Methods

### Materials

Myristoylated constructs of human AKT1 (UniProt: P31749), and AKT2 (UniProt: P31751), and AKT3 (UniProt: Q9Y243) were purchased from Addgene (cat. nos. 9008, 9016, and 9017, respectively). pNLF1-N (cat. no. N1351) and pNLF1-C (cat. no. N1361) vectors, K5 10,000× NanoBRET kits (cat. no. N2530), K16 1000× NanoBRET Kit (cat. no. CS1810C495), PDK1-NL vector (cat. no. CS1810C771), and GloMax Discover (cat. no. GM3000) were purchased from Promega. QuikChange mutagenesis kits were purchased from Agilent (cat# 200522). Ipatasertib (cat. no. HY-15186), capivasertib (cat. no. HY-15431), MK-2206 (cat. no. HY-10358), miransertib (cat. no. HY-19719), and inhibitor VIII (cat. no. HY-10355) were purchased from MedChem Express. A-443654 was purchased from Ontario Chemicals (cat# I1939). Resazurin was purchased from Fisher Scientific (cat# AC418900050). White 96-well plates for NanoBRET experiments (cat. no. 07200628) and clear plastic 96-well plates (cat. no. 229195) for drug dilutions and cell viability experiments were purchased from Fisher Scientific. For cell culture, Opti-MEM (cat. no. 11-058-021), DMEM (cat. no. 11-965-092), FBS (cat. no. 26140079), trypsin (cat. no. 25200056), penicillin/streptomycin (cat. no. 15140122), and PBS (cat. no. 20-012-027) were purchased from Thermo Fisher Scientific. For transfections, PEI Star (cat. no. 78-541-00) was purchased from Fisher Scientific, and Fugene HD was purchased from Promega (cat# E2311). For Western blots, BSA (catalog no. A9647), Thermo Scientific Halt Protease and Phosphatase Inhibitor Cocktail (100×) (catalog no. PI78440), RIPA buffer (catalog no. 89900), and SuperSignal West Femto Substrate Kit (catalog no. 34096) were purchased from Fisher Scientific. Pierce BCA Protein Assay Kits (catalog no. A55864) and Trans-Blot Turbo RTA Transfer Kits (catalog no. 1704273) were purchased from Bio-Rad. Antibodies Phospho-AKT­(Thr308) (D25E6) XP Rabbit mAb (cat. no. 13038), Phospho-AKT (Ser473) (D9E) XP Rabbit mAb (cat. no. 4060), AKT (pan) (C67E7) Rabbit mAb (cat. no. 4691), β-Actin (D6A8) Rabbit mAb (cat. no. 8457), and Antirabbit IgG-HRP-linked Antibody (cat. no. 7074) were purchased from Cell Signaling. The HEK293T cell line was acquired from ATCC (cat. no. CRL-3216), and the MDA-MB-231 and MDA-MB-468 cell lines were generously provided by Dr. Natalie R. Gassman.

### Constructs and Mutagenesis

To generate N- or C-terminal NanoLuciferase fusion constructs, full-length versions of AKT1, AKT2, and AKT3, were separately subcloned into pNLF1-N or pNLF1-C vectors between PvuI and XbaI, and between PvuI and XhoI, respectively. Myristoylated versions of AKT1, AKT2, and AKT3 were subcloned into the pNLF1-C vector. PDK1-NL was purchased from Promega. All pathological and myristoylated mutants were made by using the QuikChange mutagenesis kit.

### Steady-State NanoBRET Competition Assays

HEK293T, MDA-MB-231, or MDA-MB-468 cells were transfected at a cell density of 200,000 cells/mL in Opti-MEM (containing 10% FBS and 1000 U/mL Pen/Strep) using either PEI Star or Fugene HD, and a ratio of 9.5:0.5 μg/mL carrier DNA/AKT-NL or carrier DNA/PDK1-NL constructs in Opti-MEM. For coexpression experiments with Myr-AKT1-NL and PDK1, a DNA ratio of 9:0.5:0.5, μg/mL carrier DNA/Myr-AKT1-NL: PDK1 was used. This solution was aliquoted into white adherent 96-well plates at a volume of 100 μL/well and incubated for 24 h at 37 °C and 5% CO_2_. For steady-state apparent IC_50_ measurements, 10 μL of a 10× AKT inhibitor/BX-795 dose–response curve (100, 10, 1, 0.1, 0.01, 0.001, 0.0001, 0.00001 μM final concentration at 0.1% DMSO) made in Opti-MEM, followed by 5 μL of a 20× K5 tracer solution (made according to Promega’s adherent target engagement protocol). For steady-state K5 EC_50_ measurements, 5 μL of a 20× K5 tracer dose–response curve (4, 2, 1, 0.5, 0.1, 0.05, 0.01, 0.005 μM final concentration) was added to transfected cells. For steady-state K5 EC_50_ measurements in the presence of 1 μM final BX-795 for AKT1-NL, Myr-AKT1-NL. Myr-AKT1-NL + PDK1 (0.5:0.5 μg/mL), Myr-AKT1-NL + PDK1 (0.5:1 μg/mL), or Myr-AKT1-NL + PDK1 (0.5:2 μg/mL), 5 μL of 20× K5 tracer dose–response curve (4, 2, 1, 0.5, 0.1, 0.05, 0.01, and 0.005 μM final concentration) was added to transfected cells, in addition to 10 μL of 10× BX-795 in Opti-MEM, or DMSO in Opti-MEM (0.1% final). For steady-state IC_50_ measurements of AKT inhibitors in the presence of BX-795, 10 μL of a 10× dose–response for each AKT inhibitor was added to each well, followed by 10 μL of BX-795 (1 μM final) and 5 μL of a 20× K5 tracer solution (0.2% DMSO final). Following all inhibitor and tracer additions, cells were incubated at 37 °C and 5% CO_2_ for 2 h. 50 μL of a 3× solution of Opti-MEM containing a NanoGlo substrate and NanoLuciferase inhibitor provided in the NanoBRET kit were added to each well. BRET data were collected on a GloMax Discover using the preset NanoBRET 618 protocol, exported in Microsoft Excel format, and analyzed in GraphPad Prism 10. Statistics were performed in Origin 2023.

Calculated *K*_i_ values were calculated using the following formula

$$K_{i}=\frac{IC_{50,tracer}}{\left(1+\frac{\left[tracer\right]}{EC_{50,tracer}}\right)}$$

### Western Blot Analysis of AKT Phosphorylation

1.1 million cells in 5.28 mL of Opti-MEM were seeded into 35 mm tissue culture-treated dishes transfected using either polyethylenimine or Fugene HD, and a ratio of 9:1 μg/mL carrier DNA/AKT-NL construct. For drug-incubation experiments, 100 μL of A-443654 dose–response curve (100, 10, 1, 0.1, 0.01, 0.001, 0.0001, 0.00001 μM final concentration at 0.1% DMSO) and 528 μL of 1 μM BX-795 or 528 μL of 1 μM DMSO made in Opti-MEM, followed by 256 μL of a 20× K5 tracer solution (made according to Promega’s adherent target engagement protocol) were added to plates and incubated at 37 °C for 24 h. Cells were harvested using a solution of phosphatase and protease inhibitors (1:100 dilution) in RIPA buffer. Protein quantification was performed using the Pierce BCA Protein Assay Kit protocol. Data were quantified using the preset BCA 560 nm absorbance protocol on the GloMax Discover plate reader, exported in Microsoft Excel format, and analyzed in GraphPad Prism 10. SDS-Page gel electrophoresis was performed with 10 μg of protein per well. Proteins were transferred to PVDF membranes utilizing a Trans-Blot Turbo RTA Transfer Kit and Trans-Blot Turbo Transfer System. Membranes were blocked in 5% milk in TBST at RT for 1 h before overnight incubation in a primary antibody solution at 4 °C on a rocking shaker. Dilutions for T308, β-Actin, and Total AKT primary antibodies were 1:1000 in 5% milk in TBST, and S473 was 1:2000 dilution in 5% milk in TBST. The secondary antibody was made at a 1:20,000 dilution in 2.5% BSA in TBST and was added after washing membranes 3× in TBST. Gels were imaged using SuperSignal West Femto Substrate Kit and ChemiDoc MP Imager.

### Calculation of Fold Phosphorylation Change

Densitometry analysis for the MDA-MB-468 cell line in Figure 6C was performed in the Bio-Rad Image Lab 6. Raw intensities were used in the following formula

$$pT308\;band\;intensity\;fold\;change_{Myr‐AKT1‐NL/AKT1‐NL}=\frac{\frac{I_{pT308,\;Myr‐AKT1‐NL}}{I_{\beta ‐Actinp,\;T308,\;Myr‐AKT1‐NL}}}{\frac{I_{total\;AKT,\;Myr‐AKT1‐NL}}{I_{\beta ‐actin,\;total\;AKT,\;Myr‐AKT1‐NL}}}\times \frac{\frac{I_{total\;AKT,\;AKT1‐NL}}{I_{\beta ‐actin,\;total\;AKT,\;AKT1‐NL}}}{\frac{I_{pT308,\;AKT1‐NL}}{I_{\beta ‐actin,\;pT308,\;AKT1‐NL}}}$$

Band intensities for Myr-AKT1-NL and AKT1-NL (pT308 and total AKT) were normalized to their corresponding β-Actin band intensities and then were divided.

### MDA-MB-231 and MDA-MB-468 Cancer Cell Lines’ Viability Assay

Cultured MDA-MB-468 or MDA-MB-231 cells were trypsinized and transferred to clear adherent 96-well plates (5000 cells/well, 90 μL/well) and incubated overnight in DMEM. AKT inhibitors ipatasertib, capivasertib, MK-2206, A443654, miransertib, and inhibitor VII were then added separately at final concentrations of 100, 10, 1, 0.1, 0.01, 0.001, and 0.0001 μM (0.1% DMSO final using 10 μL/well) and incubated for 5 days at 37 °C with 5% CO_2_. Cell viability was assessed using a resazurin assay where 10 μL of resazurin (0.01 mg mL^–1^ final, 0.1 mg mL^–1^ stock made in 1× PBS, pH 7.2) was added to each well and incubated at 37 °C with 5% CO_2_ for 2 h. 96-well plates were then read on a GloMax Discover plate reader using the CellTiter-Blue Cell Viability Assay Protocol. For each condition, a control with DMSO (no inhibitor) was included, and viability was determined by dividing the intensity of each condition by the control. Data were exported and analyzed in Microsoft Excel and GraphPad Prism 10. Statistics were performed in Origin 2023.

### Bioluminescent Imaging

HEK293T cells were transfected at a cell density of 200,000 cells/mL in DMEM (containing 10% FBS and 1000 U/mL Pen/Strep) using Fugene HD and a ratio of 9.5:0.5 μg/mL carrier DNA/AKT-NL construct in Opti-MEM. This solution was aliquoted into 8-well glass bottom μ-slide plates at a volume of 400 μL/well and incubated for 24 h at 37 °C and 5% CO_2_. After incubation, 2 μL of NanoGlo substrate was added to each well and imaged using the GloMax Galaxy Bioluminescence Imager utilizing a 40× objective (Nikon CFI Plan Fluor- Material Number MRH01401) and exposure times ranging from 32 to 100 s. For experiments assessing localization changes induced by AKT inhibitors, following transfection and overnight incubation, 40 μL of a 10× solution of each drug in OptiMEM was added to each well and incubated at 37 °C and 5% CO_2_ for 2 h before imaging. Images were processed in Fiji.

## Supplementary Material



## Notes

Assay optimization for NanoLuciferase placement and AKT inhibitor binding curve replicate data; replicate and additional bioluminescent imaging data and uncropped western blots associated with some of the figures in the paper; and replicate cell viability curves and *K*_i_, fitted tracer EC_50_, and fitted IC_50_ values for MDA-MB-231 and MDA-MB-468 cell lines (PDF)
