# Supplementary material for: Live-Cell NanoBRET Assay to Measure AKT Inhibitor Binding to Conformational States of AKT
Source: ACS Chem Biol. 2025 Jul 9;20(7):1635–45. doi: 10.1021/acschembio.5c00213 (PMC12281477; doi:10.1021/acschembio.5c00213)
Supplement: Supplementary file 1 [file cb5c00213_si_002.pdf]

Supporting Information

Live-cell NanoBRET assay to measure AKT inhibitor binding to conformational states of AKT

Jeremy W. Harris, Flávio Antônio de Oliveira Simões, Erin N. Ryerson, and William M. Marsiglia

Table of Contents

Figure	Page
Figure S1	2-6
Figure S2	7-9
Figure S3	10-12
Figure S4	13-23
Figure S5	24
Figure S6	25-26
Figure S7	27-30
Figure S8	31-32
Figure S9	33
Figure S10	34-39

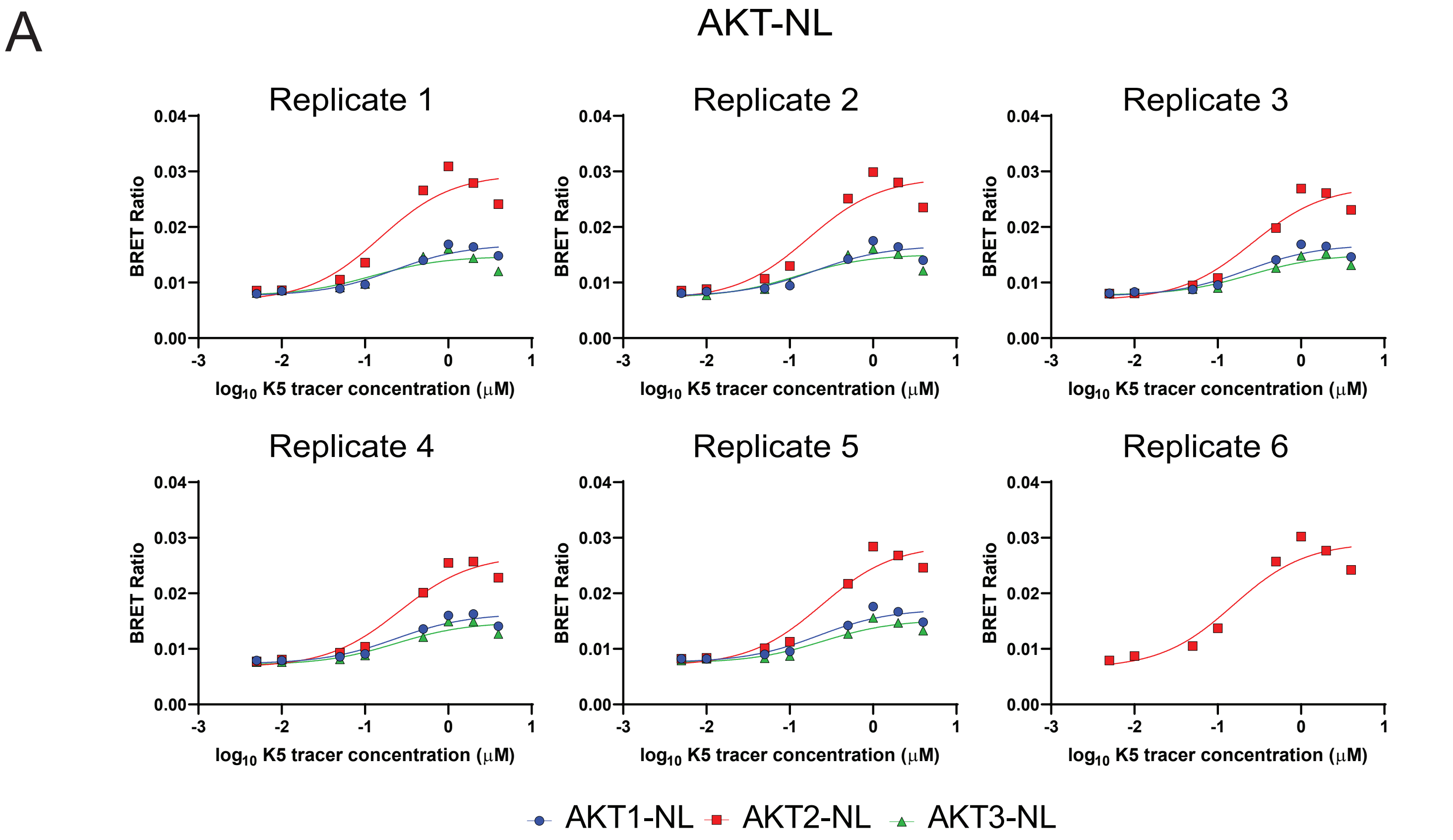

K5 EC_{50}	Replicate 1 (μM)	Replicate 2 (μM)	Replicate 3 (μM)	Replicate 4 (μM)	Replicate 5 (μM)	Replicate 6 (μM)	Average (μM)	Standard Error
AKT1-NL	0.2232	0.1977	0.2204	0.2331	0.2179		0.2185	0.0058
AKT2-NL	0.1525	0.1666	0.2598	0.2591	0.2395	0.1527	0.2050	0.0217
AKT3-NL	0.1219	0.1215	0.2444	0.2320	0.2460		0.1932	0.0293

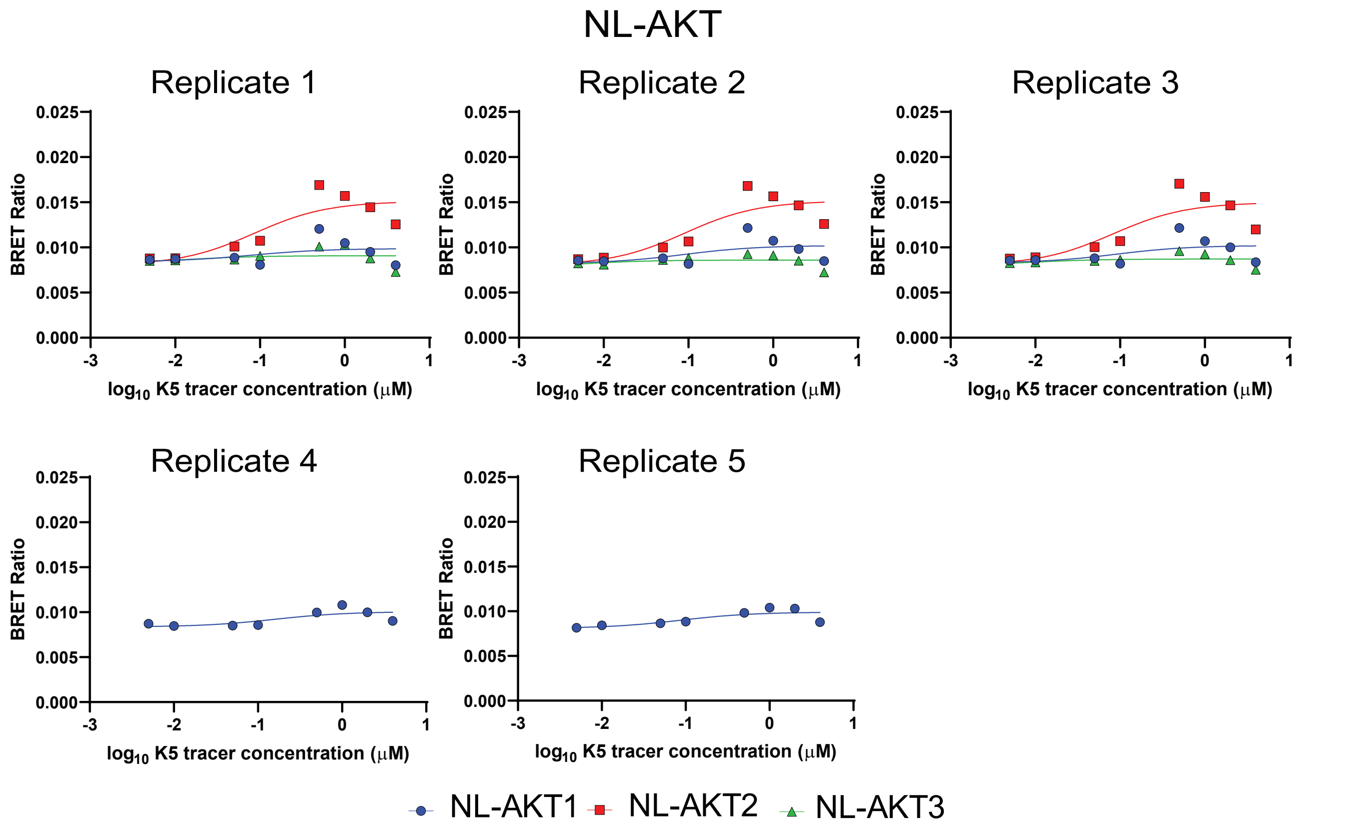

K5 EC_{50}	Replicate 1 (μM)	Replicate 2 (μM)	Replicate 3 (μM)	Replicate 4 (μM)	Replicate 5 (μM)	Average (μM)	Standard Error
NL-AKT1	0.0819	0.0883	0.0890	0.1580	0.0867	0.1008	0.0144
NL-AKT2	0.0853	0.0903	0.0830			0.0862	0.0022
NL-AKT3	0.0136	0.0042	0.0079			0.0085	0.0027

B

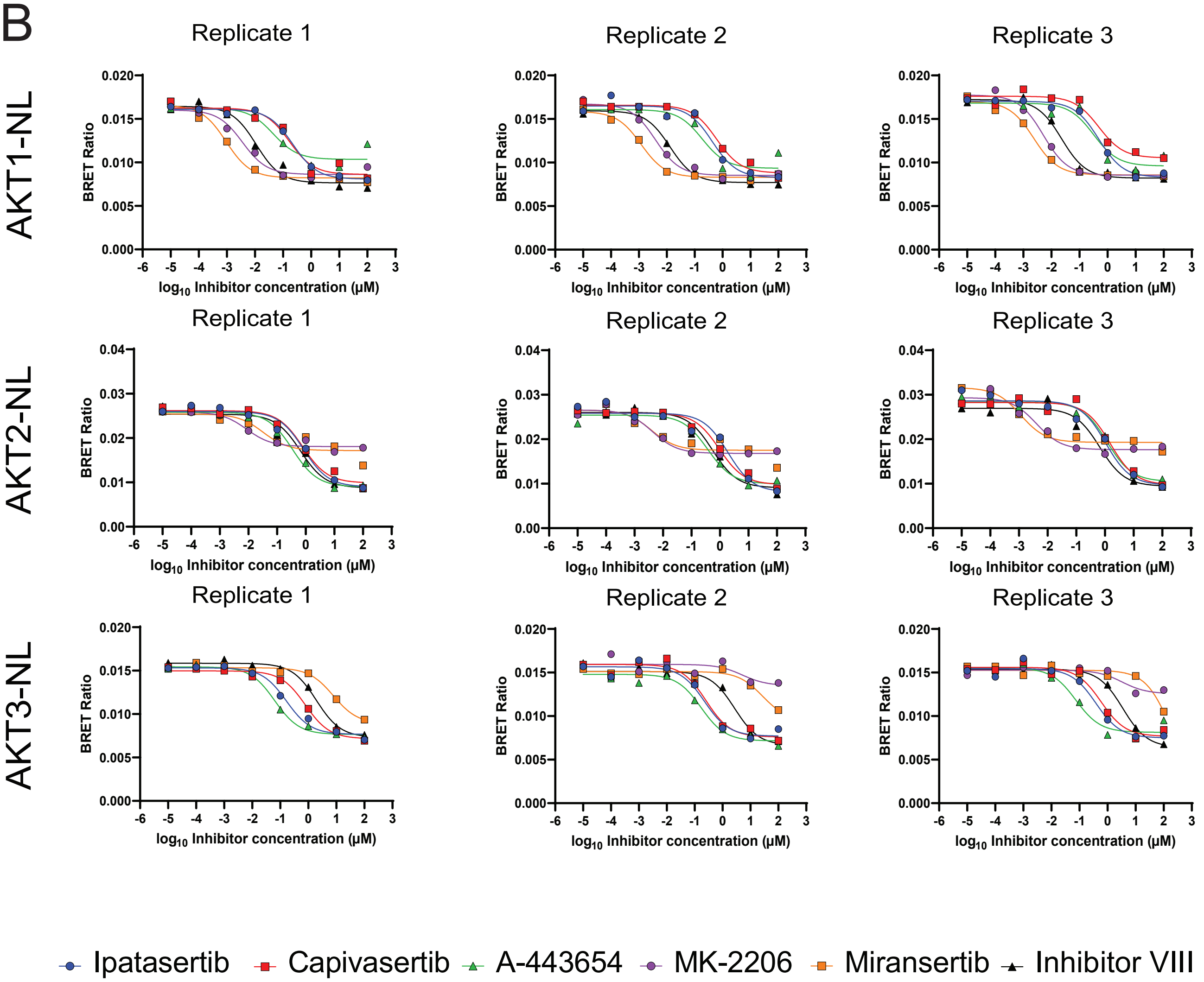

C

Apparent IC_{50} values

AKT1-NL IC_{50}	Replicate 1 (μM)	Replicate 2 (μM)	Replicate 3 (μM)	Average (μM)	Standard Error
Ipatasertib	0.2220	0.4338	0.4931	0.3830	0.0823
Capivasertib	0.1661	0.6136	0.4913	0.4237	0.1335
A-443654	0.0445	0.1863	0.3170	0.1826	0.0787
MK-2206	0.0036	0.0039	0.0052	0.0042	0.0005
Miransertib	0.0009	0.0012	0.0023	0.0015	0.0004
Inhibitor VIII	0.0112	0.0129	0.0232	0.0158	0.0038

AKT2-NL IC_{50}	Replicate 1 (μM)	Replicate 2 (μM)	Replicate 3 (μM)	Average (μM)	Standard Error
Ipatasertib	0.8388	1.9130	1.0600	1.2706	0.3275
Capivasertib	0.7908	0.9428	1.5450	1.0929	0.2303
A-443654	0.3491	0.3740	1.1840	0.6357	0.2742
MK-2206	0.0087	0.0043	0.0041	0.0057	0.0015
Miransertib	0.0325	0.0039	0.0011	0.0125	0.0100
Inhibitor VIII	0.6635	0.5476	0.6634	0.6248	0.0386

AKT3-NL IC_{50}	Replicate 1 (μM)	Replicate 2 (μM)	Replicate 3 (μM)	Average (μM)	Standard Error
Ipatasertib	0.1660	0.2252	0.3850	0.2587	0.0654
Capivasertib	0.7786	0.2567	0.5909	0.5421	0.1526
A-443654	0.0634	0.1698	0.0746	0.1026	0.0338
MK-2206	N/A	N/A	N/A	N/A	N/A
Miransertib	7.81	26.78	154.90	63.16	46.19
Inhibitor VIII	1.94	2.71	3.48	2.71	0.45

D

Calculated K_i values

AKT1-NL K_i	Replicate 1 (nM)	Replicate 2 (nM)	Replicate 3 (nM)	Average (nM)	Standard Error
Ipatasertib	39.80	77.77	88.41	68.66	14.75
Capivasertib	29.78	110.01	88.09	75.96	23.94
A-443654	7.98	33.40	56.84	32.74	14.11
MK-2206	0.6433	0.6991	0.9264	0.7563	0.0866
Miransertib	0.1685	0.2152	0.4195	0.2677	0.0771
Inhibitor VIII	2.00	2.32	4.16	2.83	0.67

AKT2-NL K_i	Replicate 1 (nM)	Replicate 2 (nM)	Replicate 3 (nM)	Average (nM)	Standard Error
Ipatasertib	142.72	325.49	180.36	216.19	55.72
Capivasertib	134.55	160.42	262.88	185.95	39.18
A-443654	59.40	63.64	201.45	108.16	46.66
MK-2206	1.47	0.73	0.69	0.97	0.25
Miransertib	5.53	0.66	0.20	2.13	1.71
Inhibitor VIII	112.89	93.17	112.88	106.31	6.57

AKT3-NL K_i	Replicate 1 (nM)	Replicate 2 (nM)	Replicate 3 (nM)	Average (nM)	Standard Error
Ipatasertib	26.87	36.46	62.33	41.89	10.59
Capivasertib	126.05	41.56	95.66	87.75	24.71
A-443654	10.26	27.49	12.08	16.61	5.46
MK-2206	N/A	N/A	N/A	N/A	N/A
Miransertib	1264.36	4335.40	25076.67	10225.48	7478.33
Inhibitor VIII	313.42	438.72	564.02	438.72	72.34

E

AKT1 bound to AMP-PNP (PDB: 4EKK)

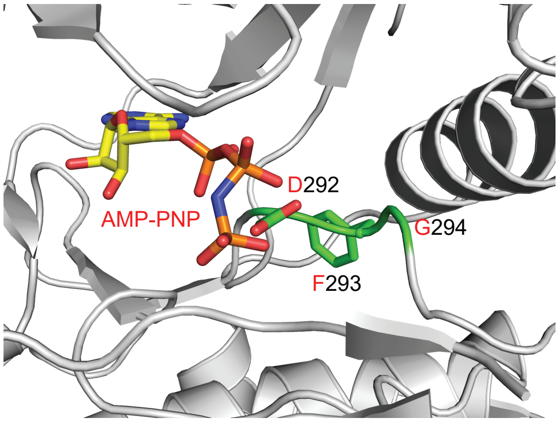

AKT1 bound to Inhibitor VIII (allosteric; PDB: 3O96)

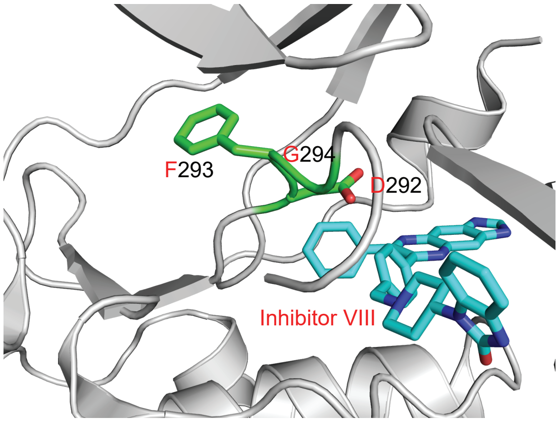

Hypothetical structure of AKT1 bound to Inhibitor VIII (allosteric) and AMP-PNP. Steric clash between F293 and AMP-PNP

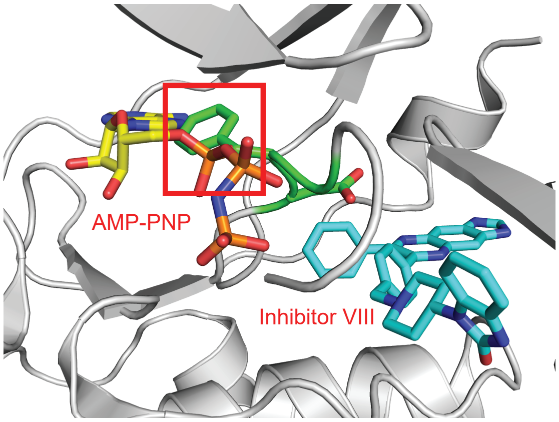

Hypothetical structure of AKT1 bound to Inhibitor VIII (allosteric) and A-443654 (ATP-competitive: PDB 2JDR). Steric clash between F293 and A-443654

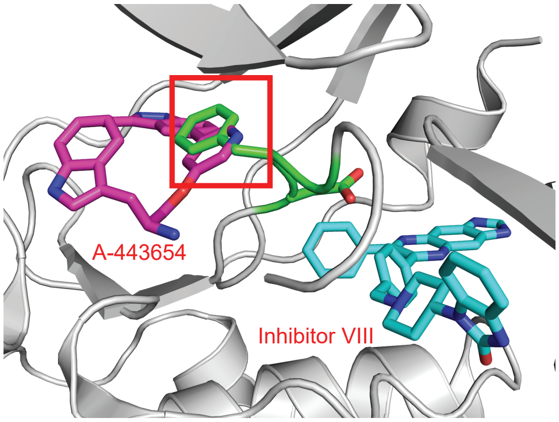

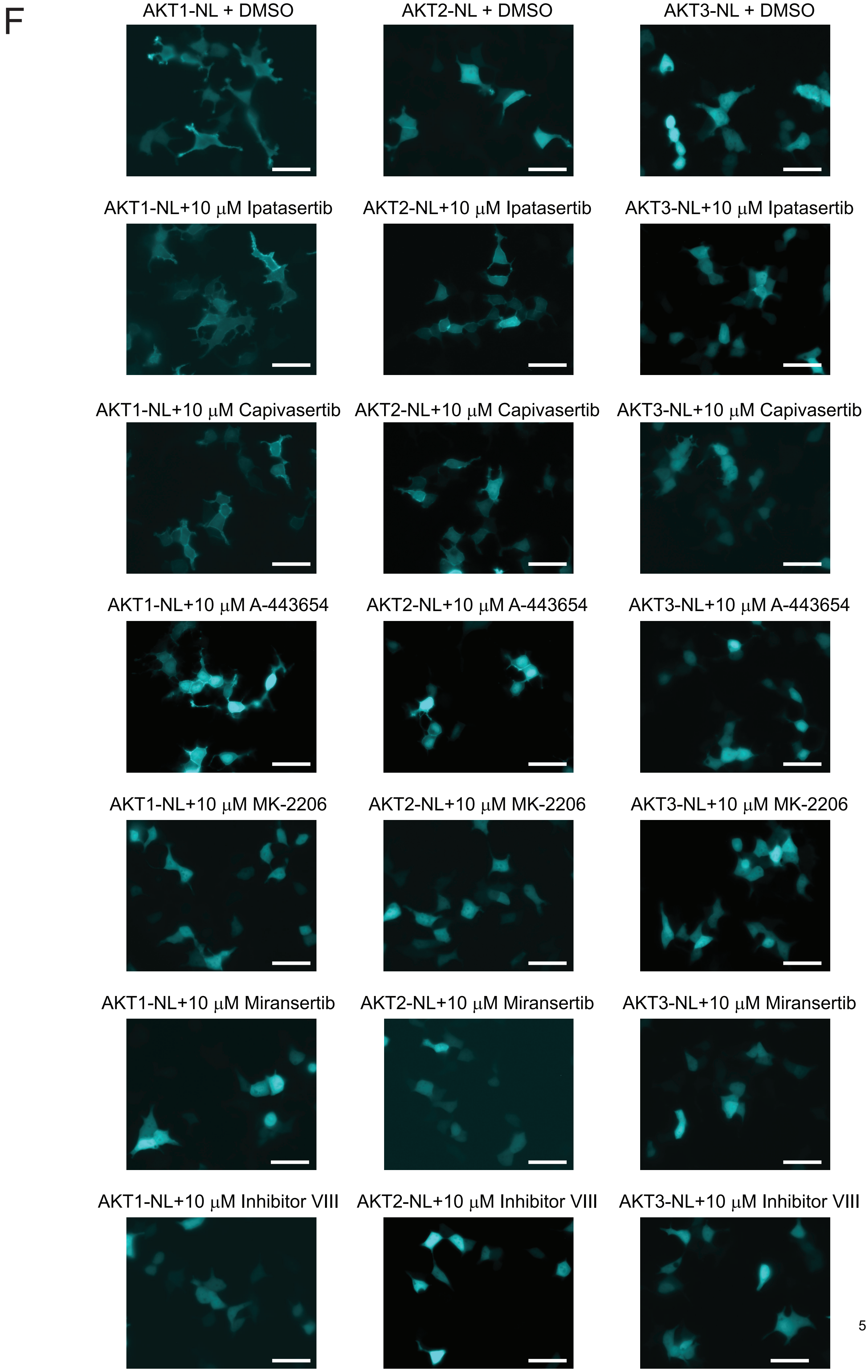
F
AKT1-NL + DMSO
AKT2-NL + DMSO
AKT3-NL + DMSO
AKT1-NL+10 μM Ipatasertib
AKT2-NL+10 μM Ipatasertib
AKT3-NL+10 μM Ipatasertib
AKT1-NL+10 μM Capivasertib
AKT2-NL+10 μM Capivasertib
AKT3-NL+10 μM Capivasertib
AKT1-NL+10 μM A-443654
AKT2-NL+10 μM A-443654
AKT3-NL+10 μM A-443654
AKT1-NL+10 μM MK-2206
AKT2-NL+10 μM MK-2206
AKT3-NL+10 μM MK-2206
AKT1-NL+10 μM Miransertib
AKT2-NL+10 μM Miransertib
AKT3-NL+10 μM Miransertib
AKT1-NL+10 μM Inhibitor VIII
AKT2-NL+10 μM Inhibitor VIII
AKT3-NL+10 μM Inhibitor VIII

Figure S1- Assay optimization for NanoLuciferase placement and AKT inhibitor binding curve replicate data. **(A)** Signal build-up curves and fitted EC_{50} values using the K5 tracer on 293T cells transfected with AKT-NL or NL-AKT. **(B)** Binding curves for AKT inhibitors on AKT1-NL, AKT2-NL, and AKT3-NL. **(C)** Fitted apparent IC_{50} values for curves in (B). **(D)** Calculated K_i values derived from EC_{50} and IC_{50} values in (A) and (C). **(E)** Structural rationale for how allosteric inhibitor binding is not compatible with ATP/ATP-competitive inhibitor binding. **(F)** Bioluminescent Imaging of HEK293T cells transfected with AKT1-NL, AKT2-NL, or AKT3-NL, and treated with AKT inhibitors for 2 hrs at 37 ^{o}C and 5 % CO_2. Scale bar indicates 50 μm.

A

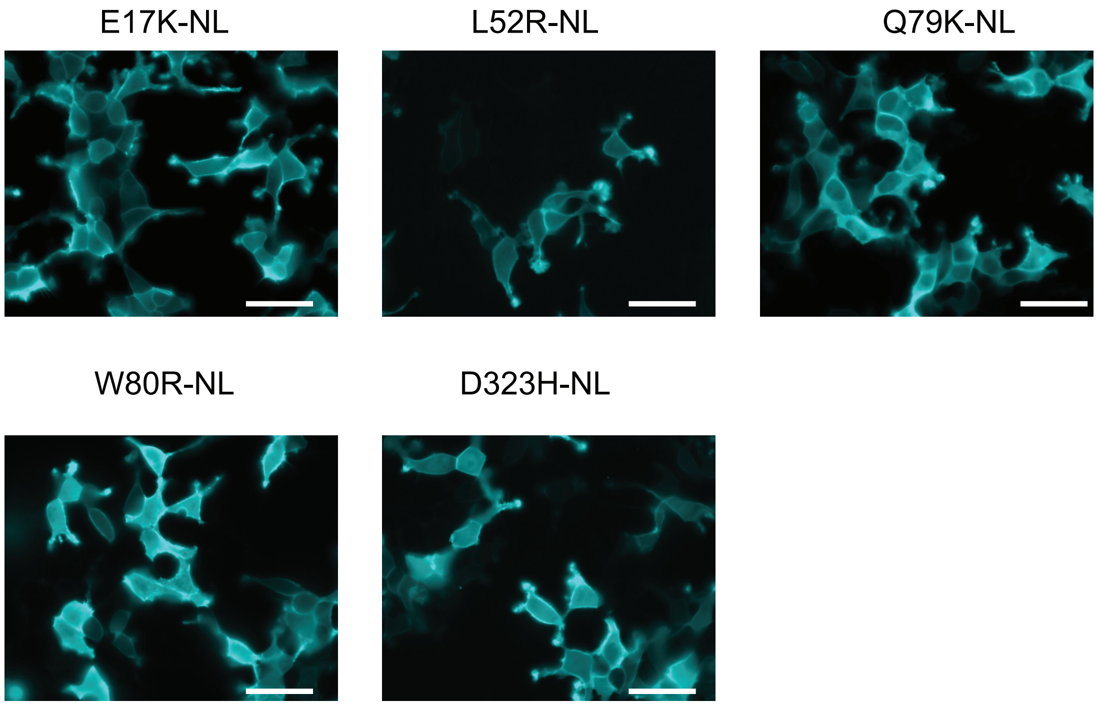

B

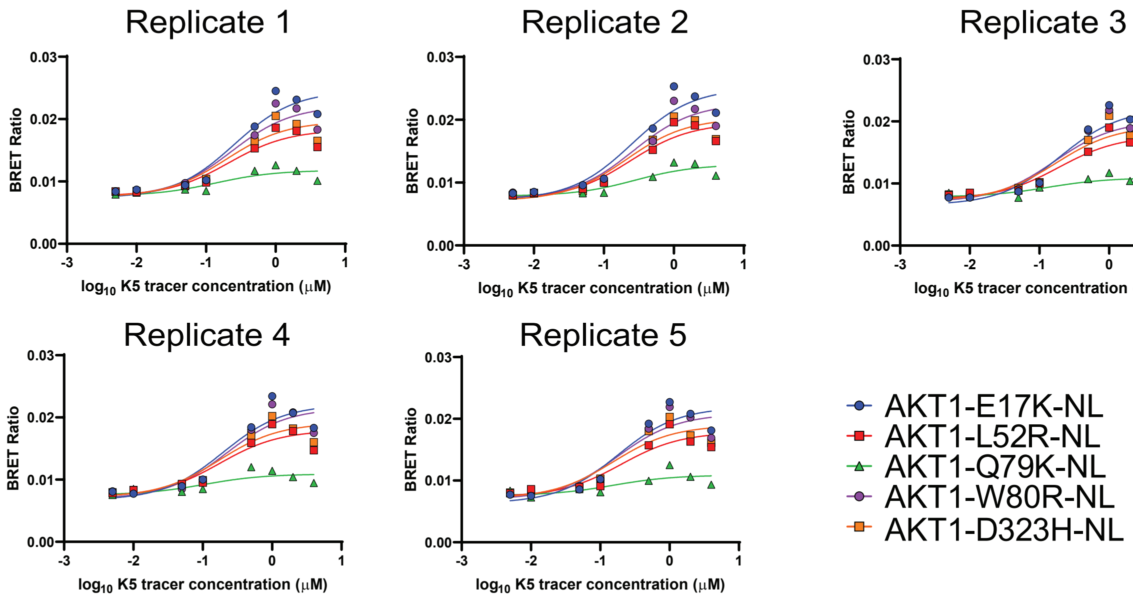

K5 EC_{50}	Replicate 1 (μM)	Replicate 2 (μM)	Replicate 3 (μM)	Replicate 4 (μM)	Replicate 5 (μM)	Average (μM)	Standard Error
AKT1-E17K-NL	0.2542	0.2503	0.1959	0.1958	0.1777	0.2148	0.0157
AKT1-L52R-NL	0.2103	0.2384	0.1890	0.1741	0.1983	0.2020	0.0109
AKT1-Q79K-NL	0.1340	0.2766	0.1313	0.1043	0.1534	0.1599	0.0302
AKT1-W80R-NL	0.2318	0.2607	0.1640	0.2160	0.1763	0.2098	0.0178
AKT1-D323H-NL	0.1965	0.2087	0.1723	0.1724	0.1532	0.1806	0.0098

C

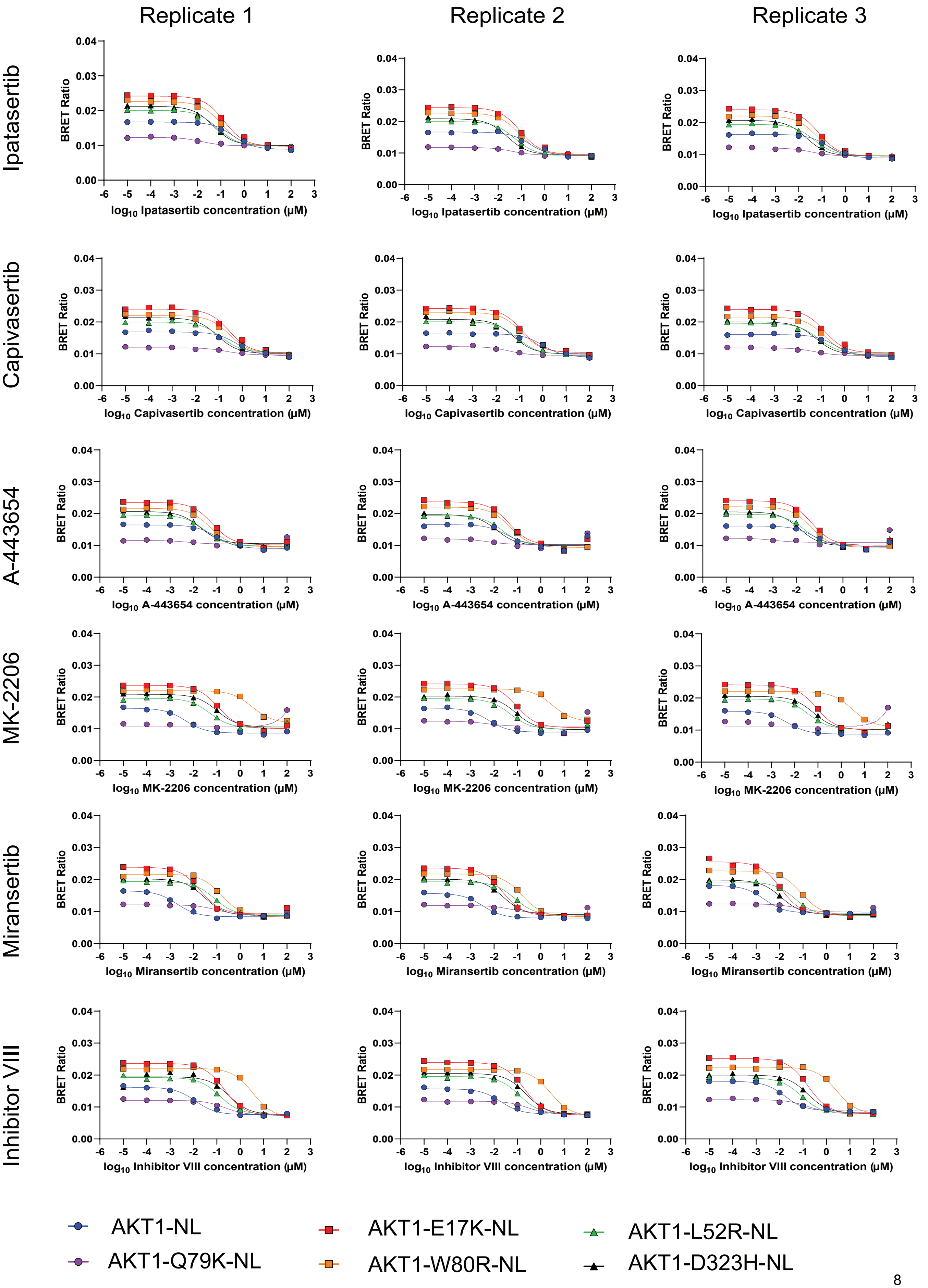
Replicate 1
Replicate 2
Replicate 3
Ipatasertib
Capivasertib
A-443654
MK-2206
Miransertib
Inhibitor VIII
BRET Ratio
log10 Ipatasertib concentration (µM)
log10 Capivasertib concentration (µM)
log10 A-443654 concentration (µM)
log10 MK-2206 concentration (µM)
log10 Miransertib concentration (µM)
log10 Inhibitor VIII concentration (µM)
AKT1-NL
AKT1-Q79K-NL
AKT1-E17K-NL
AKT1-W80R-NL
AKT1-L52R-NL
AKT1-D323H-NL

D

Apparent IC_{50} values

	Replicate 1 (µM)	Replicate 2 (µM)	Replicate 3 (µM)	Average (µM)	Standard Error
WT-AKT1-NL	0.3331	0.2278	0.1880	0.2496	0.0433
AKT1-E17K-NL	0.1384	0.0883	0.0724	0.0997	0.0199
AKT1-L52R-NL	0.0719	0.0515	0.0466	0.0567	0.0077
AKT1-Q79K-NL	0.0155	0.0402	0.0356	0.0304	0.0076
AKT1-W80R-NL	0.1060	0.0829	0.0586	0.0825	0.0137
AKT1-D323H-NL	0.0474	0.0252	0.0222	0.0316	0.0079

	Replicate 1 (µM)	Replicate 2 (µM)	Replicate 3 (µM)	Average (µM)	Standard Error
WT-AKT1-NL	0.5989	1.0380	0.4196	0.6855	0.1837
AKT1-E17K-NL	0.2811	0.1305	0.1409	0.1842	0.0486
AKT1-L52R-NL	0.1251	0.0820	0.0740	0.0937	0.0159
AKT1-Q79K-NL	0.2128	0.0359	0.0529	0.1005	0.0563
AKT1-W80R-NL	0.2812	0.1243	0.1444	0.1833	0.0493
AKT1-D323H-NL	0.0867	0.0569	0.0589	0.0675	0.0096

	Replicate 1 (µM)	Replicate 2 (µM)	Replicate 3 (µM)	Average (µM)	Standard Error
WT-AKT1-NL	0.0965	0.0349	0.0580	0.0631	0.0180
AKT1-E17K-NL	0.0637	0.0447	0.0462	0.0515	0.0061
AKT1-L52R-NL	0.0321	0.0226	0.0224	0.0257	0.0032
AKT1-Q79K-NL	0.0058	0.0052	0.0011	0.0041	0.0015
AKT1-W80R-NL	0.0531	0.0502	0.0424	0.0486	0.0032
AKT1-D323H-NL	0.0199	0.0130	0.0143	0.0157	0.0021

	Replicate 1 (µM)	Replicate 2 (µM)	Replicate 3 (µM)	Average (µM)	Standard Error
WT-AKT1-NL	0.0046	0.0057	0.0049	0.0050	0.0003
AKT1-E17K-NL	0.1125	0.0896	0.0821	0.0947	0.0091
AKT1-L52R-NL	0.0502	0.0489	0.0397	0.0463	0.0033
AKT1-Q79K-NL	N/A	0.0048	N/A	0.0048	N/A
AKT1-W80R-NL	3.2430	3.2500	2.9920	3.1617	0.0849
AKT1-D323H-NL	0.1013	0.0863	0.0656	0.0844	0.0103

	Replicate 1 (µM)	Replicate 2 (µM)	Replicate 3 (µM)	Average (µM)	Standard Error
WT-AKT1-NL	0.0019	0.0032	0.0023	0.0025	0.0004
AKT1-E17K-NL	0.0199	0.0236	0.0113	0.0183	0.0036
AKT1-L52R-NL	0.0798	0.0776	0.0461	0.0678	0.0109
AKT1-Q79K-NL	0.1000	0.0534	0.0580	0.0705	0.0148
AKT1-W80R-NL	0.1638	0.1601	0.0852	0.1364	0.0256
AKT1-D323H-NL	0.0292	0.0244	0.0117	0.0218	0.0052

	Replicate 1 (µM)	Replicate 2 (µM)	Replicate 3 (µM)	Average (µM)	Standard Error
WT-AKT1-NL	0.0122	0.0189	0.0169	0.0160	0.0020
AKT1-E17K-NL	0.2102	0.1978	0.1647	0.1909	0.0136
AKT1-L52R-NL	0.1332	0.1232	0.0849	0.1138	0.0147
AKT1-Q79K-NL	0.2213	0.2867	0.1492	0.2191	0.0397
AKT1-W80R-NL	3.4990	2.6190	2.7090	2.9423	0.2795
AKT1-D323H-NL	0.3305	0.2285	0.1628	0.2406	0.0488

E

Calculated K_i values

	Replicate 1 (nM)	Replicate 2 (nM)	Replicate 3 (nM)	Average (nM)	Standard Error
WT-AKT1-NL	59.7221	40.8427	33.7069	44.7572	7.7608
AKT1-E17K-NL	24.4699	15.6137	12.8078	17.6305	3.5143
AKT1-L52R-NL	12.0807	8.6555	7.8386	9.5249	1.2994
AKT1-Q79K-NL	2.1370	5.5438	4.9110	4.1973	1.0462
AKT1-W80R-NL	18.3793	14.3723	10.1641	14.3052	2.3718
AKT1-D323H-NL	7.2455	3.8599	3.3917	4.8324	1.2141

	Replicate 1 (nM)	Replicate 2 (nM)	Replicate 3 (nM)	Average (nM)	Standard Error
WT-AKT1-NL	107.3779	186.1050	75.2309	122.9046	32.9347
AKT1-E17K-NL	49.7001	23.0731	24.9119	32.5617	8.5856
AKT1-L52R-NL	21.0252	13.7882	12.4370	15.7501	2.6662
AKT1-Q79K-NL	29.3391	4.9482	7.2962	13.8611	7.7686
AKT1-W80R-NL	48.7572	21.5524	25.0375	31.7823	8.5468
AKT1-D323H-NL	13.2610	8.7080	9.0171	10.3287	1.4688

	Replicate 1 (nM)	Replicate 2 (nM)	Replicate 3 (nM)	Average (nM)	Standard Error
WT-AKT1-NL	17.3017	6.2573	10.4043	11.3211	3.2210
AKT1-E17K-NL	11.2608	7.9085	8.1720	9.1137	1.0762
AKT1-L52R-NL	5.4017	3.8034	3.7580	4.3210	0.5405
AKT1-Q79K-NL	0.8057	0.7224	0.1517	0.5599	0.2056
AKT1-W80R-NL	9.2035	8.7059	7.3535	8.4210	0.5527
AKT1-D323H-NL	3.0368	1.9873	2.1847	2.4029	0.3220

	Replicate 1 (nM)	Replicate 2 (nM)	Replicate 3 (nM)	Average (nM)	Standard Error
WT-AKT1-NL	0.8188	1.0146	0.8819	0.9051	0.0577
AKT1-E17K-NL	19.8906	15.8383	14.5193	16.7494	1.6161
AKT1-L52R-NL	8.4386	8.2134	6.6723	7.7748	0.5551
AKT1-Q79K-NL	N/A	0.6627	N/A	0.6627	N/A
AKT1-W80R-NL	562.3030	563.5167	518.7822	548.2006	14.7134
AKT1-D323H-NL	15.4976	13.1982	10.0390	12.9116	1.5823

	Replicate 1 (nM)	Replicate 2 (nM)	Replicate 3 (nM)	Average (nM)	Standard Error
WT-AKT1-NL	0.3485	0.5786	0.4052	0.4441	0.0692
AKT1-E17K-NL	3.5202	4.1762	2.0032	3.2332	0.6435
AKT1-L52R-NL	13.4185	13.0370	7.7462	11.4006	1.8305
AKT1-Q79K-NL	13.7816	7.3665	7.9966	9.7149	2.0415
AKT1-W80R-NL	28.4012	27.7597	14.7693	23.6434	4.4409
AKT1-D323H-NL	4.4734	3.7390	1.7930	3.3351	0.7997

	Replicate 1 (nM)	Replicate 2 (nM)	Replicate 3 (nM)	Average (nM)	Standard Error
WT-AKT1-NL	2.1802	3.3886	3.0211	2.8633	0.3577
AKT1-E17K-NL	37.1646	34.9722	29.1199	33.7522	2.4011
AKT1-L52R-NL	22.3865	20.7059	14.2723	19.1216	2.4727
AKT1-Q79K-NL	30.5110	39.5278	20.5704	30.2031	5.4747
AKT1-W80R-NL	606.6908	454.1078	469.7129	510.1705	48.4699
AKT1-D323H-NL	50.5623	34.9576	24.9064	36.8088	7.4639

Figure S2- Replicate data for Figure 3. **(A)** Bioluminescent Imaging of HEK293T cells transfected with pathological AKT mutants. Scale bar indicates 50 μm.**(B)** Signal build-up curves and fitted EC_{50} values using the K5 tracer on 293T cells transfected with AKT1-NL mutants. **(C)** Replicate binding curves for AKT inhibitors on AKT1-NL mutants. **(D)** Fitted apparent IC_{50} values for curves in (B). **(E)** Calculated K_i values derived from EC_{50} and IC_{50} values in (B and Figure S1A) and (D).

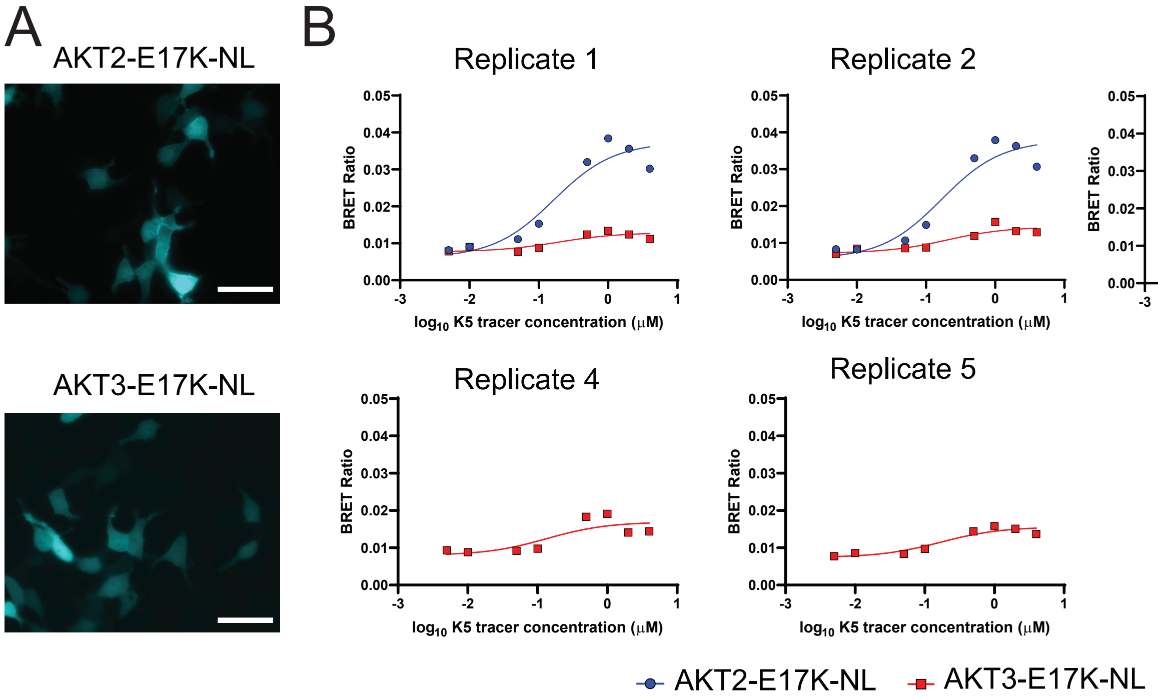

EC50	Replicate 1 (µM)	Replicate 2 (µM)	Replicate 3 (µM)	Replicate 4 (µM)	Replicate 5 (µM)	Average (µM)	Standard Error
AKT2-E17K-NL	0.1643	0.1671	0.1721			0.1678	0.0023
AKT3-E17K-NL	0.1348	0.1698	0.1865	0.1997	0.0873	0.1556	0.0203

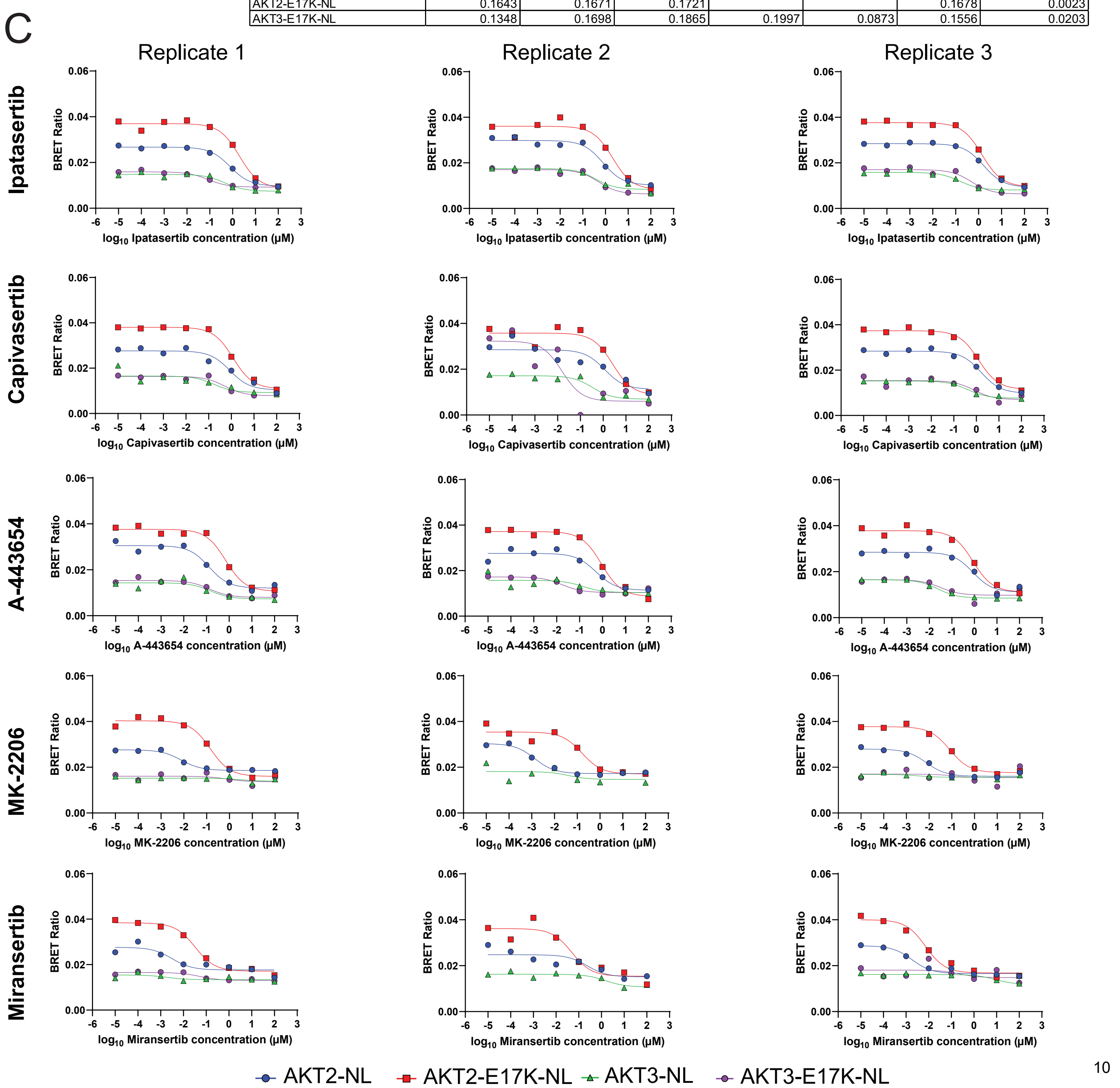

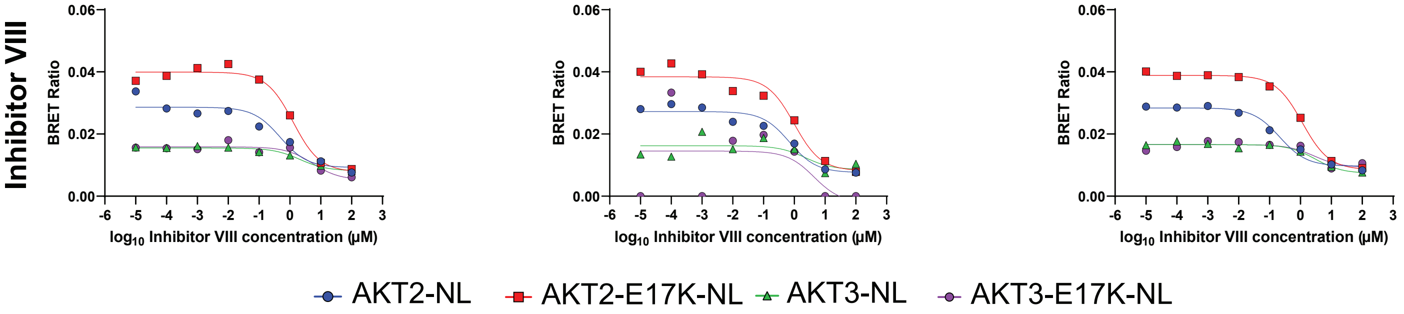

D

Ipatasertib	Replicate 1 (µM)	Replicate 2 (µM)	Replicate 3 (µM)	Average (µM)	Standard Error
AKT2-NL	0.7773	0.7825	1.6560	1.0719	0.2920
AKT2-E17K-NL	1.9840	2.0770	1.4120	1.8243	0.2079
AKT3-NL	0.4538	0.3678	0.1546	0.3254	0.0889
AKT3-E17K-NL	0.0827	0.1434	0.4971	0.2411	0.1292

Capivasertib	Replicate 1 (µM)	Replicate 2 (µM)	Replicate 3 (µM)	Average (µM)	Standard Error
AKT2-NL	0.8630	1.1680	1.6310	1.2207	0.2233
AKT2-E17K-NL	1.2020	2.6300	1.2740	1.7020	0.4645
AKT3-NL	0.1709	0.3384	0.4087	0.3060	0.0705
AKT3-E17K-NL	0.5087	0.0156	0.7948	0.4397	0.2276

A-443654	Replicate 1 (µM)	Replicate 2 (µM)	Replicate 3 (µM)	Average (µM)	Standard Error
AKT2-NL	0.1291	0.5223	0.8603	0.5039	0.2113
AKT2-E17K-NL	0.7003	0.9087	0.8643	0.8244	0.0634
AKT3-NL	0.1496	0.1418	0.0241	0.1052	0.0406
AKT3-E17K-NL	0.1357	0.0175	0.0315	0.0616	0.0373

MK-2206	Replicate 1 (µM)	Replicate 2 (µM)	Replicate 3 (µM)	Average (µM)	Standard Error
AKT2-NL	0.0078	0.0014	0.0076	0.0056	0.0021
AKT2-E17K-NL	0.1427	0.1601	0.0815	0.1281	0.0238
AKT3-NL	N/A	N/A	N/A	N/A	N/A
AKT3-E17K-NL	N/A	N/A	N/A	N/A	N/A

Miransertib	Replicate 1 (µM)	Replicate 2 (µM)	Replicate 3 (µM)	Average (µM)	Standard Error
AKT2-NL	0.0032	0.2368	0.0018	0.0806	0.0781
AKT2-E17K-NL	0.0324	0.0495	0.0073	0.0297	0.0122
AKT3-NL	N/A	1.52	8.70	5.11	3.59
AKT3-E17K-NL	N/A	N/A	N/A	N/A	N/A

Inhibitor VIII	Replicate 1 (µM)	Replicate 2 (µM)	Replicate 3 (µM)	Average (µM)	Standard Error
AKT2-NL	0.4895	0.6986	0.2136	0.4672	0.1404
AKT2-E17K-NL	1.2750	0.9939	1.1500	1.1396	0.0813
AKT3-NL	2.1010	2.2910	3.0170	2.4697	0.2791
AKT3-E17K-NL	5.4410	N/A	2.8120	4.1265	1.3145

E

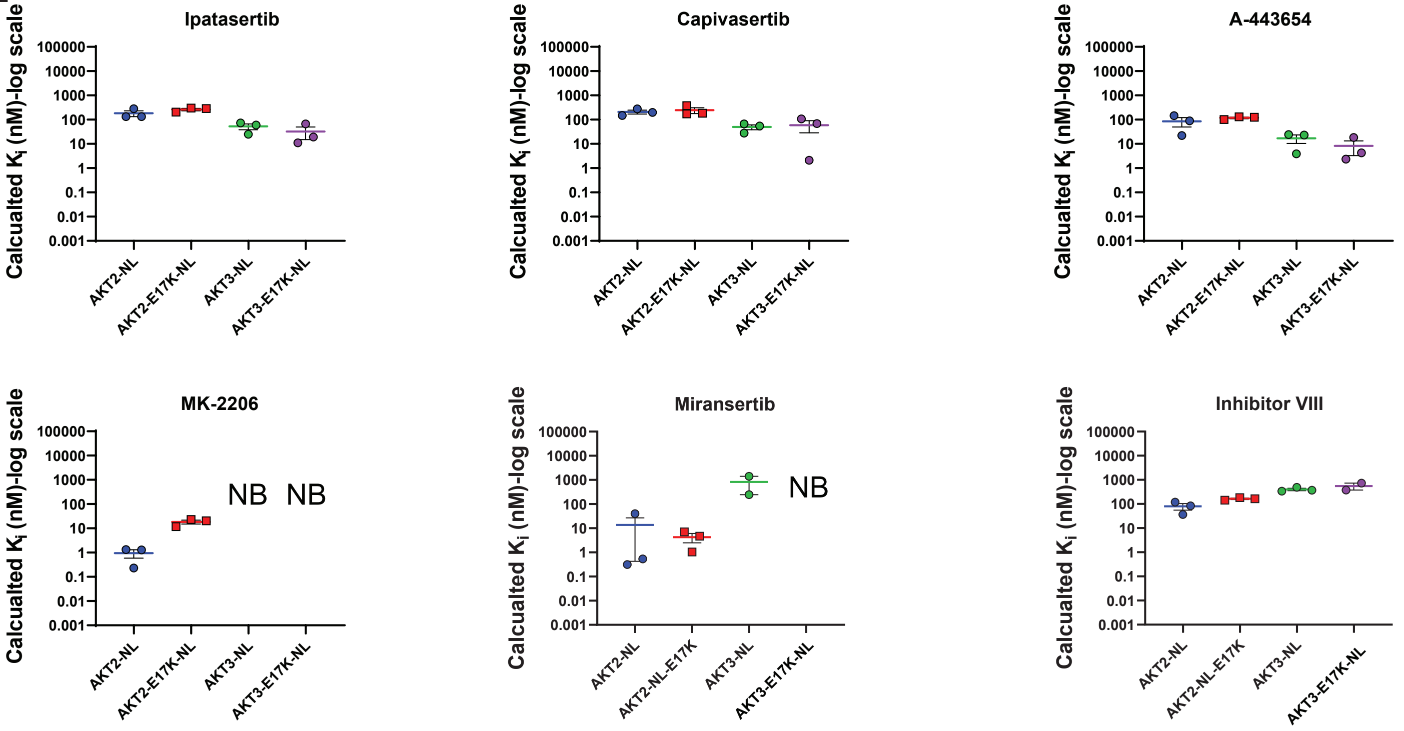

F

Ipatasertib	Replicate 1 (nM)	Replicate 2 (nM)	Replicate 3 (nM)	Average (nM)	Standard Error
AKT2-NL	132.26	133.14	281.76	182.39	49.69
AKT2-E17K-NL	285.13	298.49	202.92	262.18	29.88
AKT3-NL	73.47	59.54	25.03	52.68	14.40
AKT3-E17K-NL	11.13	19.31	66.94	32.46	17.40

Capivasertib	Replicate 1 (nM)	Replicate 2 (nM)	Replicate 3 (nM)	Average (nM)	Standard Error
AKT2-NL	146.84	198.73	277.51	207.69	37.99
AKT2-E17K-NL	172.74	377.97	183.09	244.60	66.75
AKT3-NL	27.67	54.78	66.16	49.54	11.42
AKT3-E17K-NL	68.50	2.10	107.03	59.21	30.64

A-443654	Replicate 1 (nM)	Replicate 2 (nM)	Replicate 3 (nM)	Average (nM)	Standard Error
AKT2-NL	21.97	88.87	146.38	85.74	35.95
AKT2-E17K-NL	100.64	130.59	124.21	118.48	9.11
AKT3-NL	24.22	22.96	3.90	17.03	6.57
AKT3-E17K-NL	18.27	2.36	4.24	8.29	5.02

MK-2206	Replicate 1 (nM)	Replicate 2 (nM)	Replicate 3 (nM)	Average (nM)	Standard Error
AKT2-NL	1.32	0.23	1.28	0.94	0.36
AKT2-E17K-NL	20.51	23.01	11.72	18.41	3.42
AKT3-NL	N/A	N/A	N/A	N/A	N/A
AKT3-E17K-NL	N/A	N/A	N/A	N/A	N/A

Miransertib	Replicate 1 (nM)	Replicate 2 (nM)	Replicate 3 (nM)	Average (nM)	Standard Error
AKT2-NL	0.54	40.29	0.31	13.71	13.29
AKT2-E17K-NL	4.65	7.11	1.04	4.27	1.76
AKT3-NL	N/A	245.91	1409.09	827.50	581.59
AKT3-E17K-NL	N/A	N/A	N/A	N/A	N/A

Inhibitor VIII	Replicate 1 (nM)	Replicate 2 (nM)	Replicate 3 (nM)	Average (nM)	Standard Error
AKT2-NL	83.29	118.86	36.34	79.50	23.90
AKT2-E17K-NL	183.23	142.84	165.27	163.78	11.69
AKT3-NL	340.13	370.89	488.42	399.81	45.18
AKT3-E17K-NL	732.67	N/A	378.66	555.60	177.01

Figure S3- Additional data associated with Figure 3. **(A)** Bioluminescent Imaging of HEK293T cells transfected with AKT2-E17K-NL or AKT3-E17K-NL. Scale bar indicates 50 μm.**(B)** Signal build-up curves and fitted EC_{50} values using the K5 tracer on 293T cells transfected with AKT2-E17K-NL and AKT3-E17K-NL mutants. **(C)** Replicate binding curves for AKT inhibitors on AKT2-NL and AKT3-NL mutants. **(D)** Fitted apparent IC_{50} values for curves in (C). **(E)** Plotted Calculated K_i values derived from EC_{50} and IC_{50} values in (B and Figure S1A) and (D). **(F)** K_i values plotted in (E). Note that "NB" denotes no binding.

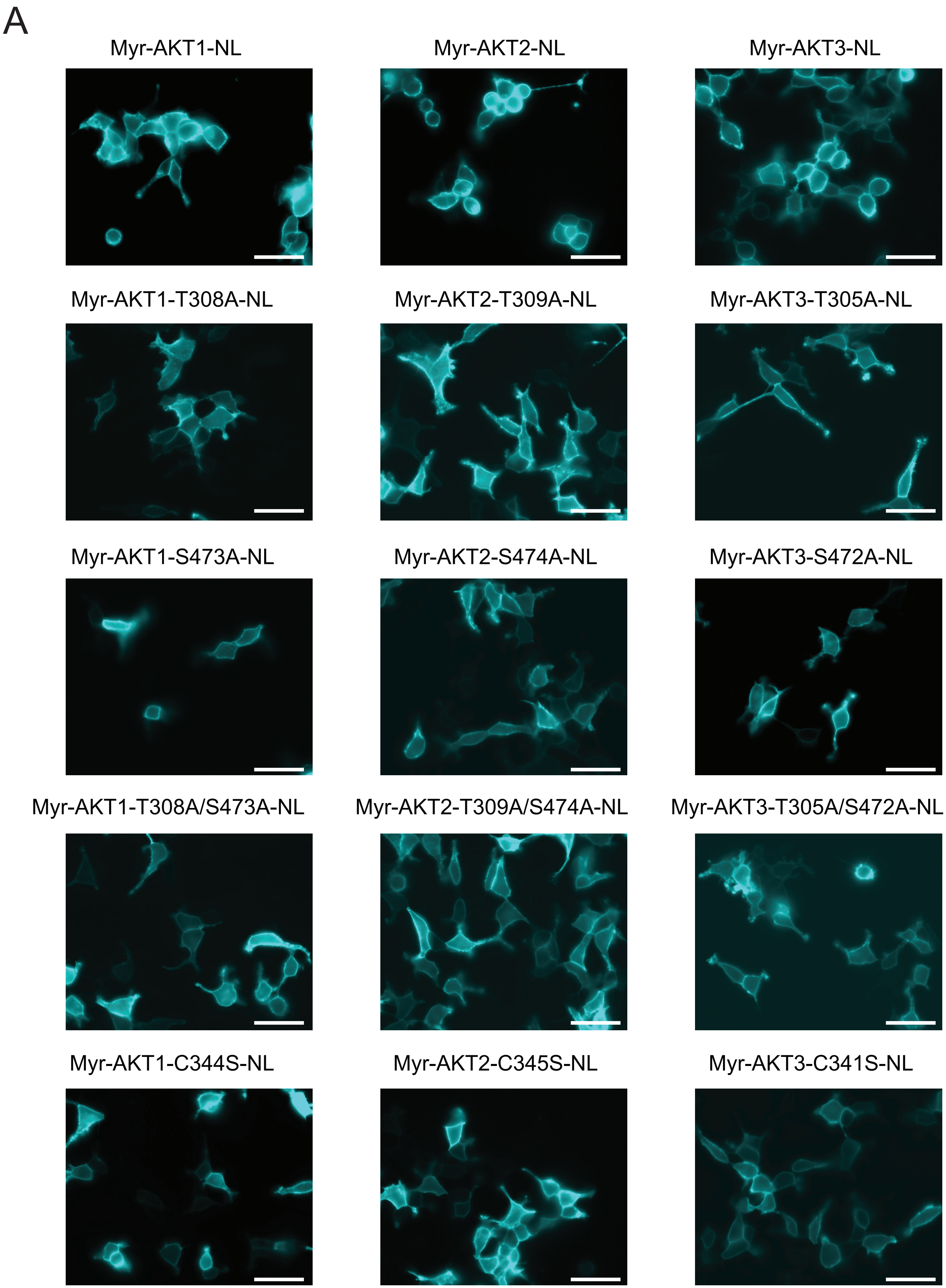
A
Myr-AKT1-NL
Myr-AKT2-NL
Myr-AKT3-NL
Myr-AKT1-T308A-NL
Myr-AKT2-T309A-NL
Myr-AKT3-T305A-NL
Myr-AKT1-S473A-NL
Myr-AKT2-S474A-NL
Myr-AKT3-S472A-NL
Myr-AKT1-T308A/S473A-NL
Myr-AKT2-T309A/S474A-NL
Myr-AKT3-T305A/S472A-NL
Myr-AKT1-C344S-NL
Myr-AKT2-C345S-NL
Myr-AKT3-C341S-NL

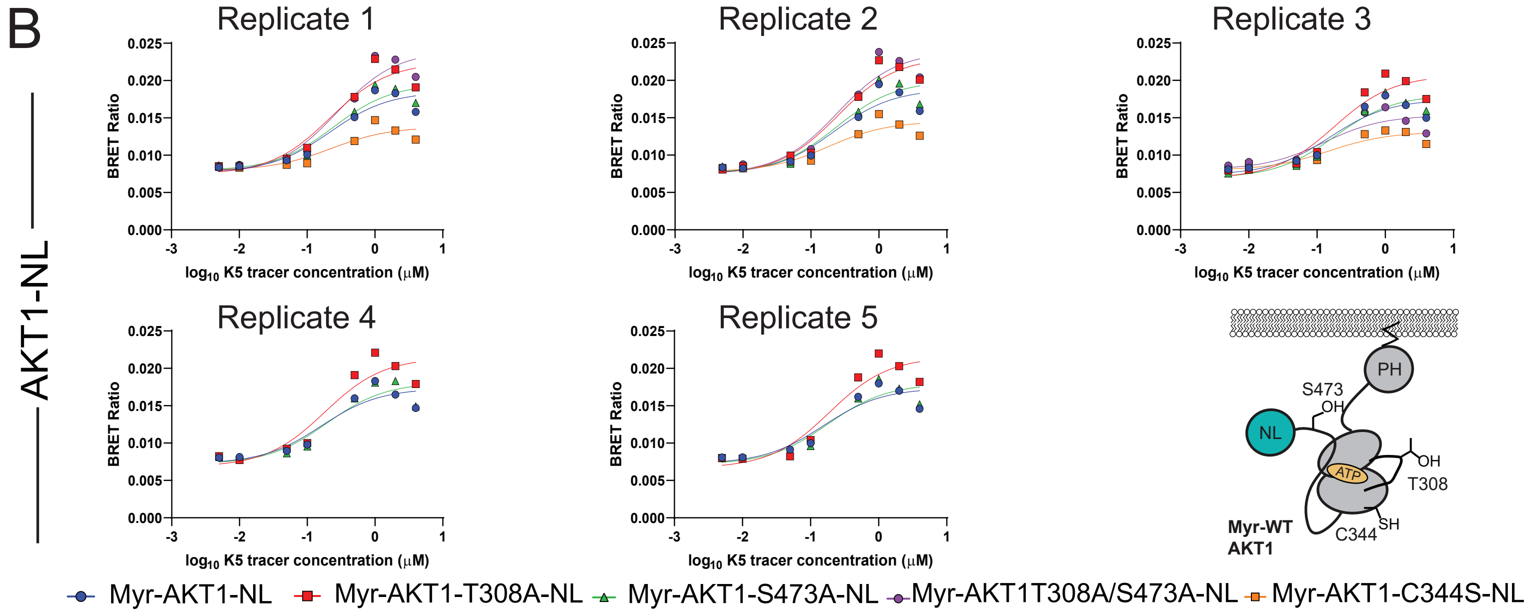

EC$_{50}$ Values

AKT1	Replicate 1(µM)	Replicate 2 (µM)	Replicate 3 (µM)	Replicate 4 (µM)	Replicate 5 (µM)	Average (µM)	Standard Error
Myr-AKT1-NL	0.2196	0.22	0.1546	0.1609	0.1533	0.1817	0.0156
Myr-AKT1-T308A-NL	0.2154	0.2469	0.1778	0.1806	0.1889	0.2019	0.0131
Myr-AKT1-S473A-NL	0.2433	0.2376	0.1829	0.1942	0.1801	0.2076	0.0136
Myr-AKT1-T308A/S473A-NL	0.2766	0.2442	0.1259			0.2156	0.0458
Myr-AKT1-C344S-NL	0.2224	0.1814	0.1486			0.1841	0.0213

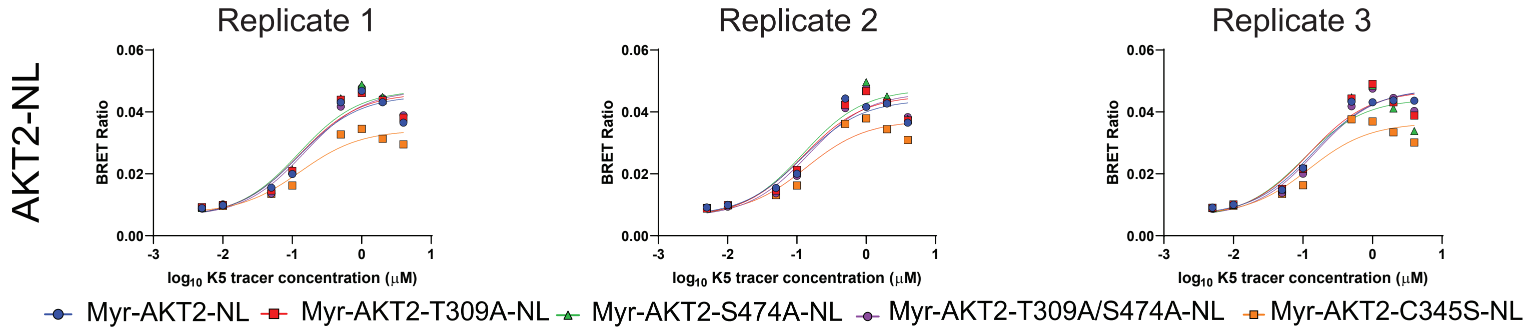

EC$_{50}$ Values

AKT2	Replicate 1(µM)	Replicate 2 (µM)	Replicate 3 (µM)	Average (µM)	Standard Error
Myr-AKT2-NL	0.1237	0.1195	0.1420	0.1284	0.0069
Myr-AKT2-T309A-NL	0.1269	0.1230	0.1215	0.1238	0.0016
Myr-AKT2-S474A-NL	0.1235	0.1247	0.1034	0.1172	0.0069
Myr-AKT2-T309A/S474A-NL	0.1463	0.1458	0.1502	0.1474	0.0014
Myr-AKT2-C345S-NL	0.1304	0.1360	0.1219	0.1294	0.0041

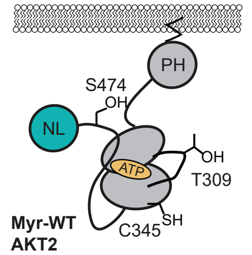

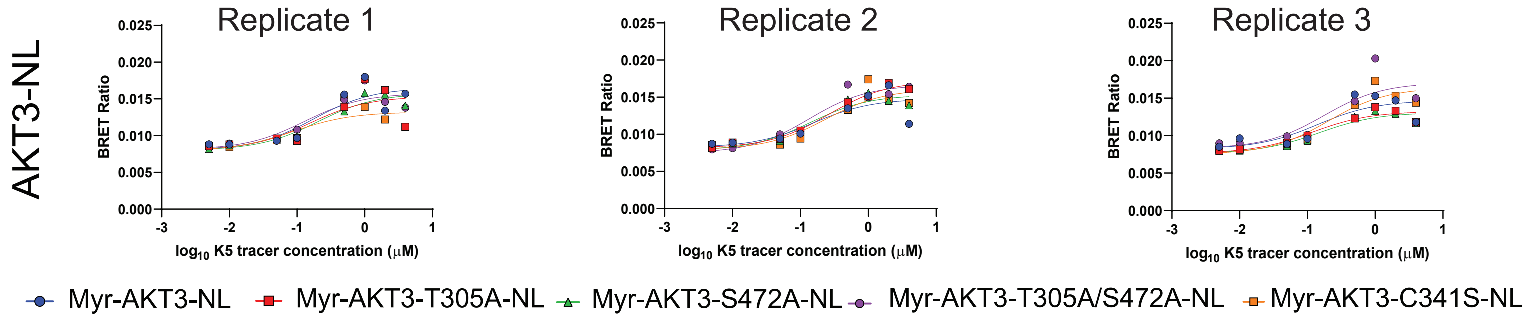

EC$_{50}$ Values

AKT3	Replicate 1(µM)	Replicate 2 (µM)	Replicate 3 (µM)	Average (µM)	Standard Error
Myr-AKT3-NL	0.1766	0.1534	0.14	0.1567	0.0107
Myr-AKT3-T305A-NL	0.1529	0.261	0.116	0.1766	0.0435
Myr-AKT3-S472A-NL	0.2026	0.1392	0.147	0.1629	0.0200
Myr-AKT3-T305A/S472A-NL	0.1293	0.1332	0.1752	0.1459	0.0147
Myr-AKT3-C341S-NL	0.0873	0.2345	0.204	0.1753	0.0449

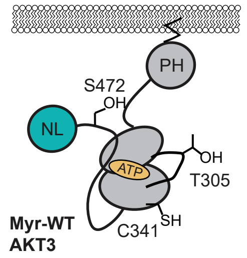

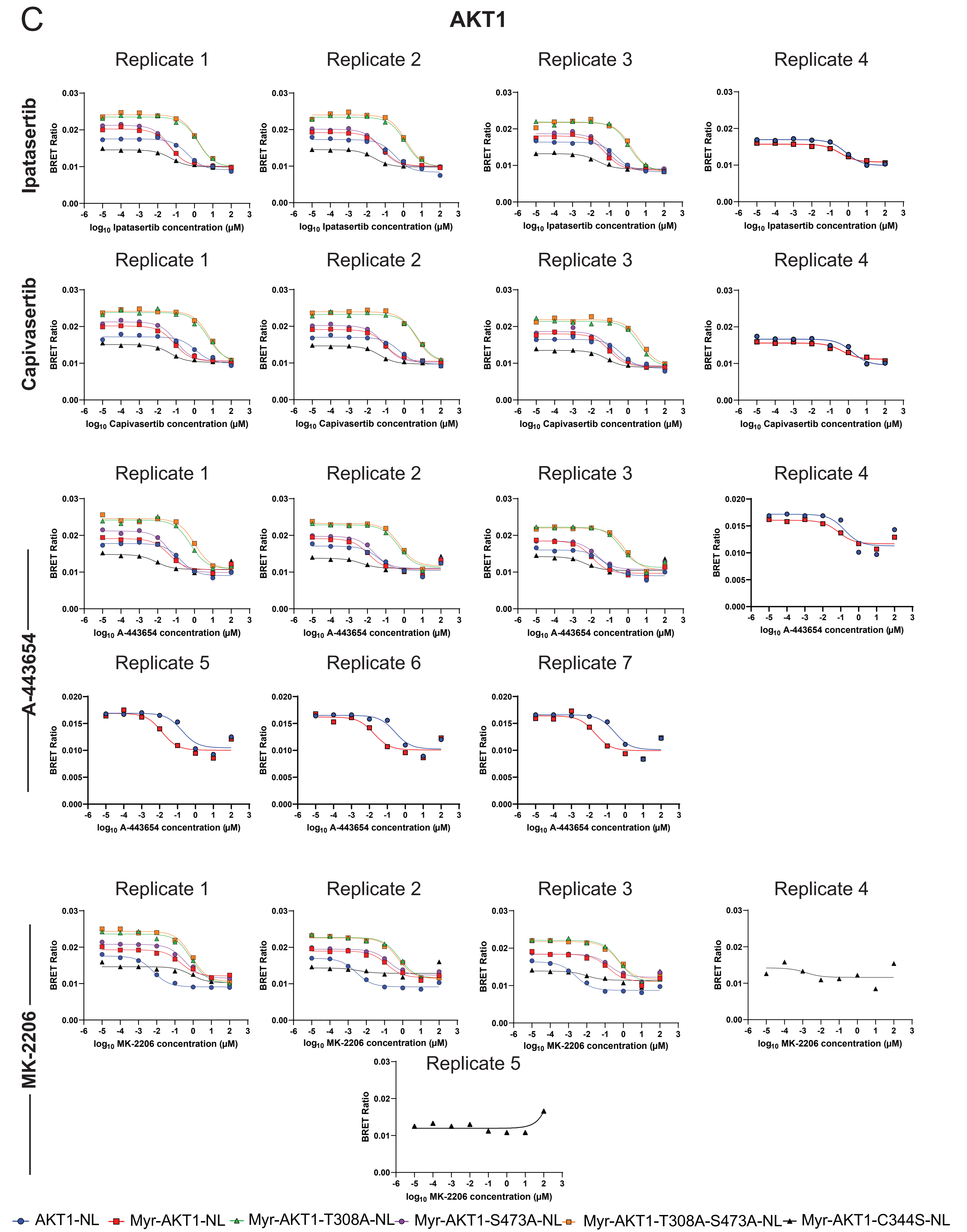
C
AKT1
Ipatasertib
Capivasertib
A-443654
MK-2206
Replicate 1
Replicate 2
Replicate 3
Replicate 4
Replicate 5
Replicate 6
Replicate 7
BRET Ratio
log10 Ipatasertib concentration (µM)
log10 Capivasertib concentration (µM)
log10 A-443654 concentration (µM)
log10 MK-2206 concentration (µM)
AKT1-NL
Myr-AKT1-NL
Myr-AKT1-T308A-NL
Myr-AKT1-S473A-NL
Myr-AKT1-T308A-S473A-NL
Myr-AKT1-C344S-NL

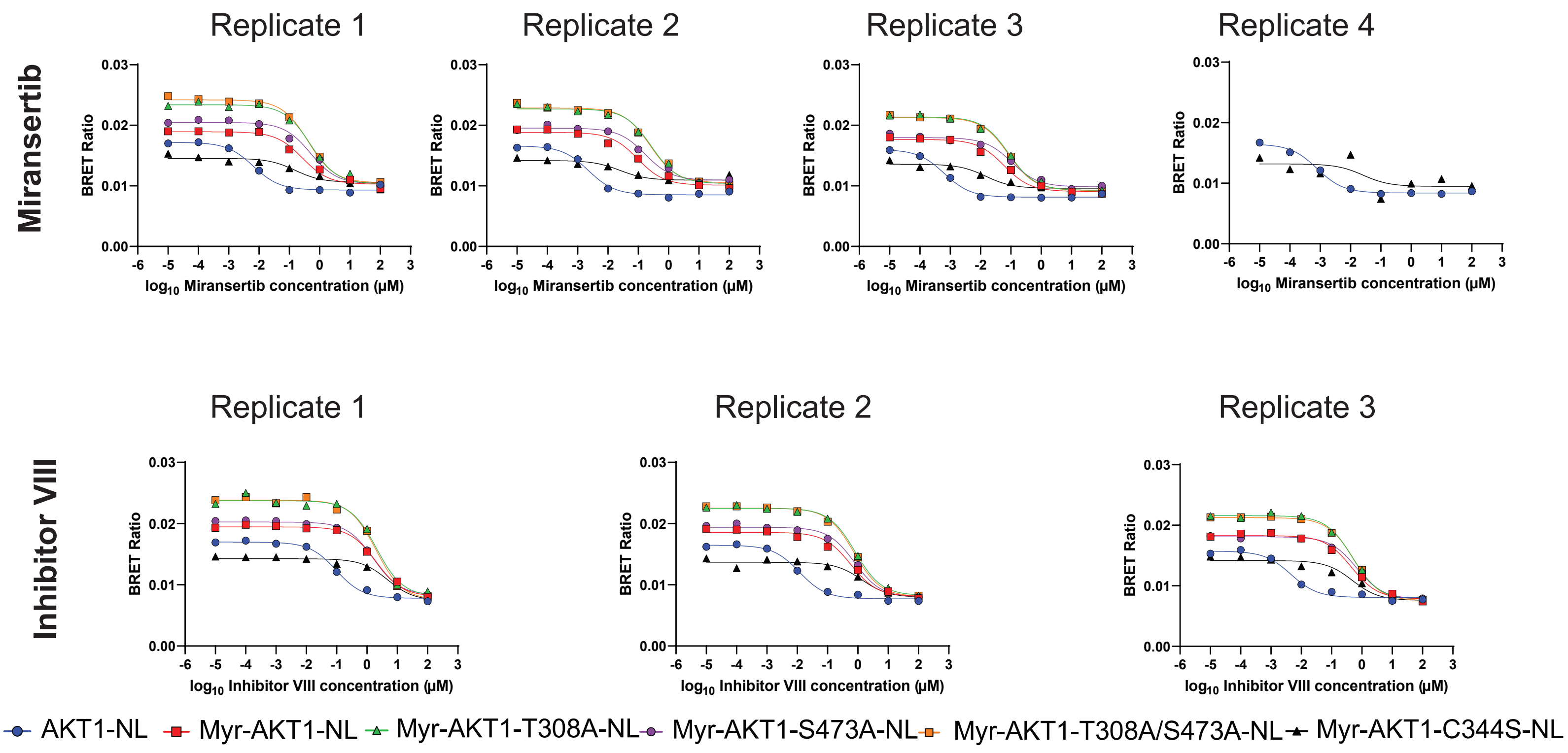

D

Apparent IC_{50} Values

Ipatasertib	Replicate 1 (µM)	Replicate 2 (µM)	Replicate 3 (µM)	Replicate 4 (µM)	Average (µM)	Standard Error
AKT1-NL	0.4446	0.3463	0.2727	0.7315	0.4488	0.1006
Myr-AKT1-NL	0.0502	0.0744	0.0641	0.3554	0.1360	0.0733
Myr-AKT1-T308A-NL	1.5760	1.2390	1.2290		1.3480	0.1140
Myr-AKT1-S473A-NL	0.0454	0.0581	0.0792		0.0609	0.0099
Myr-AKT1-T308A-S473A-NL	1.5320	1.3750	1.5860		1.4977	0.0633
Myr-AKT1-C344S-NL	0.0555	0.0264	0.0247		0.0356	0.0100

Capivasertib	Replicate 1 (µM)	Replicate 2 (µM)	Replicate 3 (µM)	Replicate 4 (µM)	Average (µM)	Standard Error
AKT1-NL	1.1590	0.5347	0.4594	1.6560	0.9523	0.2821
Myr-AKT1-NL	0.0808	0.0979	0.0919	0.4651	0.1839	0.0938
Myr-AKT1-T308A-NL	5.2900	5.4340	4.1430		4.9557	0.4085
Myr-AKT1-S473A-NL	0.1016	0.0792	0.1129		0.0979	0.0099
Myr-AKT1-T308A-S473A-NL	6.5200	4.4800	5.1900		5.3967	0.5979
Myr-AKT1-C344S-NL	0.0488	0.0448	0.0692		0.0543	0.0076

A-443654	Replicate 1 (µM)	Replicate 2 (µM)	Replicate 3 (µM)	Replicate 4 (µM)	Replicate 5 (µM)	Replicate 6 (µM)	Replicate 7 (µM)	Average (µM)	Standard Error
AKT1-NL	0.1704	0.0405	0.0538	0.1650	0.1758	0.2730	0.2575	0.1623	0.0339
Myr-AKT1-NL	0.0403	0.0112	0.0103	0.0740	0.0133	0.0178	0.0206	0.0268	0.0088
Myr-AKT1-T308A-NL	0.6119	0.4913	0.4175					0.5069	0.0567
Myr-AKT1-S473A-NL	0.0486	0.0218	0.0186					0.0297	0.0095
Myr-AKT1-T308A-S473A-NL	1.0160	0.5635	0.6601					0.7465	0.1376
Myr-AKT1-C344S-NL	0.0082	0.0046	0.0046					0.0058	0.0012

MK-2206	Replicate 1 (µM)	Replicate 2 (µM)	Replicate 3 (µM)	Replicate 4 (µM)	Replicate 5 (µM)	Average (µM)	Standard Error
AKT1-NL	0.0065	0.0028	0.0025			0.0039	0.0013
Myr-AKT1-NL	0.2072	0.1358	0.1484			0.1638	0.0220
Myr-AKT1-T308A-NL	0.7748	0.6234	0.5784			0.6589	0.0594
Myr-AKT1-S473A-NL	0.3440	0.1792	0.1719			0.2317	0.0562
Myr-AKT1-T308A-S473A-NL	0.8123	0.5354	0.5055			0.6177	0.0977
Myr-AKT1-C344S-NL	0.7654	0.0040	0.0097	0.0015	0.0555	0.1672	0.1499

Miransertib	Replicate 1 (µM)	Replicate 2 (µM)	Replicate 3 (µM)	Replicate 4 (µM)	Average (µM)	Standard Error
AKT1-NL	0.0065	0.0022	0.0006	0.0008	0.0025	0.0014
Myr-AKT1-NL	0.2706	0.0953	0.0591		0.1417	0.0653
Myr-AKT1-T308A-NL	0.4973	0.2705	0.0855		0.2844	0.1191
Myr-AKT1-S473A-NL	0.4985	0.1694	0.1069		0.2583	0.1215
Myr-AKT1-T308A-S473A-NL	0.4170	0.2454	0.0827		0.2484	0.0965
Myr-AKT1-C344S-NL	0.1571	0.0248	0.0149	0.0261	0.0557	0.0339

Inhibitor VIII	Replicate 1 (µM)	Replicate 2 (µM)	Replicate 3 (µM)	Average (µM)	Standard Error
AKT1-NL	0.0962	0.0117	0.0045	0.0375	0.0294
Myr-AKT1-NL	2.0050	0.5831	0.4534	1.0138	0.4970
Myr-AKT1-T308A-NL	2.0810	0.8179	0.4901	1.1297	0.4850
Myr-AKT1-S473A-NL	1.6180	0.7689	0.6730	1.0200	0.3003
Myr-AKT1-T308A-S473A-NL	1.9970	0.7452	0.5350	1.0924	0.4564
Myr-AKT1-C344S-NL	4.7720	1.3600	0.4882	2.2067	1.3071

E

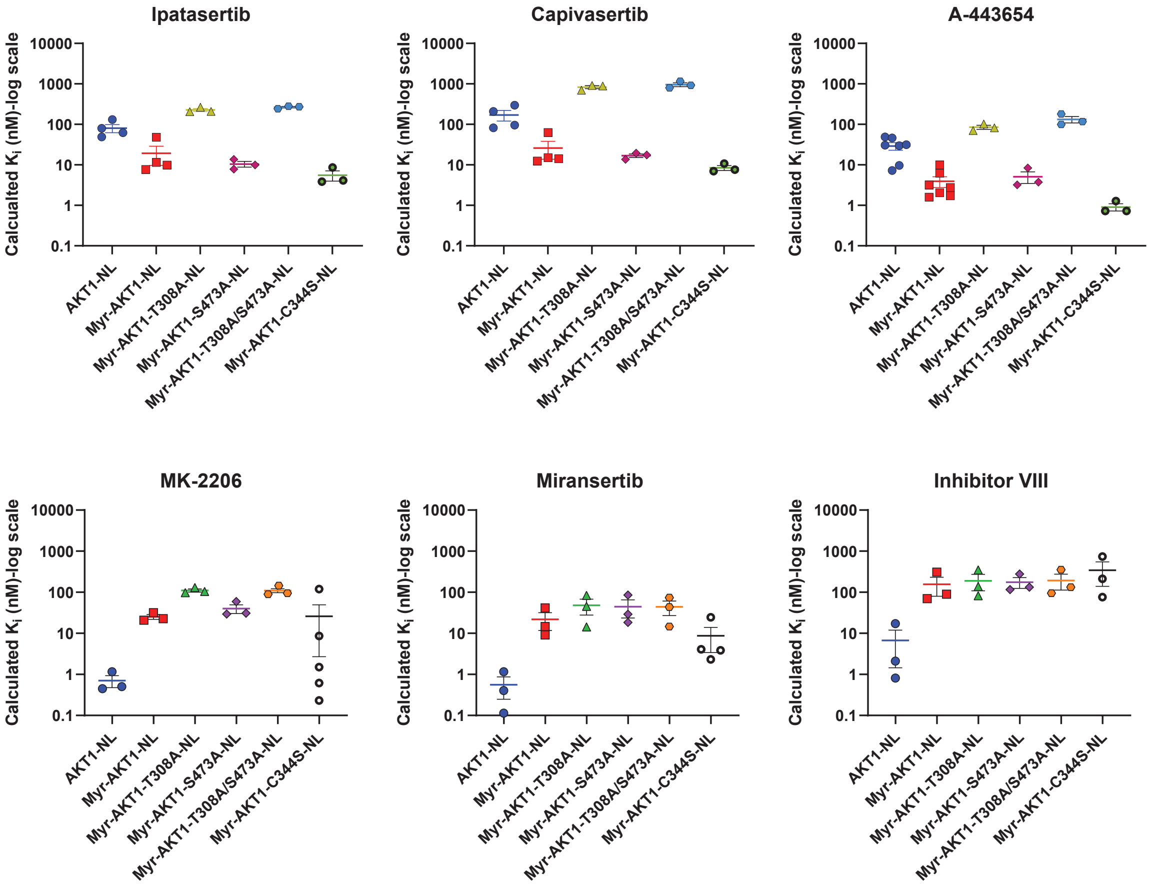

F

Calculated K_i Values

Ipatasertib	Replicate 1 (nM)	Replicate 2 (nM)	Replicate 3 (nM)	Replicate 4 (nM)	Average (nM)	Standard Error
AKT1-NL	79.71	62.09	48.89	131.15	80.46	18.04
Myr-AKT1-NL	7.713	11.44	9.86	47.86	19.22	9.58
Myr-AKT1-T308A-NL	264.76	208.15	206.47		226.46	19.16
Myr-AKT1-S473A-NL	7.81	10.00	13.62		10.48	1.69
Myr-AKT1-T308A-S473A-NL	271.68	243.84	281.26		265.59	11.22
Myr-AKT1-C344S-NL	8.64	4.10	3.84		5.53	1.56

Capivasertib	Replicate 1 (nM)	Replicate 2 (nM)	Replicate 3 (nM)	Replicate 4 (nM)	Average (nM)	Standard Error
AKT1-NL	207.80	95.87	82.37	296.91	170.74	50.59
Myr-AKT1-NL	12.42	15.05	14.13	62.63	26.06	12.20
Myr-AKT1-T308A-NL	888.71	912.90	696.02		832.54	68.62
Myr-AKT1-S473A-NL	17.47	13.62	19.41		16.83	1.70
Myr-AKT1-T308A-S473A-NL	1156.25	794.48	920.39		957.04	106.03
Myr-AKT1-C344S-NL	7.59	6.96	10.76		8.44	1.18

A-443654	Replicate 1 (nM)	Replicate 2 (nM)	Replicate 3 (nM)	Replicate 4 (nM)	Replicate 5 (nM)	Replicate 6 (nM)	Replicate 7 (nM)	Average (nM)	Standard Error
AKT1-NL	30.55	7.26	9.65	29.58	31.52	48.95	46.17	29.10	6.07
Myr-AKT1-NL	6.19	1.72	1.59	9.96	2.05	2.74	3.17	3.92	1.17
Myr-AKT1-T308A-NL	102.80	82.54	70.14					85.16	9.52
Myr-AKT1-S473A-NL	8.35	3.75	3.19					5.10	1.63
Myr-AKT1-T308A-S473A-NL	180.18	99.93	117.06					132.39	24.40
Myr-AKT1-C344S-NL	1.28	0.72	0.72					0.91	0.19

MK-2206	Replicate 1 (nM)	Replicate 2 (nM)	Replicate 3 (nM)	Replicate 4 (nM)	Replicate 5 (nM)	Average (nM)	Standard Error
AKT1-NL	1.16	0.51	0.45			0.70	0.23
Myr-AKT1-NL	31.86	20.88	22.82			25.18	3.38
Myr-AKT1-T308A-NL	130.16	104.73	97.17			110.69	9.98
Myr-AKT1-S473A-NL	59.14	30.81	29.55			39.84	9.66
Myr-AKT1-T308A-S473A-NL	144.05	94.95	89.64			109.55	17.32
Myr-AKT1-C344S-NL	119.02	0.61	1.51	0.23	8.63	26.00	23.31

Miransertib	Replicate 1 (nM)	Replicate 2 (nM)	Replicate 3 (nM)	Replicate 4 (nM)	Average (nM)	Standard Error
AKT1-NL	1.17	0.40	0.11	0.14	0.56	0.31
Myr-AKT1-NL	41.60	14.65	9.09		21.78	10.04
Myr-AKT1-T308A-NL	83.55	45.44	14.37		47.79	20.00
Myr-AKT1-S473A-NL	85.70	29.12	18.38		44.40	20.88
Myr-AKT1-T308A-S473A-NL	73.95	43.52	14.67		44.05	17.11
Myr-AKT1-C344S-NL	24.43	3.85	2.32	4.06	8.66	5.27

Inhibitor VIII	Replicate 1 (nM)	Replicate 2 (nM)	Replicate 3 (nM)	Average (nM)	Standard Error
AKT1-NL	17.24	2.10	0.81	6.72	5.28
Myr-AKT1-NL	308.26	89.65	69.71	155.87	76.41
Myr-AKT1-T308A-NL	349.60	137.41	82.34	189.78	81.48
Myr-AKT1-S473A-NL	278.17	132.19	115.71	175.36	51.63
Myr-AKT1-T308A-S473A-NL	354.14	132.15	94.88	193.72	80.93
Myr-AKT1-C344S-NL	742.05	211.48	75.92	343.15	203.25

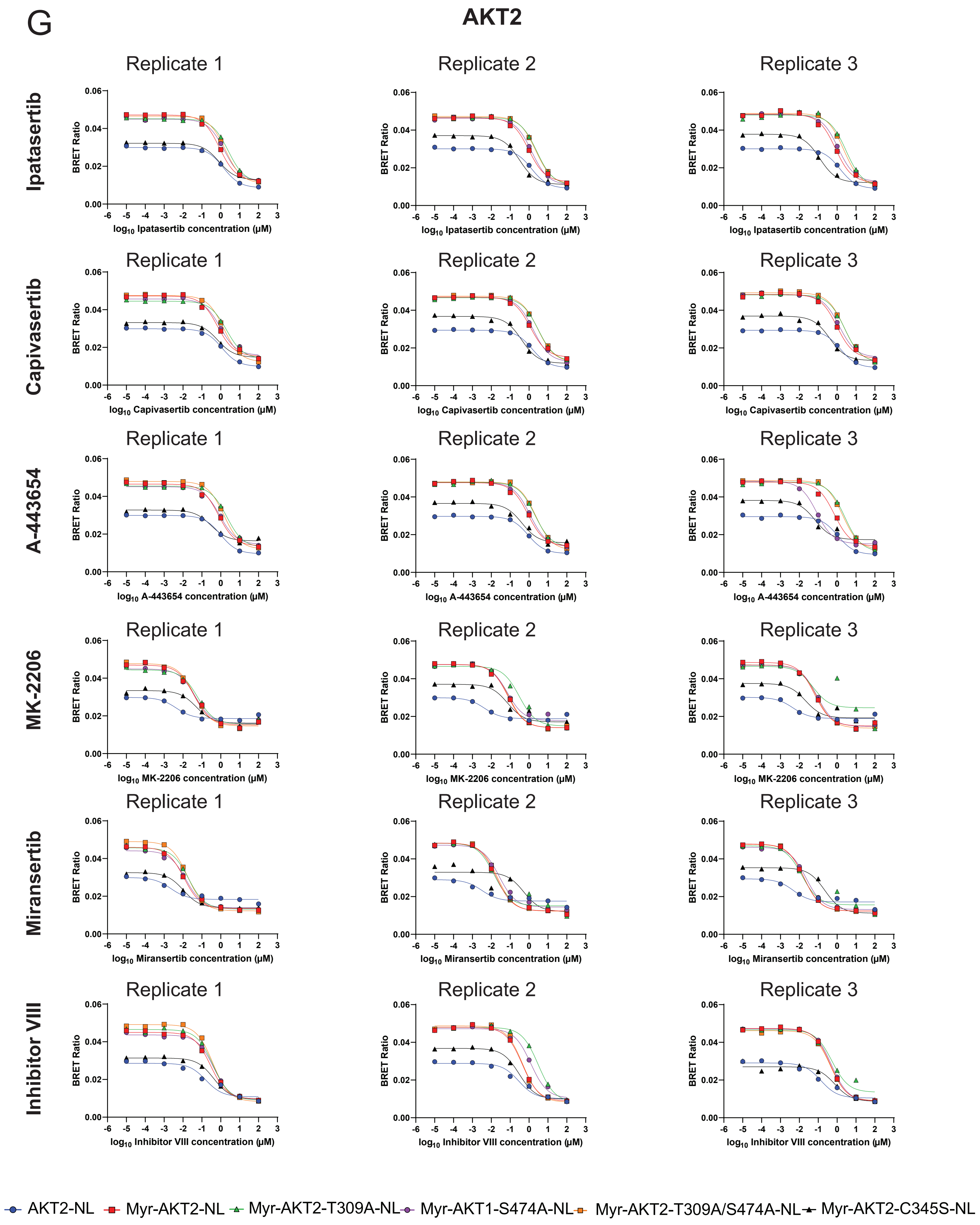
G
AKT2
Replicate 1
Replicate 2
Replicate 3
Ipatasertib
Capivasertib
A-443654
MK-2206
Miransertib
Inhibitor VIII
BRET Ratio
0.06
0.04
0.02
0.00
-6 -5 -4 -3 -2 -1 0 1 2 3
log10 Ipatasertib concentration (µM)
log10 Capivasertib concentration (µM)
log10 A-443654 concentration (µM)
log10 MK-2206 concentration (µM)
log10 Miransertib concentration (µM)
log10 Inhibitor VIII concentration (µM)
AKT2-NL
Myr-AKT2-NL
Myr-AKT2-T309A-NL
Myr-AKT1-S474A-NL
Myr-AKT2-T309A/S474A-NL
Myr-AKT2-C345S-NL

H Apparent IC_{50} Values

Ipatasertib	Replicate 1 (μM)	Replicate 2 (μM)	Replicate 3 (μM)	Average (μM)	Standard Error
AKT2-NL	1.3790	1.3440	1.4020	1.3750	0.0169
Myr-AKT2-NL	0.8675	0.9573	0.8078	0.8775	0.0434
Myr-AKT2-T309A-NL	2.4750	2.5710	2.9760	2.6740	0.1535
Myr-AKT2-S474A-NL	1.5160	1.3790	1.1420	1.3457	0.1092
Myr-AKT2-T309A-S474A-NL	1.7490	2.2480	2.2410	2.0793	0.1652
Myr-AKT2-C345S-NL	0.8083	0.3157	0.1203	0.4148	0.2047

Capivasertib	Replicate 1 (μM)	Replicate 2 (μM)	Replicate 3 (μM)	Average (μM)	Standard Error
AKT2-NL	1.1280	1.4710	1.4990	1.3660	0.1193
Myr-AKT2-NL	0.6666	1.1720	0.9790	0.9392	0.1472
Myr-AKT2-T309A-NL	1.9980	3.0850	2.5290	2.5373	0.3138
Myr-AKT2-S474A-NL	0.9233	1.6560	1.2920	1.2904	0.2115
Myr-AKT2-T309A-S474A-NL	1.4550	2.9200	2.3510	2.2420	0.4264
Myr-AKT2-C345S-NL	0.6808	0.4054	0.4076	0.4979	0.0914

A-443654	Replicate 1 (μM)	Replicate 2 (μM)	Replicate 3 (μM)	Average (μM)	Standard Error
AKT2-NL	0.9754	0.8890	1.0780	0.9808	0.0546
Myr-AKT2-NL	0.7544	0.8746	0.7114	0.7801	0.0488
Myr-AKT2-T309A-NL	2.0840	2.0060	2.5390	2.2097	0.1662
Myr-AKT2-S474A-NL	0.8956	1.1160	0.0840	0.6985	0.3138
Myr-AKT2-T309A-S474A-NL	1.4820	2.2940	1.9520	1.9093	0.2354
Myr-AKT2-C345S-NL	0.3088	0.4220	0.0680	0.2663	0.1044

MK-2206	Replicate 1 (μM)	Replicate 2 (μM)	Replicate 3 (μM)	Average (μM)	Standard Error
AKT2-NL	0.0044	0.0044	0.0046	0.0044	0.0001
Myr-AKT2-NL	0.0377	0.0759	0.0644	0.0593	0.0113
Myr-AKT2-T309A-NL	0.0587	0.3011	0.0524	0.1374	0.0819
Myr-AKT2-S474A-NL	0.0440	0.0705	0.0856	0.0667	0.0122
Myr-AKT2-T309A-S474A-NL	0.0396	0.0818	0.0830	0.0681	0.0143
Myr-AKT2-C345S-NL	0.0501	0.0734	0.0213	0.0483	0.0151

Miransertib	Replicate 1 (μM)	Replicate 2 (μM)	Replicate 3 (μM)	Average (μM)	Standard Error
AKT2-NL	0.0022	0.0031	0.0048	0.0034	0.0007
Myr-AKT2-NL	0.0113	0.0183	0.0174	0.0157	0.0022
Myr-AKT2-T309A-NL	0.0196	0.0139	0.0136	0.0157	0.0019
Myr-AKT2-S474A-NL	0.0141	0.0257	0.0231	0.0210	0.0035
Myr-AKT2-T309A-S474A-NL	0.0172	0.0209	0.0173	0.0185	0.0012
Myr-AKT2-C345S-NL	0.0105	0.5261	0.2255	0.2540	0.1495

Inhibitor VIII	Replicate 1 (μM)	Replicate 2 (μM)	Replicate 3 (μM)	Average (μM)	Standard Error
AKT2-NL	0.1617	0.2822	0.1373	0.1937	0.0448
Myr-AKT2-NL	0.2909	0.4360	0.4450	0.3906	0.0499
Myr-AKT2-T309A-NL	0.3852	3.0730	0.5332	1.3305	0.8723
Myr-AKT2-S474A-NL	0.4180	1.2280	0.5092	0.7184	0.2562
Myr-AKT2-T309A-S474A-NL	0.3821	0.4589	0.4408	0.4273	0.0232
Myr-AKT2-C345S-NL	0.4216	0.2956	0.7540	0.4904	0.1367

I

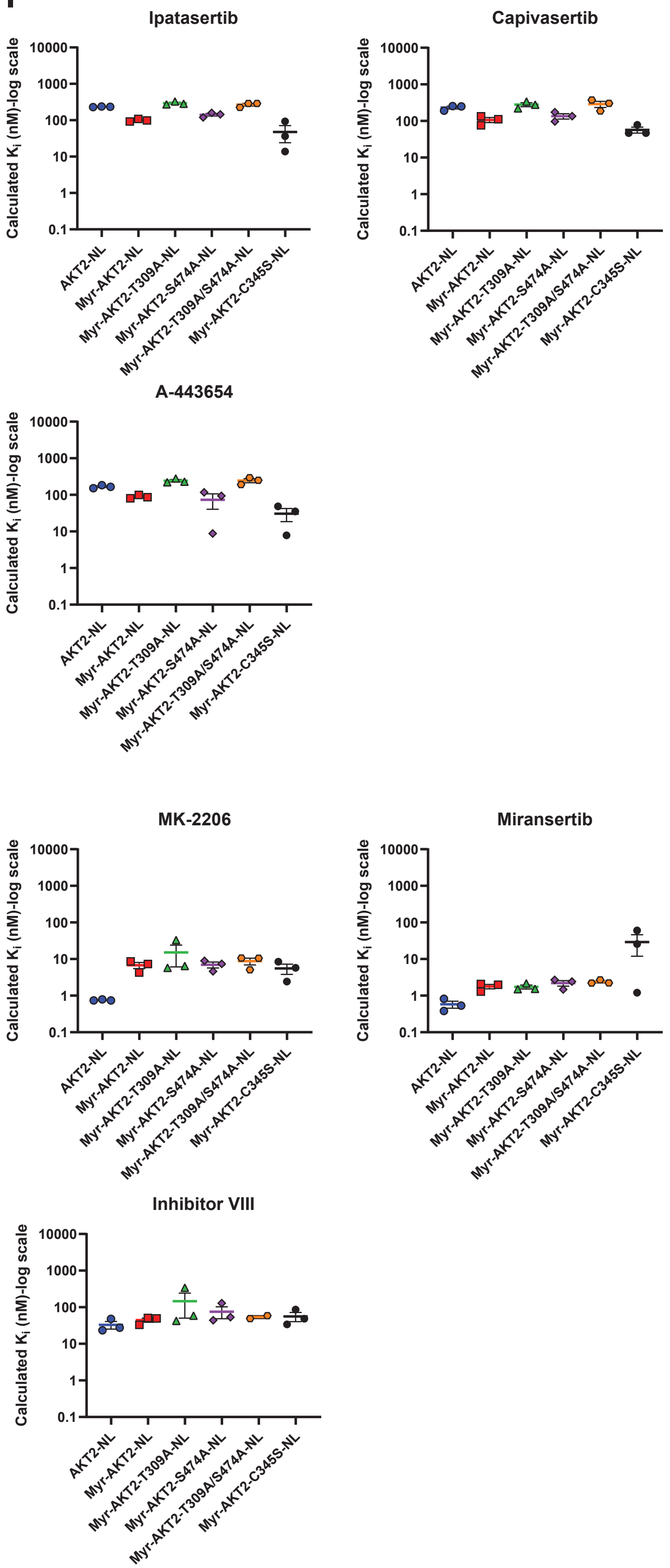

J Calculated K_i Values

Ipatasertib	Replicate 1 (nM)	Replicate 2 (nM)	Replicate 3 (nM)	Average (nM)	Standard Error
AKT2-NL	234.6333	228.6782	238.5467	233.9527	2.8691
Myr-AKT2-NL	98.7123	108.9306	91.9191	99.8540	4.9439
Myr-AKT2-T309A-NL	272.6508	283.2264	327.8420	294.5731	16.9123
Myr-AKT2-S474A-NL	159.0362	144.6642	119.8016	141.1673	11.4602
Myr-AKT2-T309A-S474A-NL	224.7284	288.8448	287.9454	267.1729	21.2238
Myr-AKT2-C345S-NL	92.6314	36.1793	13.7864	47.5324	23.4578

Capivasertib	Replicate 1 (nM)	Replicate 2 (nM)	Replicate 3 (nM)	Average (nM)	Standard Error
AKT2-NL	191.9263	250.2869	255.0510	232.4214	20.2942
Myr-AKT2-NL	75.8520	133.3612	111.3999	106.8710	16.7552
Myr-AKT2-T309A-NL	220.1036	339.8496	278.5996	279.5176	34.5708
Myr-AKT2-S474A-NL	96.8589	173.7229	135.5374	135.3731	22.1889
Myr-AKT2-T309A-S474A-NL	186.9525	375.1898	302.0792	288.0738	54.7888
Myr-AKT2-C345S-NL	78.0198	46.4589	46.7111	57.0633	10.4785

A-443654	Replicate 1 (nM)	Replicate 2 (nM)	Replicate 3 (nM)	Average (nM)	Standard Error
AKT2-NL	165.9618	151.2611	183.4189	166.8806	9.2945
Myr-AKT2-NL	85.8428	99.5202	80.9498	88.7709	5.5572
Myr-AKT2-T309A-NL	229.5775	220.9849	279.7012	243.4212	18.3088
Myr-AKT2-S474A-NL	93.9530	117.0741	8.8162	73.2811	32.9162
Myr-AKT2-T309A-S474A-NL	190.4217	294.7553	250.8118	245.3296	30.2430
Myr-AKT2-C345S-NL	35.3886	48.3613	7.7951	30.5150	11.9613

MK-2206	Replicate 1 (nM)	Replicate 2 (nM)	Replicate 3 (nM)	Average (nM)	Standard Error
AKT2-NL	0.7408	0.7408	0.7888	0.7568	0.0160
Myr-AKT2-NL	4.2933	8.6378	7.3258	6.7523	1.2865
Myr-AKT2-T309A-NL	6.4654	33.1698	5.7769	15.1374	9.0184
Myr-AKT2-S474A-NL	4.6106	7.3969	8.9778	6.9951	1.2766
Myr-AKT2-T309A-S474A-NL	5.0895	10.5053	10.6595	8.7514	1.8315
Myr-AKT2-C345S-NL	5.7369	8.4162	2.4375	5.5302	1.7290

Miransertib	Replicate 1 (nM)	Replicate 2 (nM)	Replicate 3 (nM)	Average (nM)	Standard Error
AKT2-NL	0.3827	0.5358	0.8182	0.5789	0.1276
Myr-AKT2-NL	1.2892	2.0801	1.9799	1.7831	0.2486
Myr-AKT2-T309A-NL	2.1581	1.5357	1.4993	1.7310	0.2138
Myr-AKT2-S474A-NL	1.4760	2.6992	2.4223	2.1992	0.3703
Myr-AKT2-T309A-S474A-NL	2.2139	2.6880	2.2216	2.3745	0.1568
Myr-AKT2-C345S-NL	1.2067	60.2912	25.8424	29.1134	17.1344

Inhibitor VIII	Replicate 1 (nM)	Replicate 2 (nM)	Replicate 3 (nM)	Average (nM)	Standard Error
AKT2-NL	27.5128	48.0156	23.3612	32.9632	7.6210
Myr-AKT2-NL	33.1013	49.6122	50.6363	44.4499	5.6820
Myr-AKT2-T309A-NL	42.4344	338.5277	58.7384	146.5668	96.0958
Myr-AKT2-S474A-NL	43.8503	128.8235	53.4177	75.3638	26.8721
Myr-AKT2-T309A-S474A-NL	49.0959	58.9639	56.6382	54.8994	2.9784
Myr-AKT2-C345S-NL	48.3155	33.8758	86.4086	56.2000	15.6689

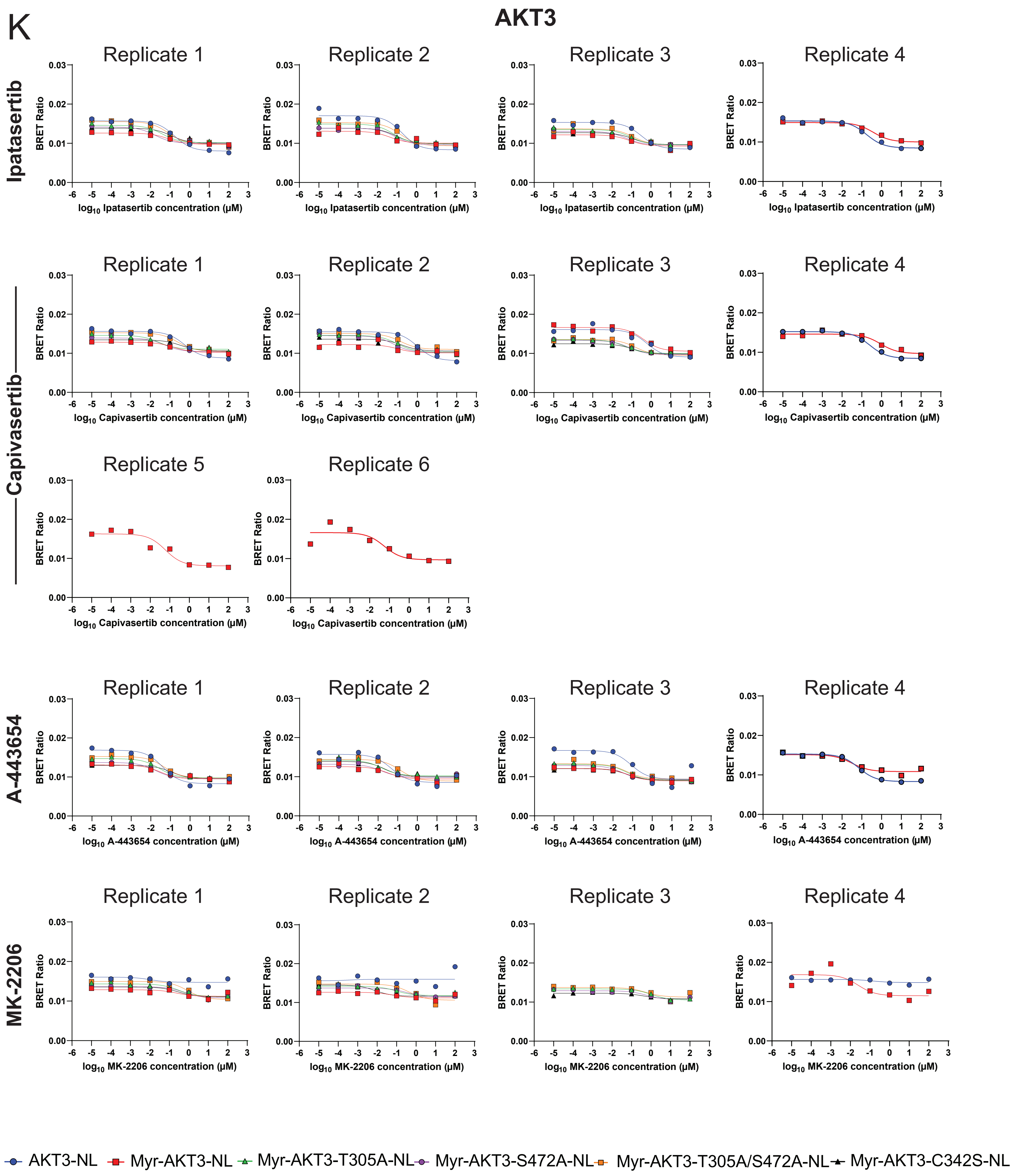
K
AKT3
Ipatasertib
Capivasertib
A-443654
MK-2206
Replicate 1
Replicate 2
Replicate 3
Replicate 4
Replicate 5
Replicate 6
BRET Ratio
log10 Ipatasertib concentration (µM)
log10 Capivasertib concentration (µM)
log10 A-443654 concentration (µM)
log10 MK-2206 concentration (µM)
AKT3-NL
Myr-AKT3-NL
Myr-AKT3-T305A-NL
Myr-AKT3-S472A-NL
Myr-AKT3-T305A/S472A-NL
Myr-AKT3-C342S-NL

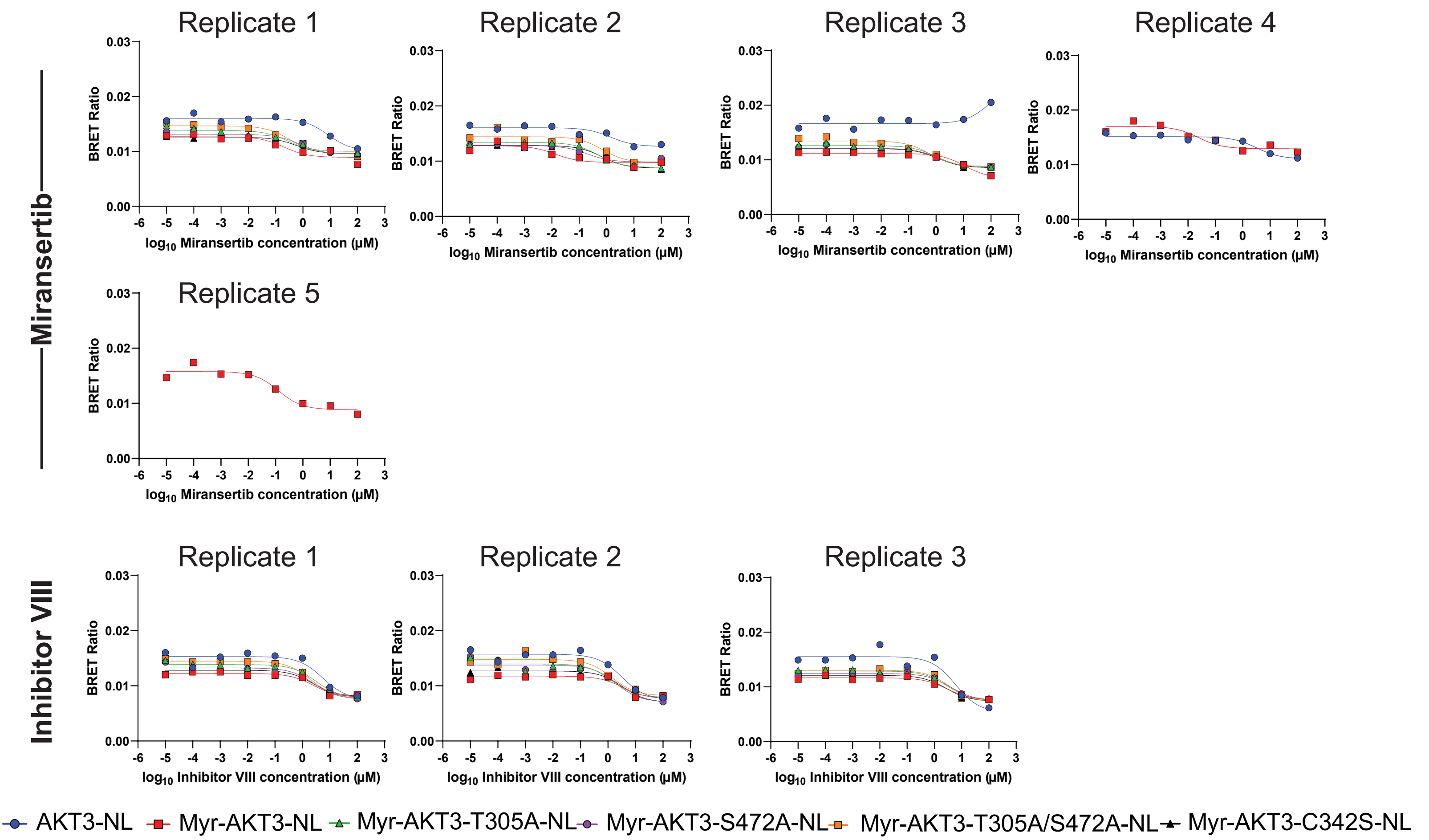

L

Apparent IC_{50} Values

Ipatasertib	Replicate 1 (µM)	Replicate 2 (µM)	Replicate 3 (µM)	Replicate 4 (µM)	Average (µM)	Standard Error
AKT3-NL	0.169	0.017	0.319	0.173	0.170	0.062
Myr-AKT3-NL	0.080	0.013	0.078	0.379	0.138	0.082
Myr-AKT3-T305A-NL	0.041	0.015	0.093		0.050	0.023
Myr-AKT3-S473A-NL	0.017	0.014	0.052		0.028	0.012
Myr-AKT3-T305A-S473A-NL	0.079	0.015	0.127		0.074	0.032
Myr-AKT3-C342S-NL	0.115	0.014	0.114		0.080	0.034

Capivasertib	Replicate 1 (µM)	Replicate 2 (µM)	Replicate 3 (µM)	Replicate 4 (µM)	Replicate 5 (µM)	Replicate 6 (µM)	Average (µM)	Standard Error
AKT3-NL	0.562	0.998	0.598	0.250			0.602	0.154
Myr-AKT3-NL	0.164	0.097	15.770	0.059	0.056	0.892	2.840	2.589
Myr-AKT3-T305A-NL	0.027	0.094	0.049				0.057	0.020
Myr-AKT3-S473A-NL	0.053	0.061	0.074				0.063	0.006
Myr-AKT3-T305A-S473A-NL	0.251	0.139	0.159				0.183	0.035
Myr-AKT3-C342S-NL	0.360	0.166	0.109				0.212	0.076

A-443654	Replicate 1 (µM)	Replicate 2 (µM)	Replicate 3 (µM)	Replicate 4 (µM)	Average (µM)	Standard Error
AKT3-NL	0.041	0.047	0.084	0.070	0.061	0.010
Myr-AKT3-NL	0.030	0.031	0.062	0.032	0.039	0.008
Myr-AKT3-T305A-NL	0.027	0.016	0.053		0.032	0.011
Myr-AKT3-S473A-NL	0.018	0.015	0.032		0.022	0.006
Myr-AKT3-T305A-S473A-NL	0.050	0.136	0.049		0.078	0.029
Myr-AKT3-C342S-NL	0.114	0.023	0.052		0.063	0.027

MK-2206	Replicate 1 (µM)	Replicate 2 (µM)	Replicate 3 (µM)	Average (µM)	Standard Error
AKT3-NL	0.0108	0.0004	0.1361	0.0491	0.0436
Myr-AKT3-NL	0.231	0.065	0.025	0.107	0.063
Myr-AKT3-T305A-NL	0.306	0.091	1.038	0.478	0.287
Myr-AKT3-S473A-NL	0.571	0.314	0.583	0.489	0.088
Myr-AKT3-T305A-S473A-NL	0.847	0.453	0.535	0.612	0.120
Myr-AKT3-C342S-NL	0.285	0.008	0.425	0.239	0.123

Miransertib	Replicate 1 (µM)	Replicate 2 (µM)	Replicate 3 (µM)	Replicate 4 (µM)	Replicate 5 (µM)	Average (µM)	Standard Error
AKT3-NL	8.36	1.41	92.45	3.17	11.74	23.42	17.35
Myr-AKT3-NL	0.203	0.013	11.020	0.024		2.815	2.735
Myr-AKT3-T305A-NL	0.279	0.489	0.989			0.586	0.21
Myr-AKT3-S473A-NL	0.790	0.195	1.198			0.727	0.291
Myr-AKT3-T305A-S473A-NL	0.337	0.728	0.585			0.550	0.114
Myr-AKT3-C342S-NL	0.578	0.688	1.134			0.800	0.170

Inhibitor VIII	Replicate 1 (µM)	Replicate 2 (µM)	Replicate 3 (µM)	Average (µM)	Standard Error
AKT3-NL	5.56	3.19	5.95	4.90	0.86
Myr-AKT3-NL	2.53	3.59	2.92	3.01	0.31
Myr-AKT3-T305A-NL	3.12	1.40	2.98	2.50	0.55
Myr-AKT3-S473A-NL	2.64	2.00	3.94	2.86	0.57
Myr-AKT3-T305A-S473A-NL	2.07	1.40	3.66	2.37	0.67
Myr-AKT3-C342S-NL	2.41	3.43	2.73	2.86	0.30

M

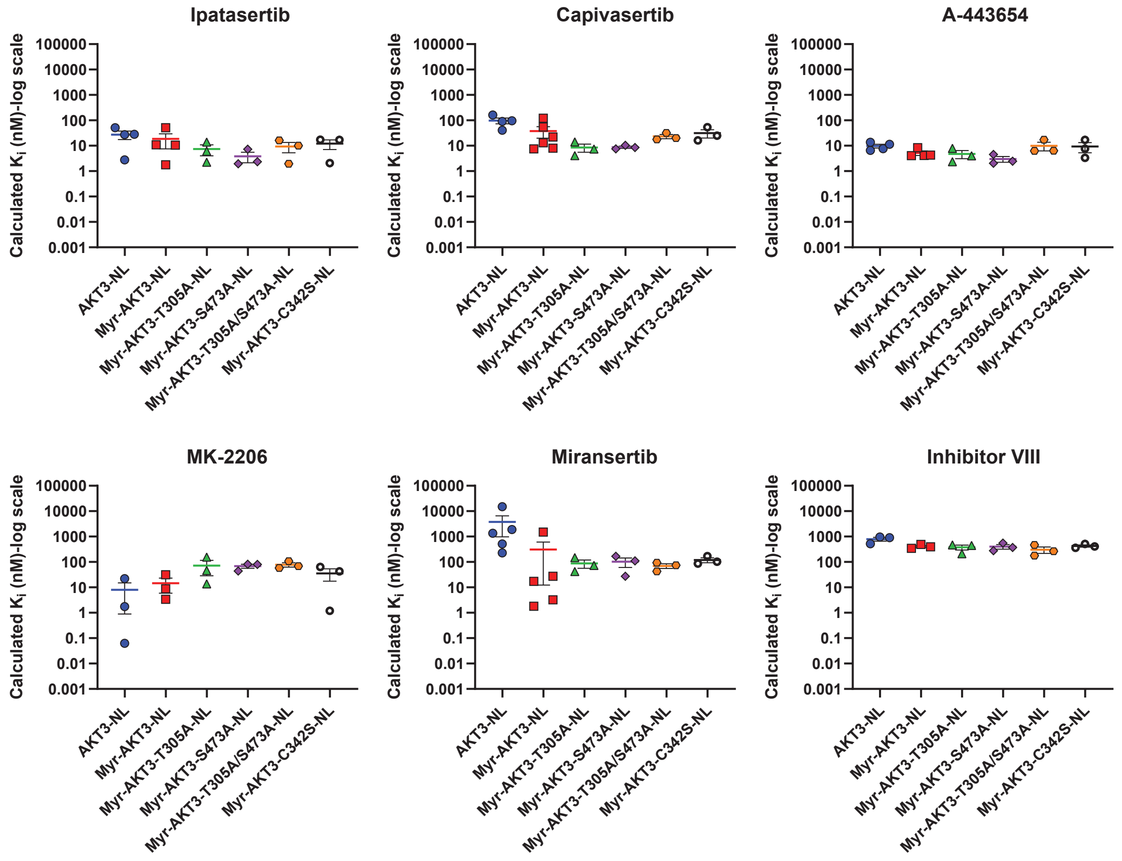

N

Calculated K_i Values

Ipatasertib	Replicate 1 (nM)	Replicate 2 (nM)	Replicate 3 (nM)	Replicate 4 (nM)	Average (nM)	Standard Error
AKT3-NL	27.42	2.76	51.66	28.01	27.46	9.98
Myr-AKT3-NL	10.88	1.77	10.56	51.27	18.62	11.09
Myr-AKT3-T305A-NL	6.83	2.50	15.58		8.31	3.85
Myr-AKT3-S473A-NL	2.69	2.21	8.26		4.39	1.94
Myr-AKT3-T305A-S473A-NL	10.073	1.94	16.16		9.39	4.12
Myr-AKT3-C342S-NL	17.12	2.07	16.95		12.05	4.99

Capivasertib	Replicate 1 (nM)	Replicate 2 (nM)	Replicate 3 (nM)	Replicate 4 (nM)	Replicate 5 (nM)	Replicate 6 (nM)	Average (nM)	Standard Error
AKT3-NL	90.97	161.60	96.87	40.46			97.47	24.84
Myr-AKT3-NL	22.20	13.08	8.04	54.95	7.51	120.85	37.77	18.12
Myr-AKT3-T305A-NL	4.53	15.81	8.25				9.53	3.32
Myr-AKT3-S473A-NL	8.52	9.68	11.86				10.02	1.00
Myr-AKT3-T305A-S473A-NL	32.06	17.70	20.24				23.33	4.42
Myr-AKT3-C342S-NL	53.73	24.81	16.24				31.59	11.34

A-443654	Replicate 1 (nM)	Replicate 2 (nM)	Replicate 3 (nM)	Replicate 4 (nM)	Average (nM)	Standard Error
AKT3-NL	6.58	7.66	13.67	11.32	9.80	1.63
Myr-AKT3-NL	4.07	4.14	8.41	4.27	5.22	1.06
Myr-AKT3-T305A-NL	4.48	2.62	8.87		5.32	1.85
Myr-AKT3-S473A-NL	2.79	2.33	5.16		3.43	0.88
Myr-AKT3-T305A-S473A-NL	6.34	17.25	6.28		9.96	3.65
Myr-AKT3-C342S-NL	16.94	3.35	7.68		9.32	4.01

MK-2206	Replicate 1 (nM)	Replicate 2 (nM)	Replicate 3 (nM)	Average (nM)	Standard Error
AKT3-NL	1.75	0.063	22.03	7.95	7.06
Myr-AKT3-NL	31.30	8.76	3.39	14.48	8.55
Myr-AKT3-T305A-NL	51.33	15.22	174.06	80.20	48.07
Myr-AKT3-S473A-NL	91.09	50.06	93.12	78.09	14.03
Myr-AKT3-T305A-S473A-NL	107.88	57.63	68.1	77.89	15.30
Myr-AKT3-C342S-NL	42.50	1.20	63.40	35.70	18.27

Miransertib	Replicate 1 (nM)	Replicate 2 (nM)	Replicate 3 (nM)	Replicate 4 (nM)	Replicate 5 (nM)	Average (nM)	Standard Error
AKT3-NL	1353.07	227.94	14966.68	512.54	1900.58	3792.16	2809.42
Myr-AKT3-NL	27.46	1.82	1492.62	3.26	17.19	308.47	296.08
Myr-AKT3-T305A-NL	46.78	82.01	165.76			98.18	35.28
Myr-AKT3-S473A-NL	126.07	31.07	191.25			116.13	46.51
Myr-AKT3-T305A-S473A-NL	42.84	92.68	74.45			70.00	14.56
Myr-AKT3-C342S-NL	86.15	102.60	169.10			119.28	25.36

Inhibitor VIII	Replicate 1 (nM)	Replicate 2 (nM)	Replicate 3 (nM)	Average (nM)	Standard Error
AKT3-NL	900.75	516.91	963.73	793.80	139.63
Myr-AKT3-NL	343.09	486.39	395.23	408.24	41.88
Myr-AKT3-T305A-NL	523.34	235.26	500.03	419.55	92.39
Myr-AKT3-S473A-NL	420.65	318.64	629.46	456.25	91.47
Myr-AKT3-T305A-S473A-NL	263.05	178.13	465.62	302.27	85.28
Myr-AKT3-C342S-NL	359.52	511.77	407.67	426.33	44.93

Figure S4- Replicate data associated with Figure 4. **(A)** Bioluminescent Imaging of HEK293T cells transfected with myristoylated NL-tagged constructs of AKT1, 2, and 3. Scale bar indicates 50 μm.**(B)** Signal build-up curves and fitted EC_{50} values using the K5 tracer on 293T cells transfected with AKT1-NL, AKT2-NL, and AKT3-NL mutants. **(C)** Replicate binding curves for AKT inhibitors on AKT1-NL, Myr-AKT1-NL, Myr-AKT1-T308A-NL, Myr-AKT1-S473A-NL, Myr-AKT1-T308A/S473A-NL, and Myr-AKT1-C344S-NL mutants. **(D)** Fitted apparent IC_{50} values for curves in (C). **(E)** Plotted calculated K_i values derived from EC_{50} and IC_{50} values in (B and Figure S1A) and (D). **(F)** K_i values plotted in (E). **(G)** Replicate binding curves for AKT inhibitors on AKT2-NL, Myr-AKT2-NL, Myr-AKT2-T309A-NL, Myr-AKT2-S474A-NL, Myr-AKT2-T309A/S474A-NL, and Myr-AKT2-C345S-NL mutants. **(H)** Fitted apparent IC_{50} values for curves in (G). **(I)** Plotted Calculated K_i values derived from EC_{50} and IC_{50} values in (B and Figure S1A) and (H). **(J)** K_i values plotted in (I). **(K)** Replicate binding curves for AKT inhibitors on AKT3-NL, Myr-AKT3-NL, Myr-AKT3-T305A-NL, Myr-AKT3-S473A-NL, Myr-AKT3-T305A/S473A-NL, and Myr-AKT3-C342S-NL mutants. **(L)** Fitted apparent IC_{50} values for curves in (J). **(M)** Plotted calculated K_i values derived from EC_{50} and IC_{50} values in (B and Figure S1A) and (L). **(N)** K_i values plotted in (M).

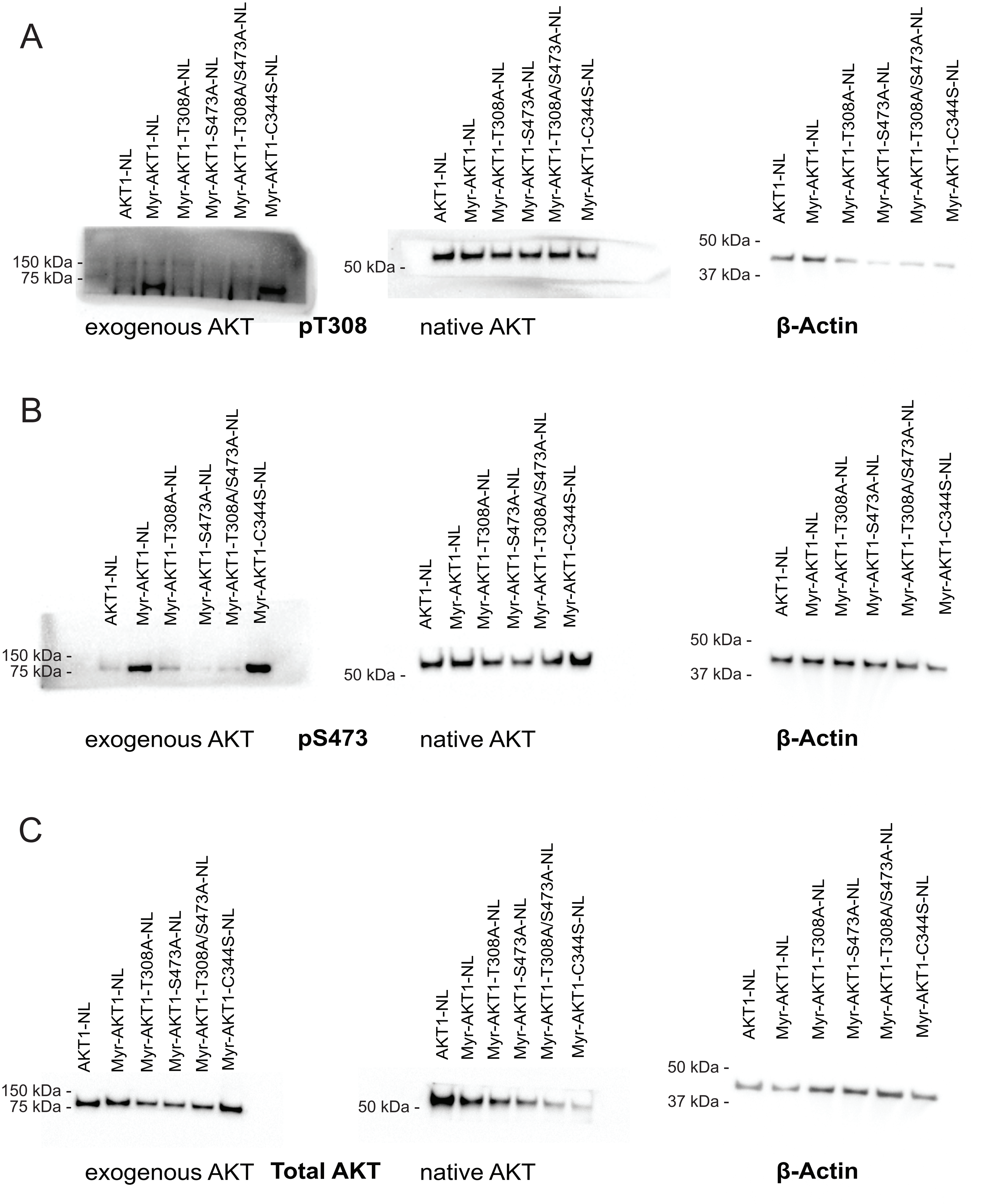

Figure S5- Uncropped Western Blots corresponding to Figure 4. Each set of blots are cut from the same SDS-PAGE gel. **(A)** pT308 (phosphorylated) and β-Actin. **(B)** pS473 (phosphorylated) and β-Actin. **(C)** Total AKT and β-Actin.

A

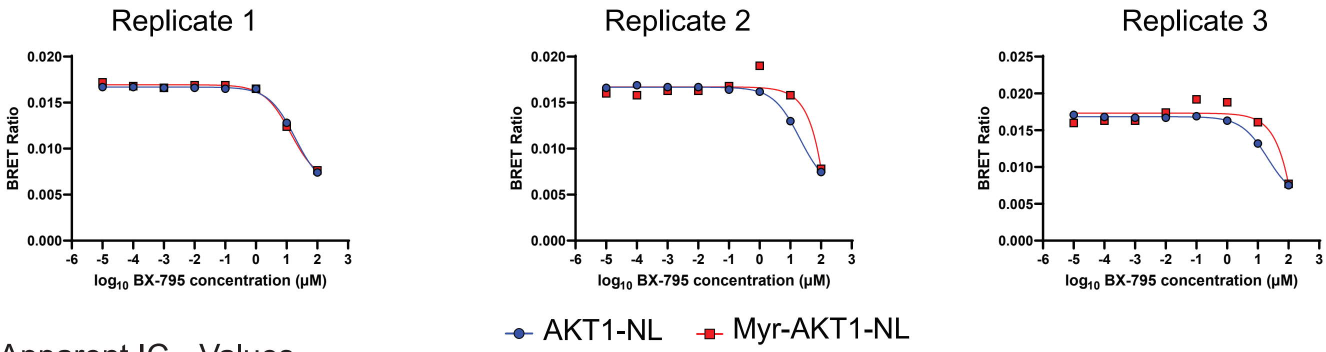

Apparent IC_{50} Values

BX-795	Replicate 1 (μM)	Replicate 2 (μM)	Replicate 3 (μM)	Average (μM)	Standard Error
AKT1-NL	19.06	20.18	20.83	20.02	0.52
Myr-AKT1-NL	14	N/A	1385	699	686

Calculated K_i Values

BX-795	Replicate 1 (nM)	Replicate 2 (nM)	Replicate 3 (nM)	Average (nM)	Standard Error
AKT1-NL	3417	3618	3735	3590	93
Myr-AKT1-NL	2117	N/A	212940	107528	105411

B **PDK1-NL**

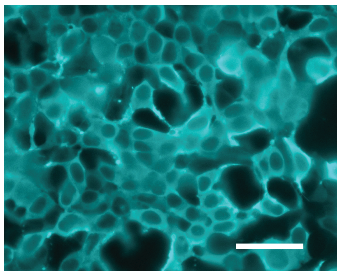

C **PDK1-NL**

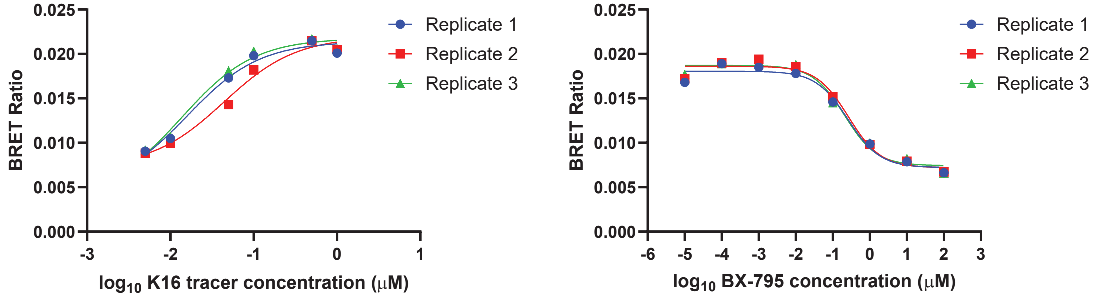

K16 tracer EC_{50} Values

Replicate 1 (μM)	Replicate 2 (μM)	Replicate 3 (μM)	Average (μM)	Standard Error
0.016	0.044	0.014	0.025	0.010

Apparent IC_{50} Values

Replicate 1 (μM)	Replicate 2 (μM)	Replicate 3 (μM)	Average (μM)	Standard Error
0.257	0.266	0.209	0.244	0.018

Calculated K_i Values

Replicate 1 (nM)	Replicate 2 (nM)	Replicate 3 (nM)	Average (nM)	Standard Error
6.17	6.38	5.01	5.85	0.43

D K5 tracer EC_{50} Values

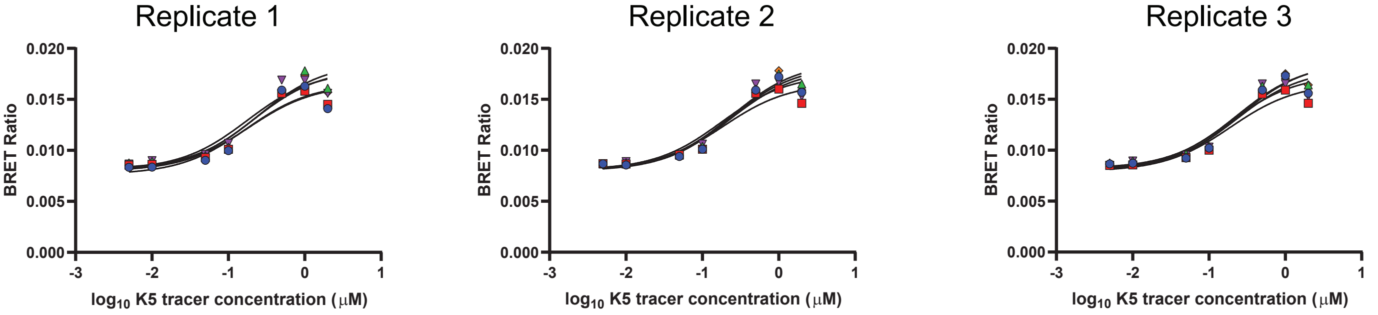

K5 tracer	Replicate 1 (μM)	Replicate 2 (μM)	Replicate 3 (μM)	Average (μM)	Standard Error
AKT1-NL	0.171	0.225	0.224	0.207	0.018
AKT1-NL + BX-795 (1 μM)	0.184	0.187	0.191	0.187	0.002
Myr-AKT1-NL	0.244	0.242	0.232	0.240	0.004
Myr-AKT1-NL+BX-795	0.176	0.177	0.185	0.180	0.003
Myr-AKT1 +PDK1 (0.5 μg/mL) + BX-795 (1 μM)	0.213	0.231	0.250	0.231	0.011

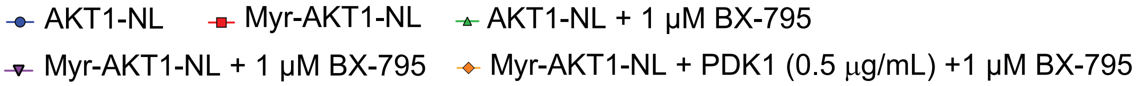

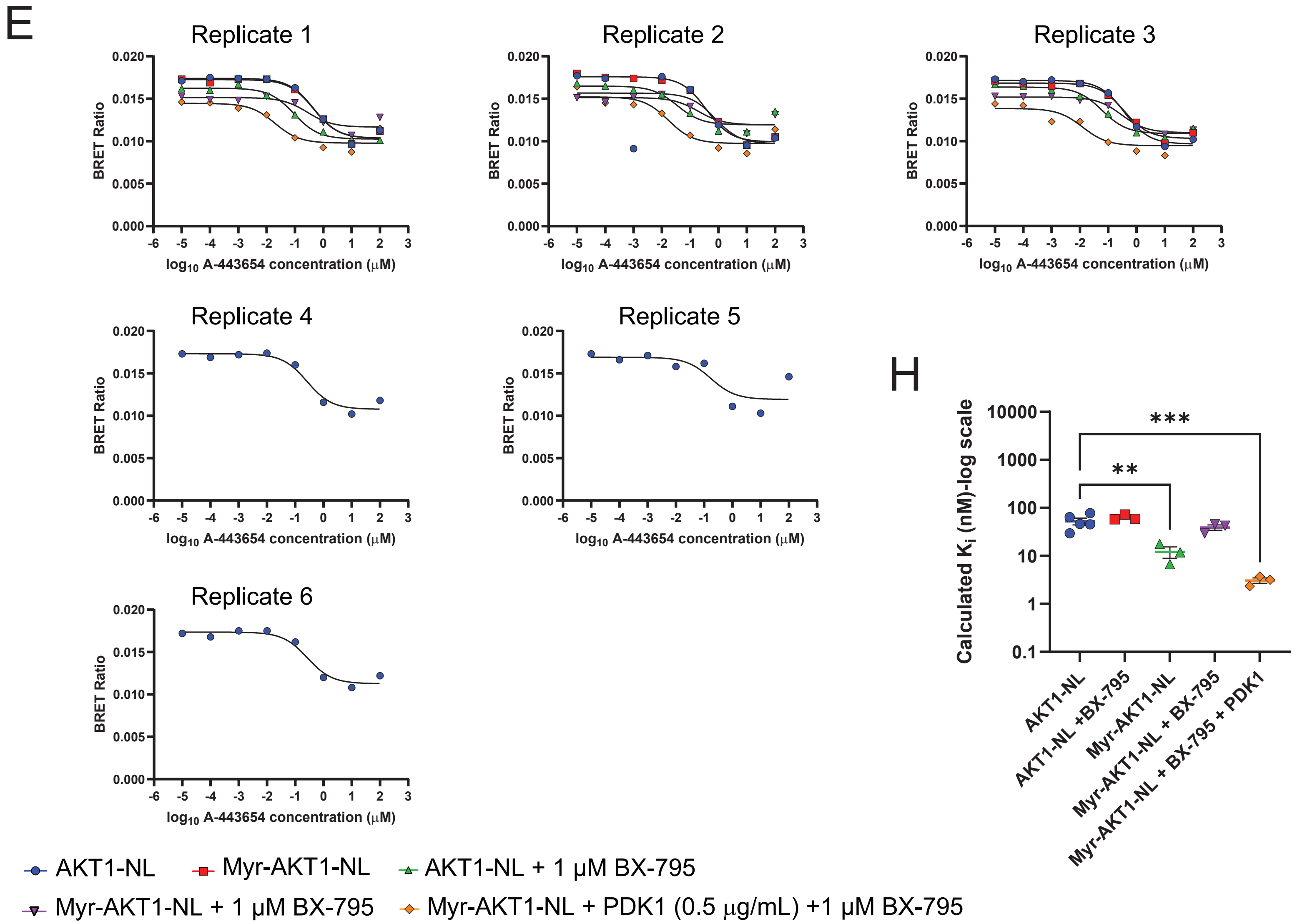

F Apparent IC_{50} Values

A-443654	Replicate 1 (μM)	Replicate 2 (μM)	Replicate 3 (μM)	Replicate 4 (μM)	Replicate 5 (μM)	Replicate 6 (μM)	Average (μM)	Standard Error
AKT1-NL	0.453	0.767	0.374	0.265	0.172	0.270	0.384	0.086
AKT1-NL + BX-795 (1 μM)	0.459	0.372	0.364					0.030
Myr-AKT1-NL	0.092	0.035	0.061					0.017
Myr-AKT1-NL+BX-795	0.278	0.192	0.298					0.033
Myr-AKT1 +PDK1 (0.5 μg/mL) + BX-795 (1 μM)	0.020	0.017	0.012					0.002

G Calculated K_i Values

A-443654	Replicate 1 (nM)	Replicate 2 (nM)	Replicate 3 (nM)	Replicate 4 (nM)	Replicate 5 (nM)	Replicate 6 (nM)	Average (nM)	Standard Error
AKT1-NL	77.58	131.21	64.07	45.31	29.48	46.24	65.65	14.77
AKT1-NL + BX-795 (1 μM)	72.34	58.69	57.36				62.80	4.79
Myr-AKT1-NL	17.78	6.70	11.83				12.10	3.20
Myr-AKT1-NL+BX-795	42.33	29.16	45.36				38.95	4.97
Myr-AKT1 +PDK1 (0.5 μg/mL) + BX-795 (1 μM)	3.69	3.18	2.34				3.07	0.40

Figure S6- Data associated with Figure 5B and C. **(A)** Dose-response curves of BX-795 on AKT1-NL and Myr-AKT1-NL. Tables below indicate fitted apparent IC_{50} and K_i values. K_i values were calculated using K5 EC_{50} values from Figure S1A and Figure S4B. **(B)** Bioluminescent imaging of PDK1-NL in HEK293T. Scale bar indicates 50 μm. **(C)** EC_{50} buildup curves for the K16 tracer, dose-response curves for BX-795, and fitted values of K16 EC_{50}, apparent BX-795 IC_{50} and calculated BX-795 K_i values using the PDK1-NL construct. **(D)** EC_{50} buildup curves for the K5 tracer and fitted values for AKT constructs in the presence and absence of BX-795. **(E)** Dose-response curves of A-443654 in the presence and absence of 1 μM BX-795. **(F)** Fitted apparent IC_{50} values from curves in (E). **(G)** Calculated K_i values using IC_{50}values from (F) and EC_{50} values from (D). **(H)** K_i values plotted from (G). Data were analyzed by one-way ANOVA with Dunnett's multiple comparison test (AKT1-NL vs. AKT1-NL + 1 μM BX-795 adjusted P=0.6383, AKT1-NL vs. Myr-AKT1-NL adjusted P=0.0022, AKT1-NL vs. Myr-AKT1-NL + 1 μM BX-795 adjusted P=0.4078, AKT1-NL vs. Myr-AKT1-NL + PDK1 (0.5 μg/mL) 1 μM BX-795 adjusted P=0.0004).

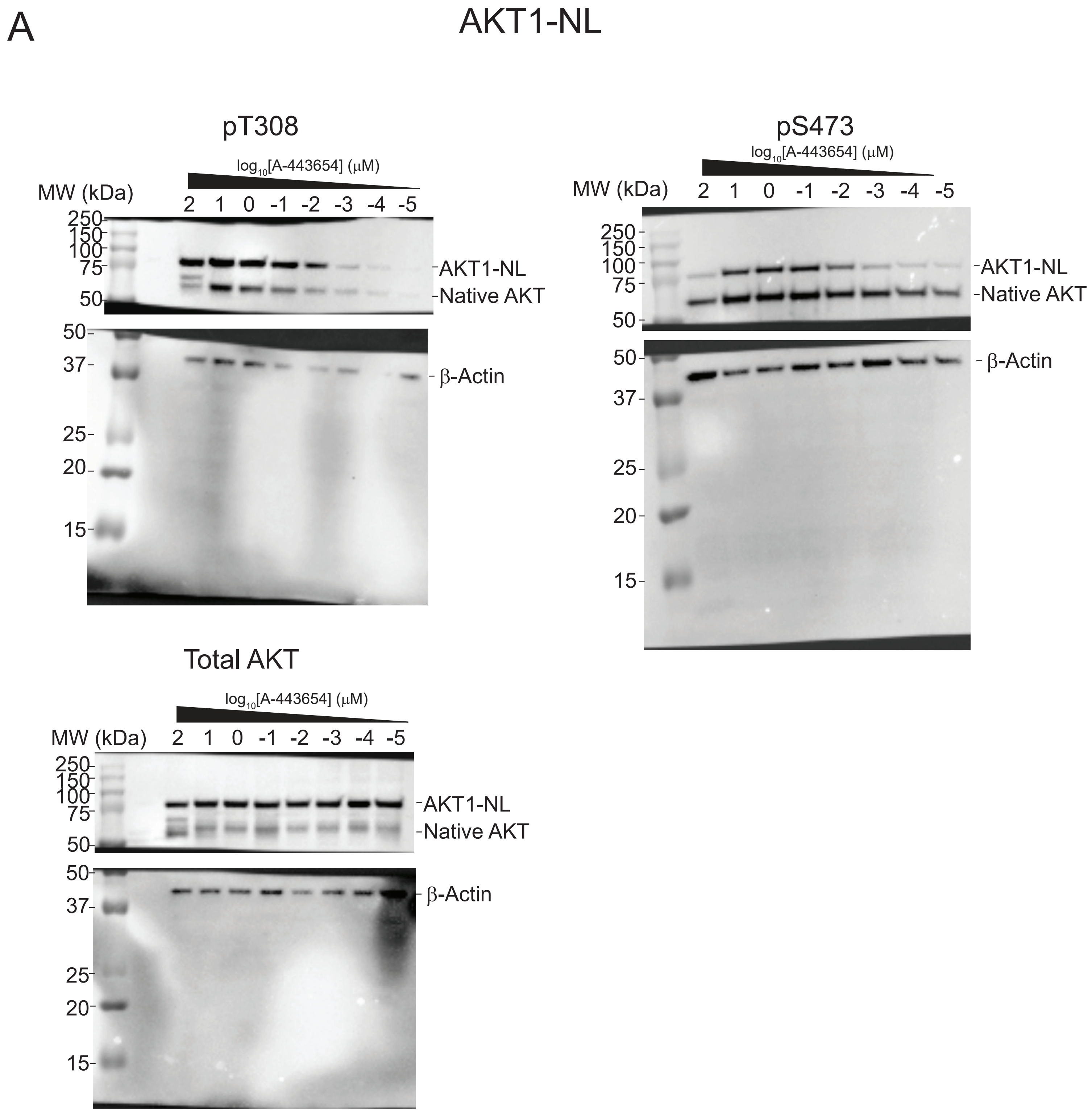
A
AKT1-NL
pT308
log10[A-443654] (μM)
MW (kDa)
2 1 0 -1 -2 -3 -4 -5
250
150
100
75
50
AKT1-NL
Native AKT
50
37
25
20
15
β-Actin
pS473
log10[A-443654] (μM)
MW (kDa)
2 1 0 -1 -2 -3 -4 -5
250
150
100
75
50
AKT1-NL
Native AKT
50
37
25
20
15
β-Actin
Total AKT
log10[A-443654] (μM)
MW (kDa)
2 1 0 -1 -2 -3 -4 -5
250
150
100
75
50
AKT1-NL
Native AKT
50
37
25
20
15
β-Actin

B

AKT1-NL + 1 μM BX-795

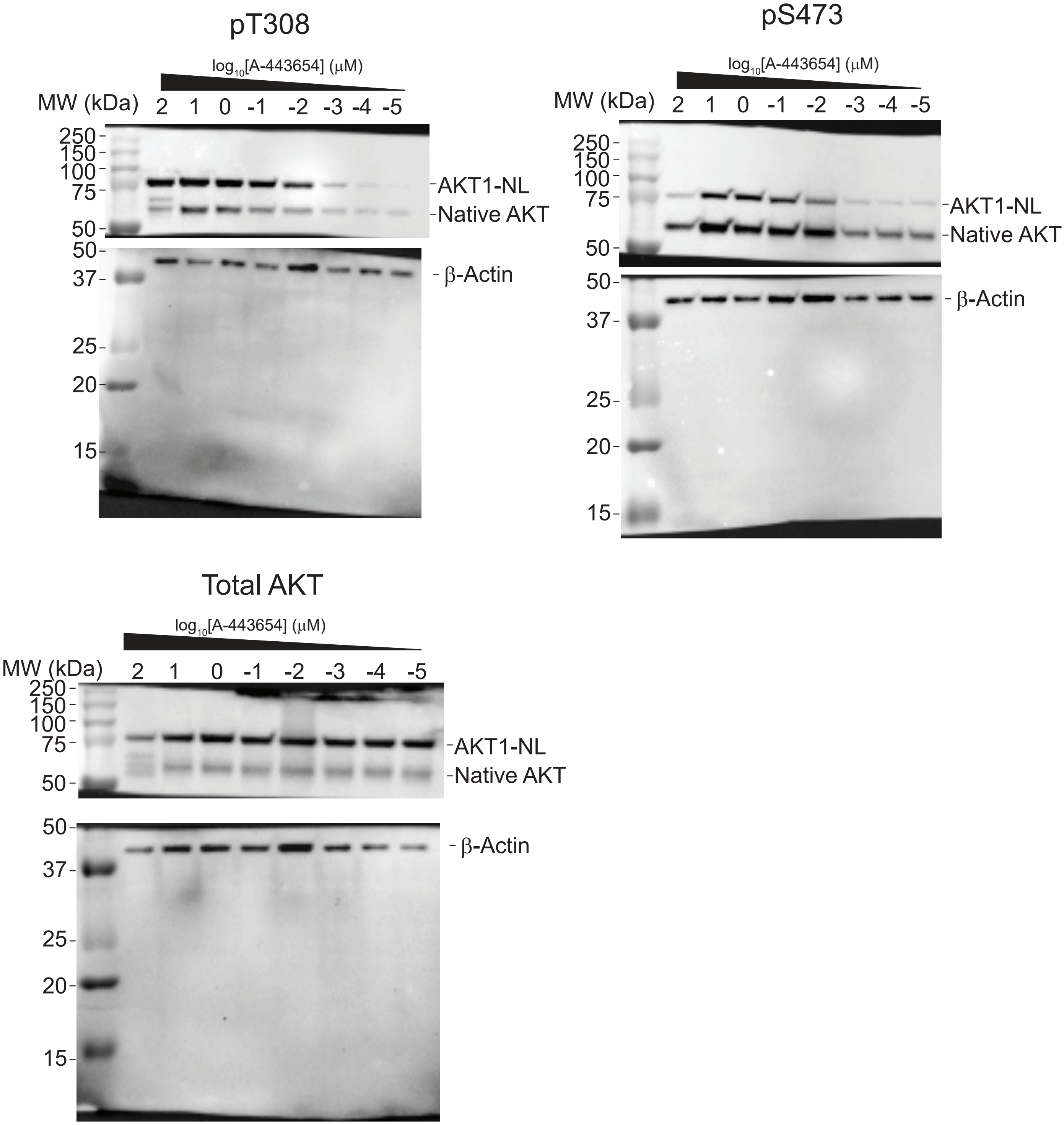

C

Myr-AKT1-NL

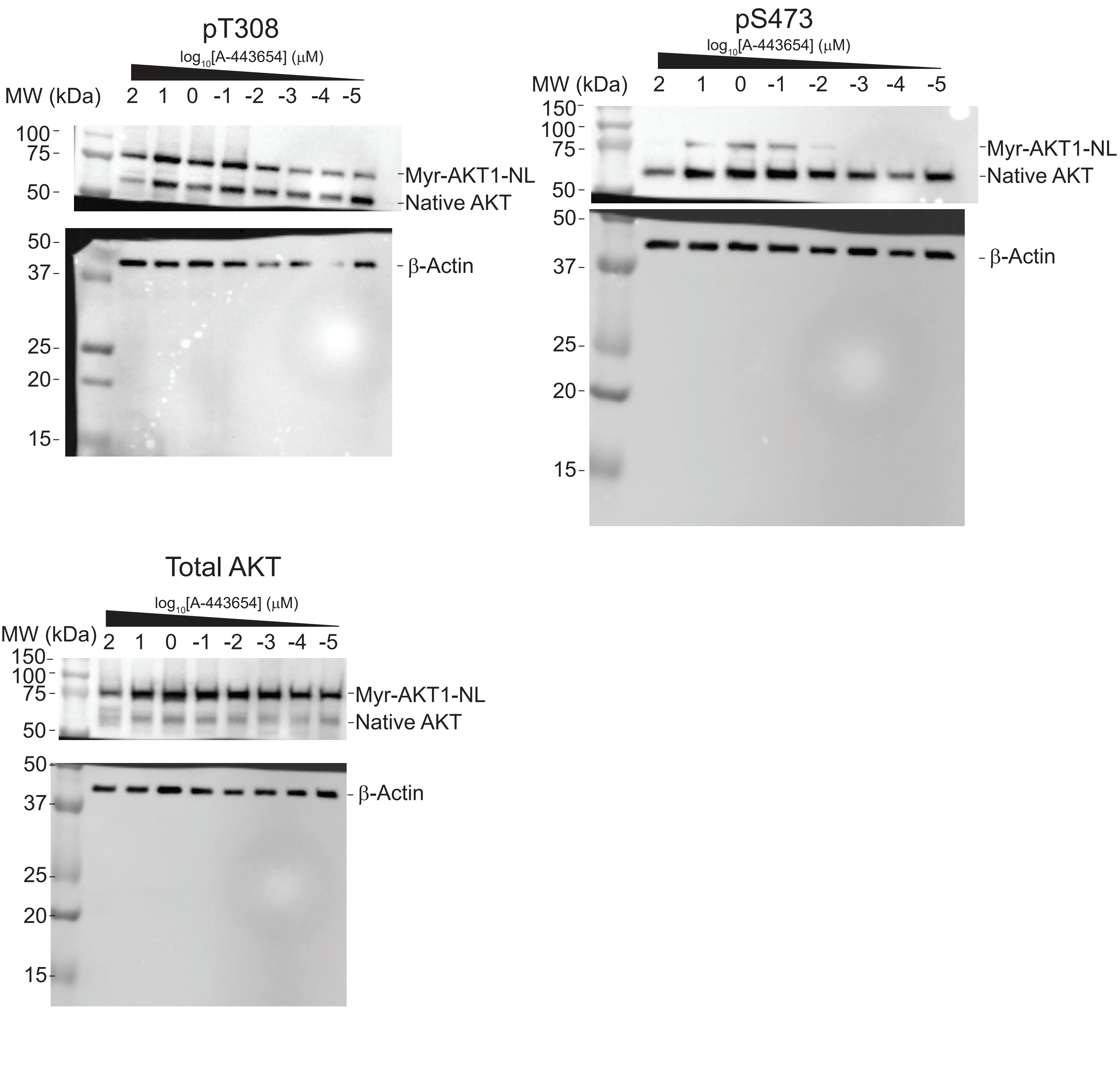
pT308
log10[A-443654] (μM)
MW (kDa) 2 1 0 -1 -2 -3 -4 -5
100
75
50
Myr-AKT1-NL
Native AKT
50
37
25
20
15
β-Actin
pS473
log10[A-443654] (μM)
MW (kDa) 2 1 0 -1 -2 -3 -4 -5
150
100
75
50
Myr-AKT1-NL
Native AKT
50
37
25
20
15
β-Actin
Total AKT
log10[A-443654] (μM)
MW (kDa) 2 1 0 -1 -2 -3 -4 -5
150
100
75
50
Myr-AKT1-NL
Native AKT
50
37
25
20
15
β-Actin

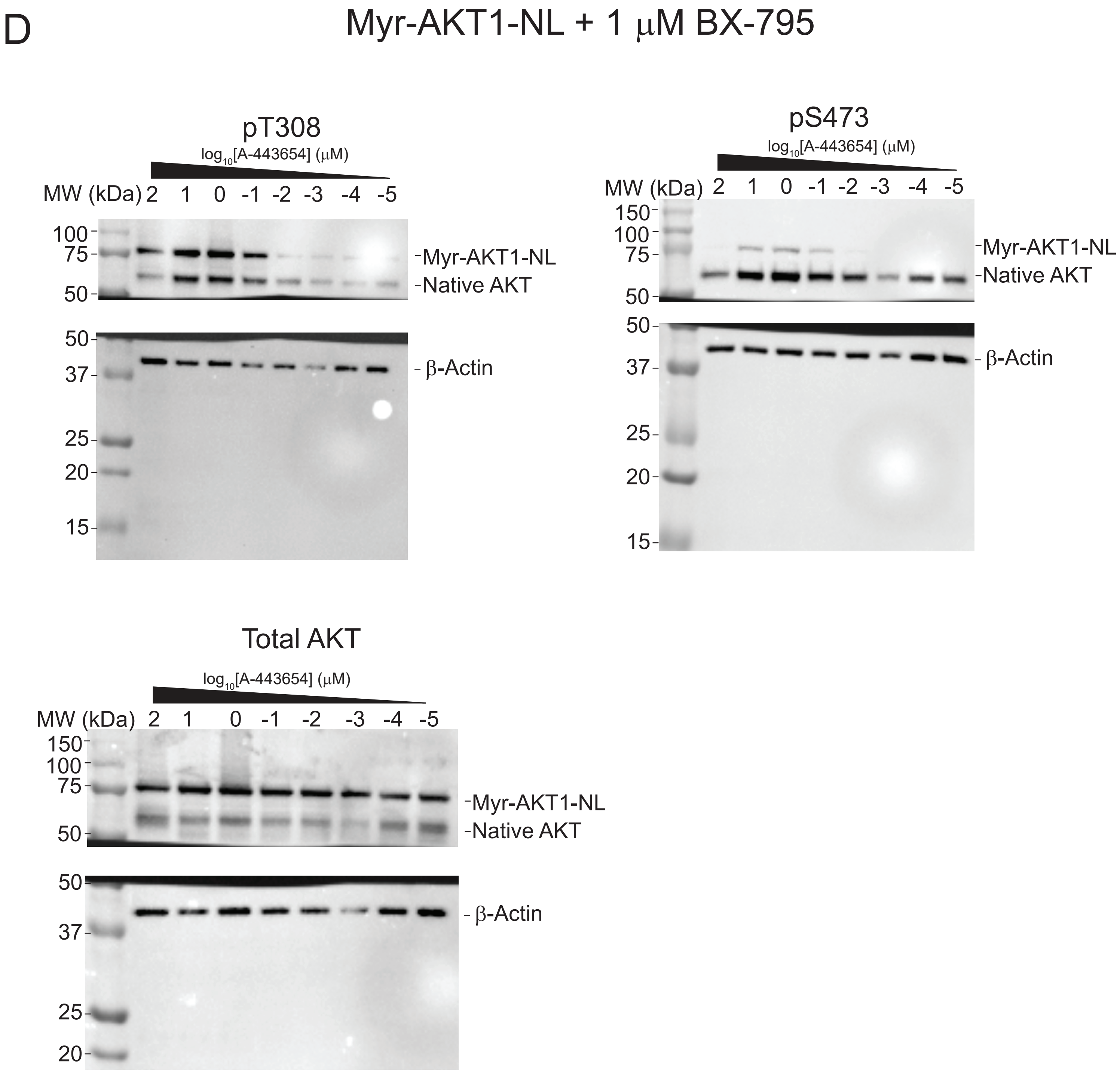

Figure S7- Uncropped Western blots associated with Figure 5D and E. Blots indicate T308 and S473 phosphorylation, and Total AKT in the presence of a A-443654 dose-response curves. Each blot has an accompanying β-actin blot associated with the same SDS-PAGE gel. **(A)** Over-expressed AKT1-NL. **(B)** Over-expressed AKT1-NL in the presence of 1 μM BX-795. **(C)** Over-expressed Myr-AKT1-NL. **(D)** Over-expressed Myr-AKT1-NL in the presence of 1 μM BX-795.

A

MDA-MB-231

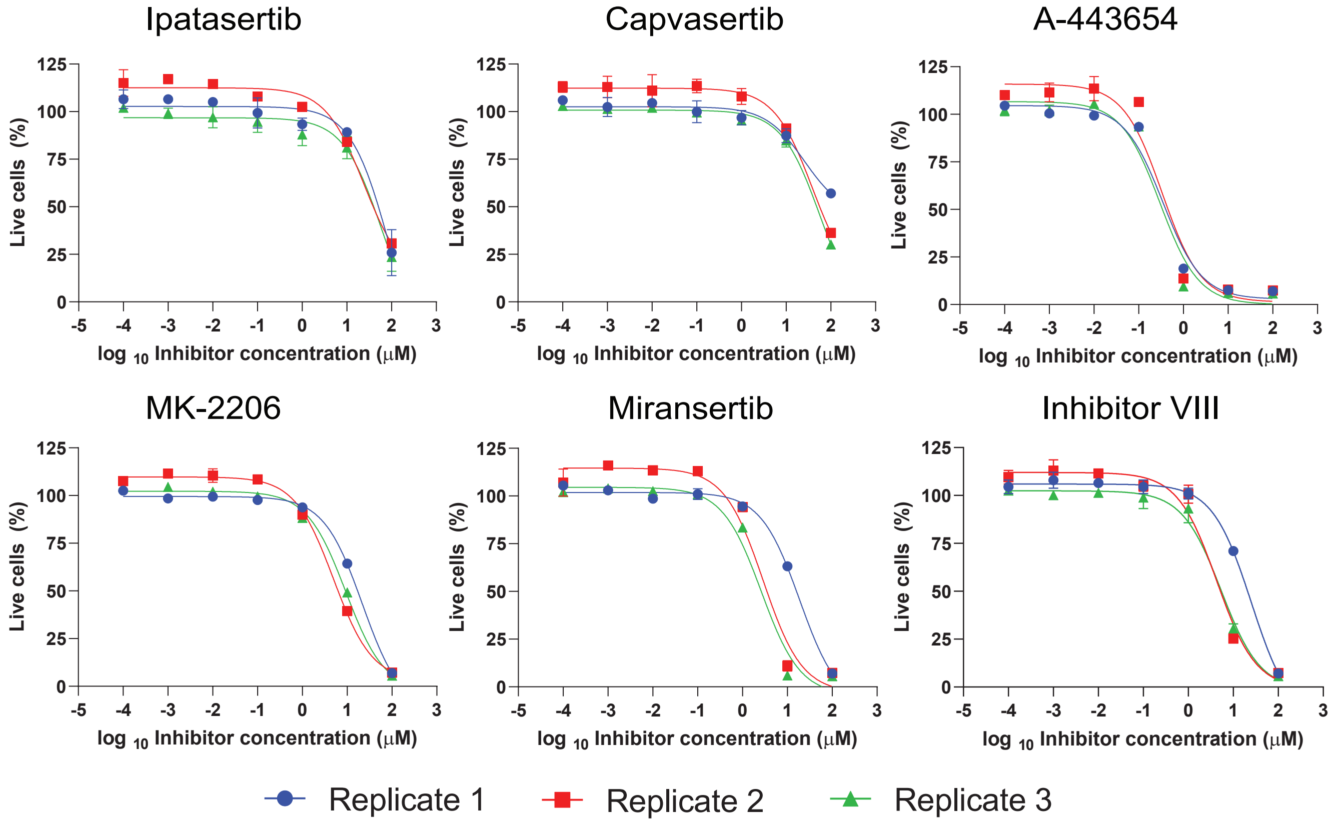

IC$_{50}$ values

MDA-MB-231					
	Replicate 1 (μM)	Replicate 2 (μM)	Replicate 3 (μM)	Average (μM)	Standard Error
Ipatasertib	90.31	24.03	60.19	58.18	19.16
Capivasertib	25.71	39.38	57.57	40.89	9.23
A-443654	0.35	0.34	0.30	0.33	0.01
MK-2206	21.86	4.96	9.27	12.03	5.07
Miransertib	18.79	2.89	2.62	8.10	5.35
Inhibitor VIII	25.28	4.28	5.37	11.64	6.83

B

MDA-MB-468

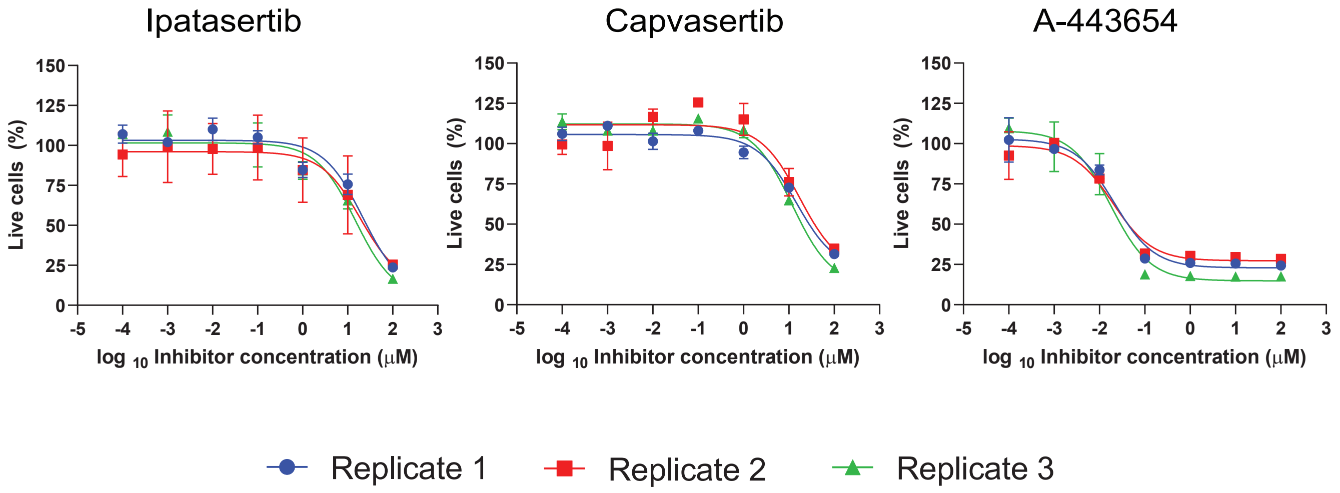

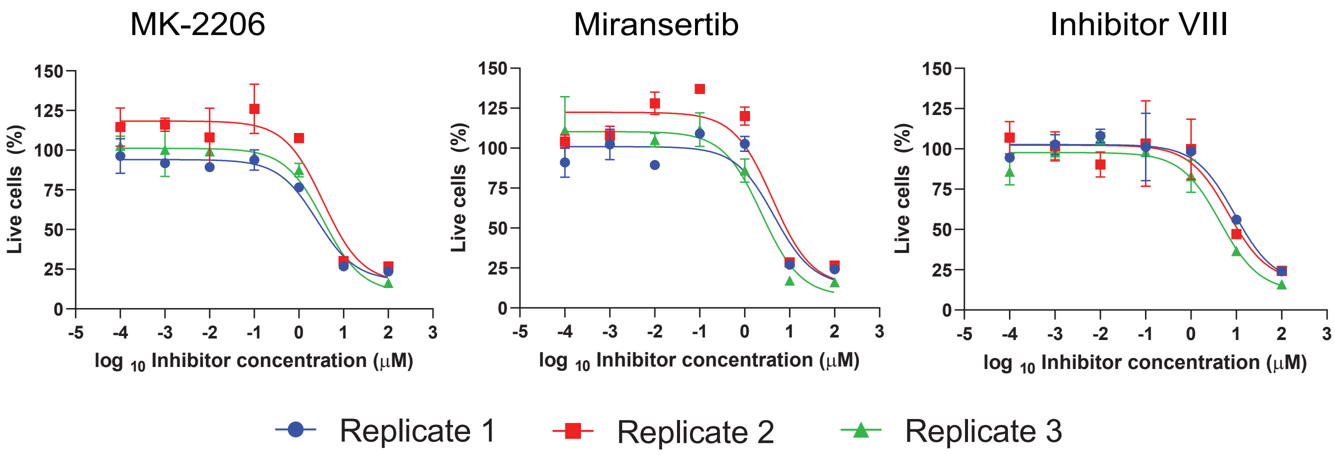

IC$_{50}$ values

MDA-MB-468					
	Replicate 1 (µM)	Replicate 2 (µM)	Replicate 3 (µM)	Average (µM)	Standard Error
Ipatasertib	21.50	19.33	14.80	18.54	1.97
Capivasertib	14.61	17.28	11.84	14.58	1.57
A-443654	0.022	0.020	0.018	0.020	0.001
MK-2206	2.53	3.53	3.53	3.20	0.33
Miransertib	4.56	4.19	2.39	3.71	0.67
Inhibitor VIII	9.21	6.71	4.41	6.78	1.39

Figure S8- Replicate cell viability curves and fitted IC$_{50}$ values for MDA-MB-231 **(A)** and MDA-MB-468 **(B)** cell lines. Data correspond to Figure 6A and B.

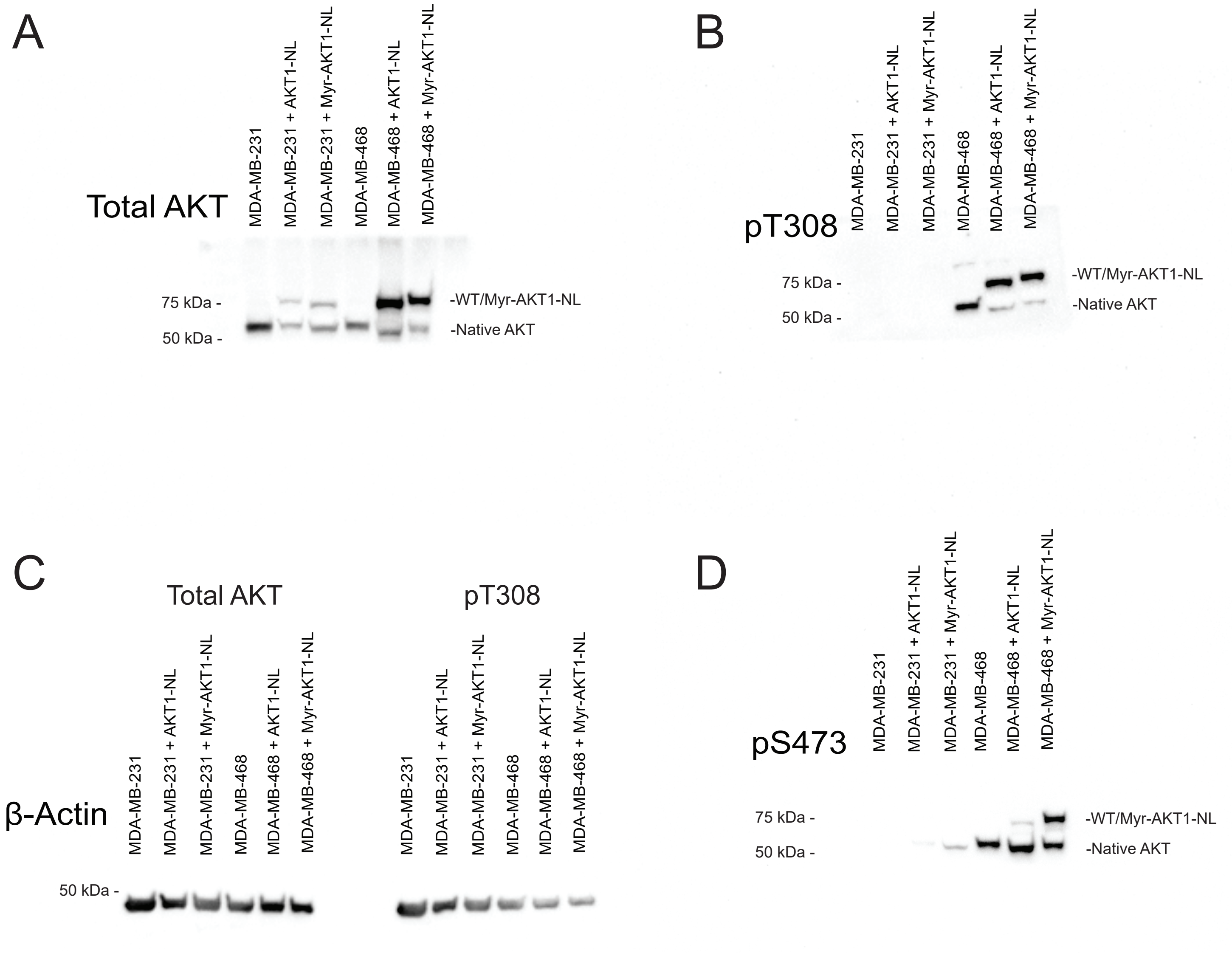

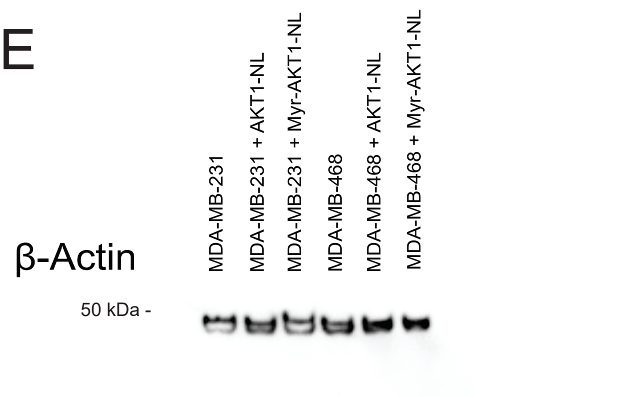

Figure S9- Uncropped Western blots for Figure 6C. **(A)** Total AKT. **(B)** T308 phosphorylaton. **(C)** β-Actin blots corresponding to Total AKT and T308 blots. **(D)**. S473 phosphorylation. **(E)** β-Actin blot corresponding to the S473 blot.

A

AKT1-NL

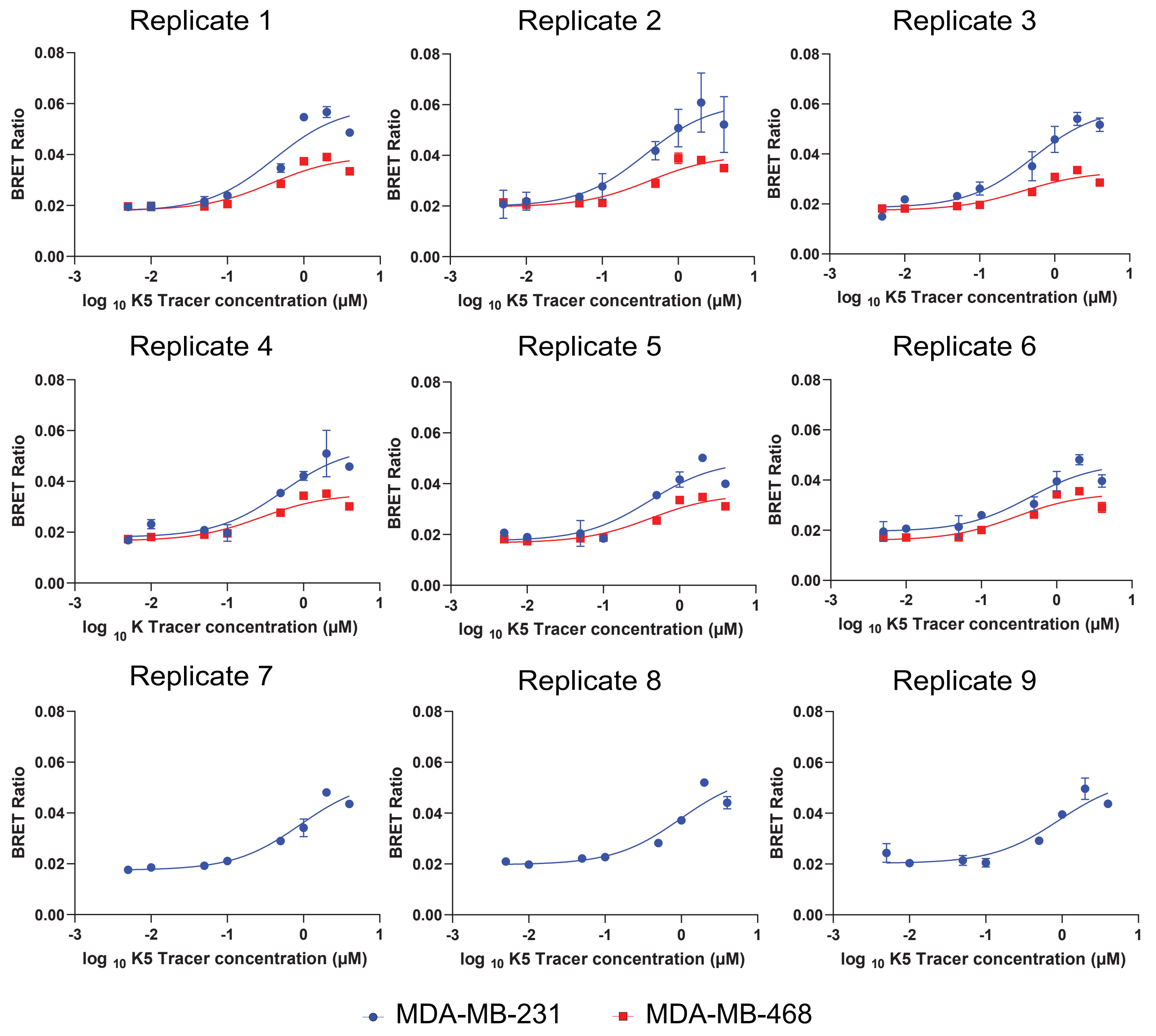

AKT1-NL	Replicate 1 (μM)	Replicate 2 (μM)	Replicate 3 (μM)	Replicate 4 (μM)	Replicate 5 (μM)	Replicate 6 (μM)	Replicate 7 (μM)	Replicate 8(μM)	Replicate 9 (μM)	Average (μM)	Standard Error
MDA-MB-231	0.41	0.37	0.52	0.53	0.41	0.44	0.91	0.97	0.91	0.61	0.08
MDA-MB-468	0.38	0.41	0.38	0.28	0.28	0.29				0.33	0.03

Myr-AKT1-NL

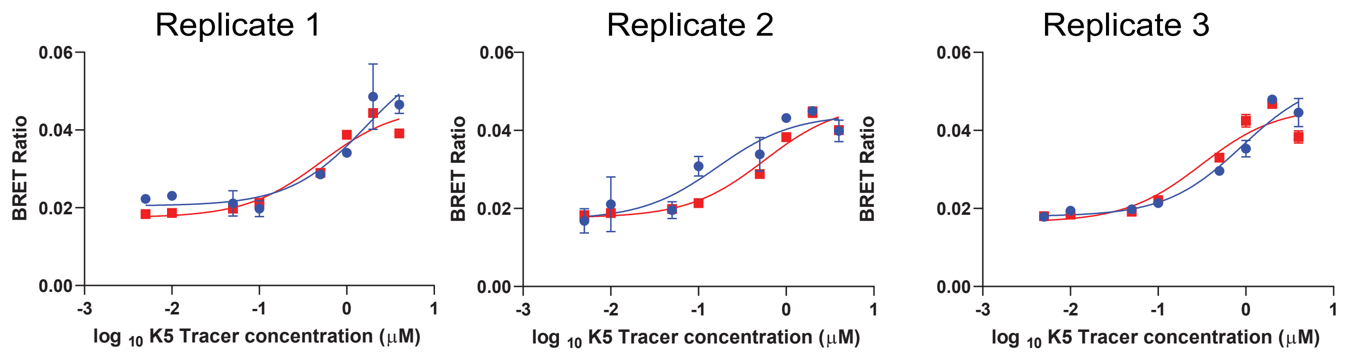

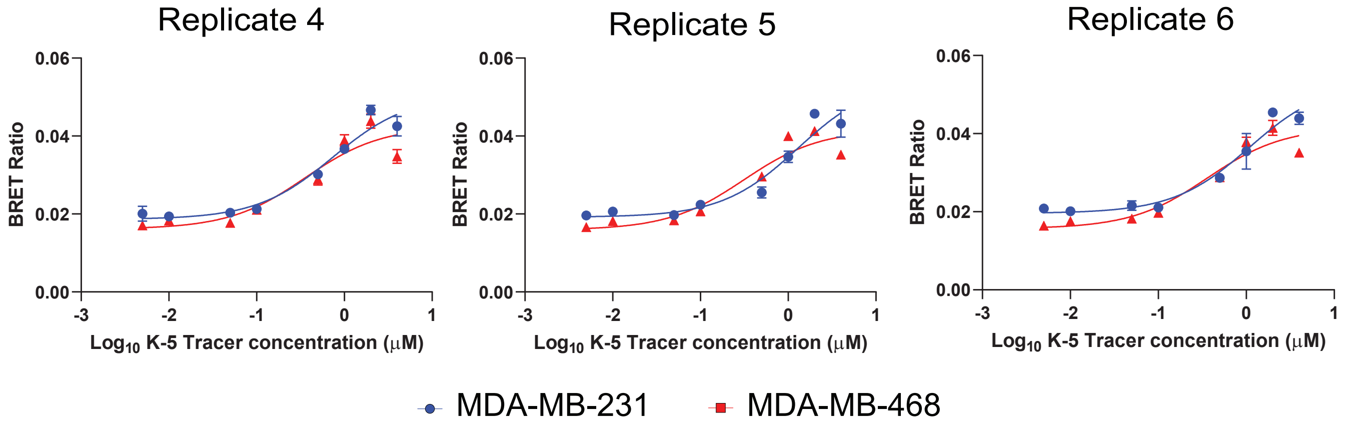

Myr-AKT1-NL	Replicate 1 (μM)	Replicate 2 (μM)	Replicate 3 (μM)	Replicate 4 (μM)	Replicate 5 (μM)	Replicate 6 (μM)	Average (μM)	Standard Error
MDA-MB-231	1.63	0.16	0.90	0.74	1.29	1.09	0.97	0.20
MDA-MB-468	0.51	0.57	0.31	0.36	0.31	0.36	0.40	0.04

B

MDA-MB-231 AKT1-NL

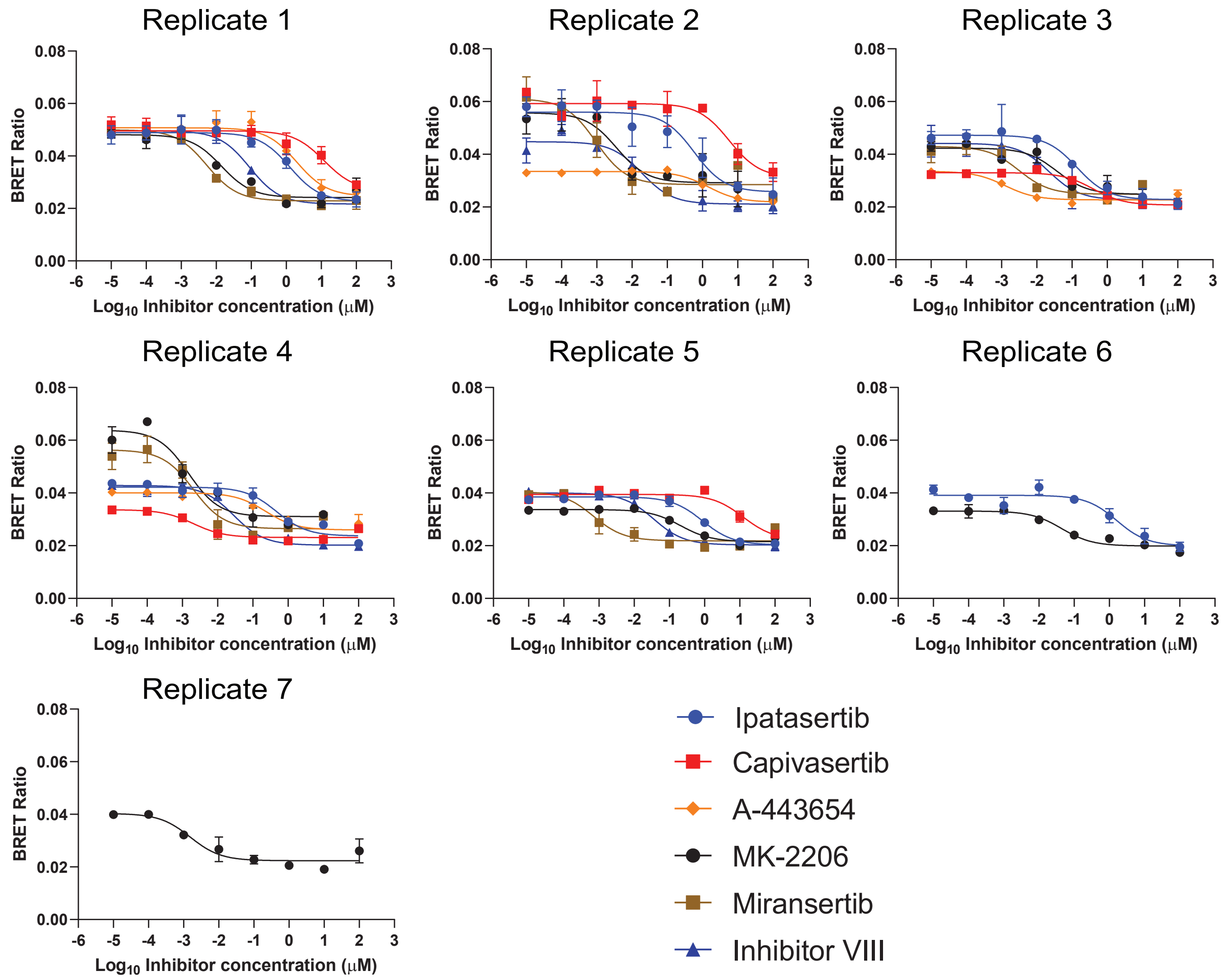

Apparent IC_{50} Values

MDA-MB-231									
AKT1-NL	Replicate 1 (μM)	Replicate 2 (μM)	Replicate 3 (μM)	Replicate 4 (μM)	Replicate 5 (μM)	Replicate 6 (μM)	Replicate 7 (μM)	Average (μM)	Standard Error
Ipatasertib	1.26	0.58	0.14	0.47	0.88	1.63		0.83	0.22
Capivasertib	12.050	6.422	0.400	0.002	12.570			6.289	2.710
A-443654	1.9910	1.4560	0.0013	0.2323				0.9201	0.4788
MK-2206	0.0141	0.0036	0.0374	0.0008	0.2076	0.0441	0.0015	0.0442	0.0280
Miransertib	0.0054	0.0009	0.0031	0.0016	0.0007			0.0024	0.0009
Inhibitor VIII	0.0901	0.0189	0.0223	0.0287	0.0358			0.0391	0.0131

Calculated K_i Values

MDA-MB-231									
AKT1-NL	Replicate 1 (nM)	Replicate 2 (nM)	Replicate 3 (nM)	Replicate 4 (nM)	Replicate 5 (nM)	Replicate 6 (nM)	Replicate 7(nM	Average (nM)	Standard Error
Ipatasertib	475.36	219.52	52.29	177.65	330.38	616.20		311.90	84.38
Capivasertib	4549.74	2424.77	150.92	0.77	4746.08			2374.45	1023.17
A-443654	751.75	549.74	0.47	87.71				347.42	180.79
MK-2206	5.31	1.37	14.13	0.30	78.38	16.65	0.58	16.68	10.58
Miransertib	2.05	0.36	1.17	0.62	0.26			0.89	0.33
Inhibitor VIII	34.02	7.12	8.40	10.83	13.50			14.77	4.93

C

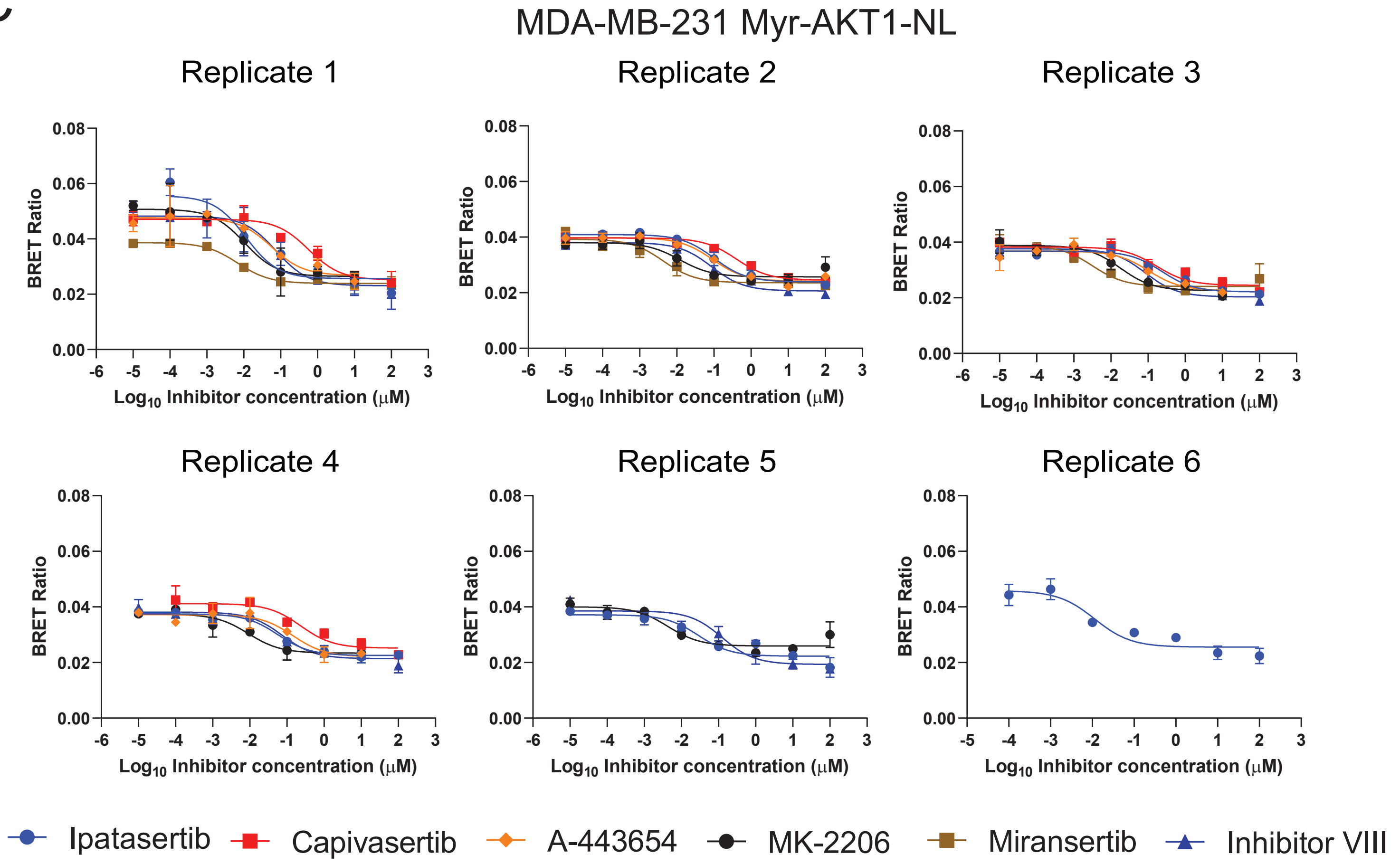

Apparent IC_{50} Values

MDA-MB-231								
Myr-AKT1-NL	Replicate 1 (μM)	Replicate 2 (μM)	Replicate 3 (μM)	Replicate 4 (μM)	Replicate 5 (μM)	Replicate 6 (μM)	Average (μM)	Standard Error
Ipatasertib	0.0114	0.1013	0.2363	0.0573	0.0290	0.0114	0.0744	0.0352
Capivasertib	0.5733	0.4038	0.1711	0.2348			0.3458	0.0904
A-443654	0.0557	0.0920	0.1036	0.1454			0.0992	0.0185
MK-2206	0.0105	0.0119	0.0178	0.0092	0.0043		0.0108	0.0022
Miransertib	0.0068	0.0043	0.0032				0.0048	0.0011
Inhibitor VIII	0.0757	0.0872	0.0845	0.0755	0.1263		0.0898	0.0094

Calculated K_i Values

MDA-MB-231								
Myr-AKT1-NL	Replicate 1 (nM)	Replicate 2 (nM)	Replicate 3 (nM)	Replicate 4 (nM)	Replicate 5 (nM)	Replicate 6(nM)	Average (nM)	Standard Error
Ipatasertib	5.59	49.83	116.23	28.18	14.24	5.58	36.61	17.33
Capivasertib	282.00	198.63	84.16	115.50			188.26	57.35
A-443654	27.41	45.25	50.96	71.52			41.21	7.09
MK-2206	5.18	5.87	8.77	4.51	2.12		5.29	1.07
Miransertib	3.35	2.11	1.59				2.73	0.62
Inhibitor VIII	37.22	42.90	41.55	37.12	62.13		44.18	4.63

D

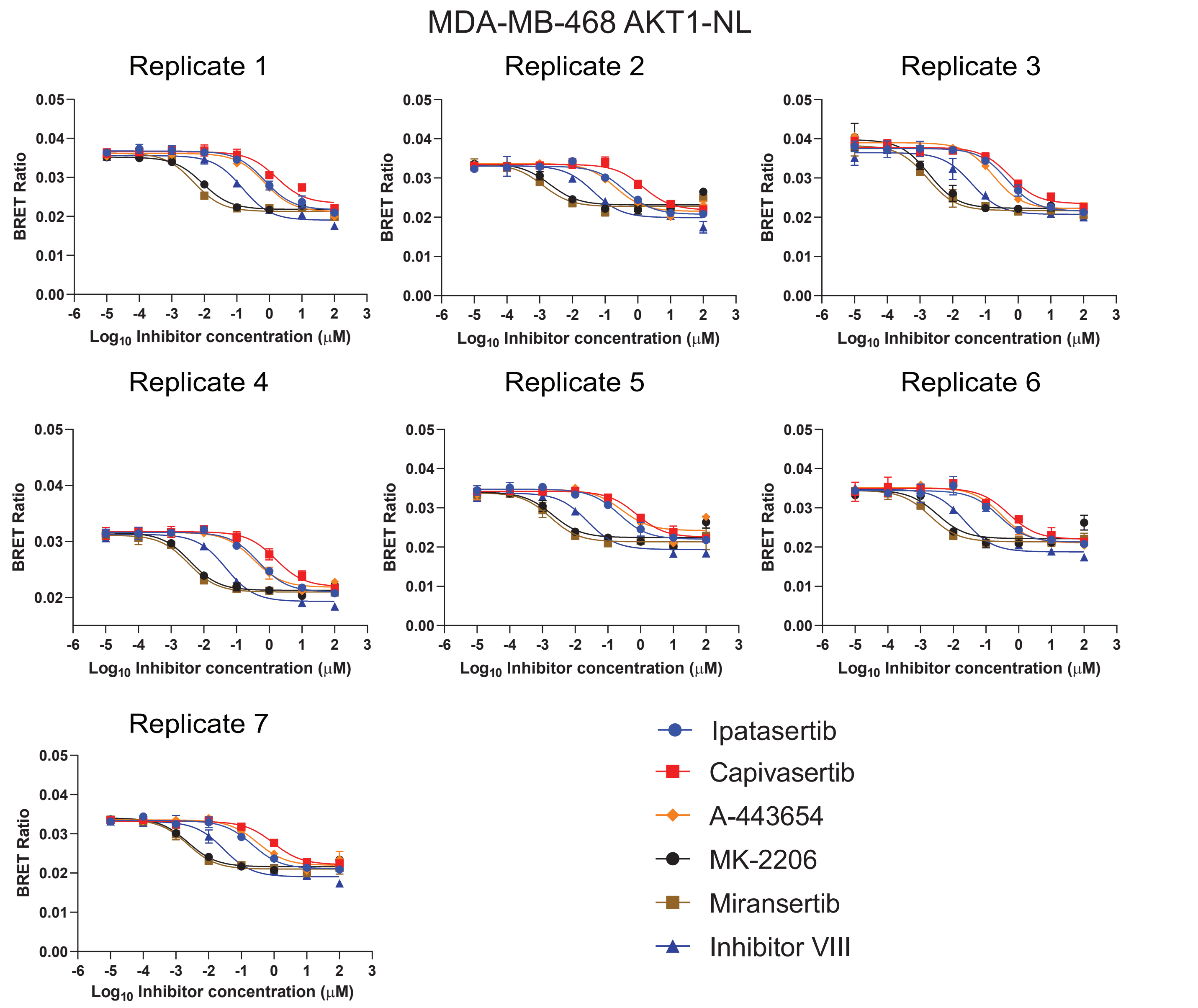

Apparent IC_{50} Values

MDA-MB-468 AKT1-NL	Replicate 1 (μM)	Replicate 2 (μM)	Replicate 3 (μM)	Replicate 4 (μM)	Replicate 5 (μM)	Replicate 6 (μM)	Replicate 7 (μM)	Average (μM)	Standard Error
Ipatasertib	0.714	0.400	0.468	0.478	0.221	0.259	0.216	0.394	0.068
Capivasertib	1.657	1.456	0.550	1.563	0.751	0.489	0.949	1.059	0.186
A-443654	0.707	0.208	0.176	0.339	0.267	0.305	0.310	0.330	0.067
MK-2206	0.009	0.002	0.002	0.004	0.002	0.003	0.002	0.003	0.001
Miransertib	0.005	0.001	0.002	0.003	0.002	0.002	0.002	0.002	0.0005
Inhibitor VIII	0.146	0.044	0.038	0.047	0.024	0.022	0.029	0.050	0.016

Calculated K_i Values

MDA-MB-468 AKT1-NL	Replicate 1 (nM)	Replicate 2 (nM)	Replicate 3 (nM)	Replicate 4 (nM)	Replicate 5 (nM)	Replicate 6(nM)	Replicate 7(nM	Average (nM)	Standard Error
Ipatasertib	187.78	105.11	122.96	125.72	58.04	68.13	56.69	103.49	17.92
Capivasertib	435.73	382.87	144.68	411.01	197.35	128.62	249.50	278.54	49.03
A-443654	185.94	54.59	46.15	89.14	70.08	80.12	81.54	86.80	17.51
MK-2206	2.31	0.54	0.49	1.02	0.54	0.86	0.61	0.91	0.25
Miransertib	1.24	0.33	0.44	0.90	0.46	0.52	0.60	0.64	0.12
Inhibitor VIII	38.50	11.60	9.97	12.29	6.21	5.74	7.53	13.12	4.34

E

MDA-MB-468 Myr-AKT1-NL

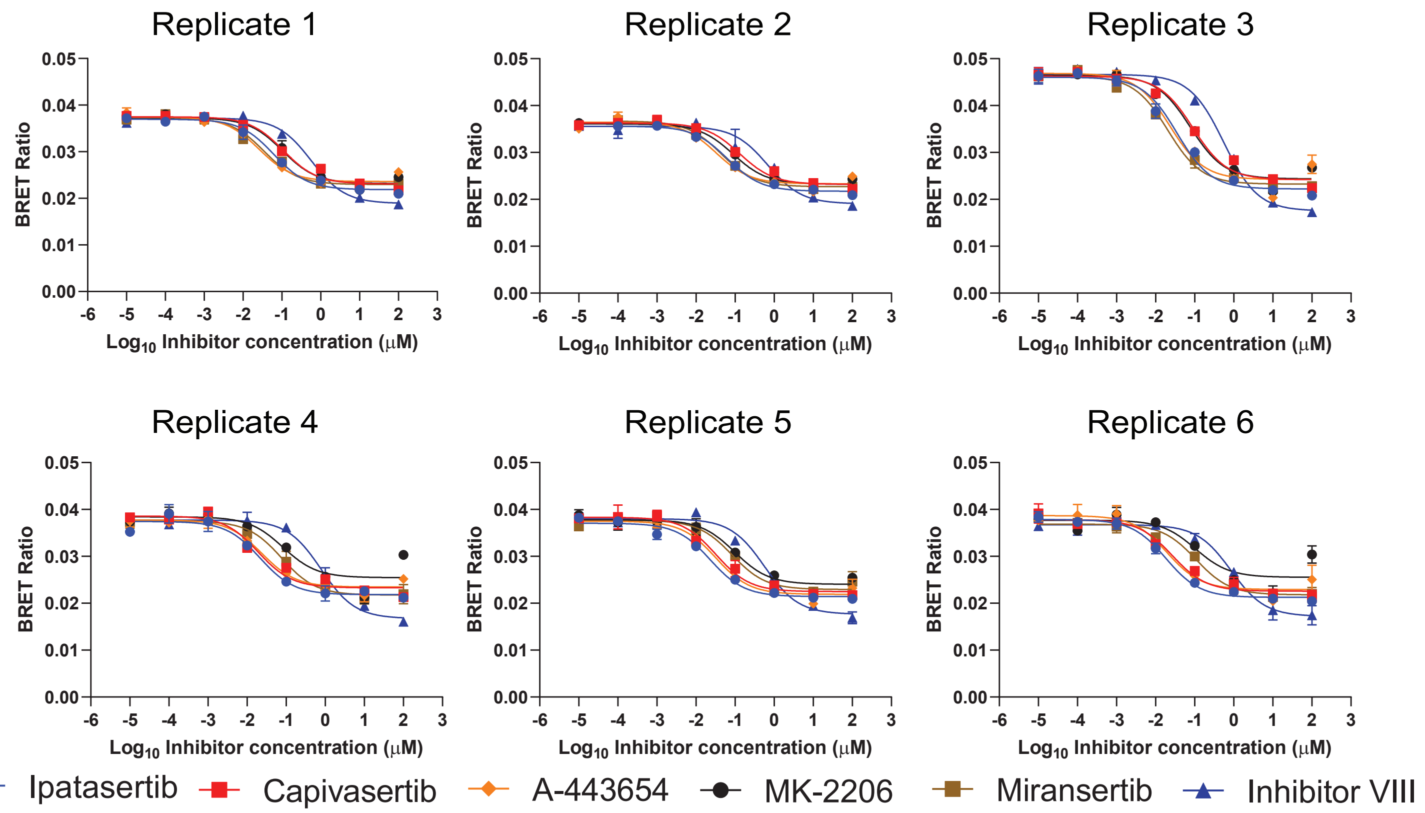

Apparent IC_{50} values

MDA-MB-468 Myr-AKT1-NL	Replicate 1 (μM)	Replicate 2 (μM)	Replicate 3 (μM)	Replicate 4 (μM)	Replicate 5 (μM)	Replicate 6 (μM)	Average (μM)	Standard Error
Ipatasertib	0.0622	0.0636	0.0353	0.0216	0.0234	0.0186	0.0375	0.0084
Capivasertib	0.1090	0.1310	0.0904	0.0192	0.0323	0.0266	0.0681	0.0196
A-443654	0.0261	0.0330	0.0235	0.0240	0.0320	0.0197	0.0264	0.0021
MK-2206	0.1037	0.0885	0.0772	0.0829	0.0982	0.1046	0.0925	0.0046
Miransertib	0.0348	0.0444	0.0205	0.0771	0.0839	0.1081	0.0615	0.0137
Inhibitor VIII	0.5721	0.6862	0.5597	0.7862	0.5679	0.8550	0.6712	0.0517

Calculated K_i Values

MDA-MB-468								
Myr-AKT1-NL	Replicate 1 (nM)	Replicate 2 (nM)	Replicate 3 (nM)	Replicate 4 (nM)	Replicate 5 (nM)	Replicate 6(nM)	Average (nM)	Standard Error
Ipatasertib	17.88	18.28	10.14	6.21	6.71	5.33	10.76	2.41
Capivasertib	31.31	37.63	25.97	5.50	9.28	7.64	19.56	5.63
A-443654	7.49	9.46	6.75	6.90	9.18	5.65	7.57	0.61
MK-2206	29.79	25.43	22.17	23.81	28.21	30.05	26.58	1.33
Miransertib	10.01	12.75	5.89	22.14	24.11	31.05	17.66	3.92
Inhibitor VIII	164.33	197.11	160.77	225.83	163.12	245.59	192.79	14.85

Figure S10- Replicate data associated with Figure 6A and B. **(A)** Signal build-up curves and fitted EC_{50} values using the K5 tracer on MDA-MB-231 or MDA-MB-468 cells transfected with AKT1-NL or Myr-AKT1-NL mutants. **(B)** Replicate binding curves for AKT inhibitors on AKT1-NL overexpressed in MDA-MB-231 cells. Fitted IC_{50} values from binding curves. Calculated K_i values using fitted IC_{50} values and EC_{50} values from panel (A). **(C)** same as (B) for overexpressed Myr-AKT1-NL in MDA-MB-231 cells. **(D)** same as (B) for overexpressed AKT1-NL in MDA-MB-468 cells. **(E)** same as (B) for overexpressed Myr-AKT1-NL in MDA-MB-468 cells.
